# Supplementary material for: An integrative phylogenetic approach for inferring relationships of fossil gobioids (Teleostei: Gobiiformes)
Source: PLoS One. 2022 Jul 8;17(7):e0271121. doi: 10.1371/journal.pone.0271121 (PMC9269936; doi:10.1371/journal.pone.0271121)
Supplement: S1 Appendix — (DOCX) [file pone.0271121.s001.docx]

**Appendix 1: Brief descriptions of fossil species used**

**†*Carlomonnius quasigobius* Bannikov & Carnevale, 2016**

*Material*. Skeleton with otolith imprint.

*Holotype*. Museo Civico di Storia Naturale, Verona; MCSNV IGVR71187 (only known specimen).

*Stratigraphy and distribution*. Lower Eocene of Italy (upper Ypresian; Monte Bolca, Pesciara cave site), 56–47.8 Ma, which makes it currently the oldest skeleton-based gobioid..

*Short description*. Vertebrae number 24 (10 abdominal, 14 caudal); dorsal fin continuous with seven spines and 11 rays, pterygiphore formula 2-3111 (for the spines); anal fin with one spine and seven rays; three anal fin pterygiophores inserting before the haemal spine of the first caudal vertebra; endopterygoid present; five branchiostegals.

*Remarks*. Based on these and a unique combination of other osteological characters Bannikov and Carnevale [1] refrained from assigning *C. quasigobius* to a gobioid family and left it incertae sedis among Gobioidei.

*Reference*. Bannikov & Carnevale [1].

**†*Eleogobius brevis* (Agassiz, 1839)**

*Material*. Skeletons, partially with otoliths *in situ*.

*Holotype*. not assigned (see [2], Tome IV Atlas, Tab. 32, Fig. 2–4).

*Stratigraphy and distribution*. Lower-Middle Miocene of Austria (Eibiswald), Germany (Öhningen, Rhinegraben), Slovakia (Závod), 23–11.6 Ma.

*Short description.* 10 to 12 abdominal and 15 to 17 (18?) caudal vertebrae; first dorsal fin with six spines; second dorsal fin with one spine and 10 to 11 rays; anal fin with one spine and eight to nine rays; six branchiostegals; palatine T-shaped; endopterygoid absent; otolith rectangular with broad posterodorsal projection, small ventral projections anteriorly and posteriorly.

*Reference*. Gierl & Reichenbacher [3].

**†*Eleogobius gaudanti* Gierl & Reichenbacher, 2015**

*Material*. Skeletons, partially with otoliths *in situ*.

*Holotype*. Naturhistorisches Museum Wien; NMW 1857 XXVII 7 (with otoliths in situ).

*Stratigraphy and distribution*. Uppermost lower Miocene of Germany (Illerkirchberg), c. 17 Ma.

*Short description*. 10 abdominal and 16 to 17 caudal vertebrae; first dorsal fin with 6 spines; second dorsal fin with one spine and nine to ten rays; anal fin with one spine and ten to 11 rays; six branchiostegals; palatine T-shaped; endopterygoid absent; otolith rectangular with posterodorsal and anteroventral projection.

*Remarks*. The authors proposed, based on the combination of characters, for this genus to be „in between“ of 6brG and 5brG and that it might be a sister taxon to the 5brG. Bradić-Milinović et al. [4] recognized that the first two branchiostegals are shifted anteriorly on the slender part of the ceratohyal, this might be a hint that this species belongs to another (extinct) genus.

*Reference*. Gierl & Reichenbacher [3].

**†**“***Gobius***” ***francofurtanus* Koken, 1891**

*Material*. Skeletons, partially with otoliths *in situ*.

*Holotype*. see Koken [5] (132–133, Pl. VI, Figs. 7–7a; otolith only); better picture of same otolith in Malz ([6], tab. 4, fig. 51), here designated as lectotype with number Senckenberg-Museum Frankfurt SMF P.1956b.

*Stratigraphy and distribution*. Lower Miocene of Germany (Mainz Basin, Hanau Basin, Upper Rhinegraben), 23–15.9 Ma.

*Short description*. 10 to 11 abdominal and 16 to 18 caudal vertebrae; first dorsal fin with six spines; pterygiophore formula 3-22110; second dorsal fin with one spine and 10 to 11 rays; anal fin with one spine and 9 to 10 rays; five branchiostegals; palatine T-shaped; no endopterygoid; otolith rectangular with big projections at the ventral corners; dorsal corners with slightly smaller projections; sulcus centered.

*Remarks*. Probably a member of the Gobiidae, but uncertain if it belongs to the genus *Gobius*, therefore the genus name of *francofurtanus* is indicated as “*Gobius*”.

*References*: Weiler [7]; Gierl [8]

**†*Gobius jarosi* Přikryl & Reichenbacher, 2018**

*Material*. Skeletons.

*Holotype*. Moravian Museum in Brno; MZM Ge25032a and b.

*Stratigraphy and distribution*. Lower Miocene of the Czech Republic and Poland, 19.1–20.4 Ma.

*Short description*. Vertebral column comprising 11 abdominal and 16–17 caudal vertebrae; first dorsal fin with six spines and pterygiophore formula 3-22110; second dorsal with one spine and 12 rays; anal fin has one spine and 11 rays; two anal pterygiophores anterior to first haemal spine. T-shaped palatine; endopterygoid absent; five branchiostegals. The latter characters place the species within the 5brG. The presence of a premaxilla with a postmaxillary process, the D1 pterygiophore pattern, the numbers of vertebrae and the presence of two anal pterygiophores anterior to the haemal spine of the first caudal vertebra led Reichenbacher et al. [9] to the assignment of *G. jarosi* to the Gobiidae.

*Remarks*. Based on the combination of vertebra number, ray number in the second dorsal fin, and the D1 pterygiophore formula, this new fossil species was assigned to the genus *Gobius*. It is the earliest verified record of the genus.

*References*. Reichenbacher et al. [9]; Kovalchuk et al. [10].

**†*Lepidocottus aries* (Agassiz, 1839)**

*Material*. Skeletons, partially with otoliths *in situ*.

*Holotype*. not assigned and lost (see [2] Tome IV Atlas, Tab. 18, Fig. 3; apparently never printed by mistake).

*Stratigraphy and distribution.* Upper Oligocene of France (Aix-en-Provence; Alpes-de-Haute-Provence), 27.8–23 Ma.

*Short description.* Vertebral column with 10 abdominal and 15–16 caudal vertebrae; first dorsal fin with six spines; corresponding pterygiophore formula 4-22110; second dorsal fin with one spine and ten rays; anal fin with one spine and eight to nine rays; three or four anal pterygiophores anterior to first haemal spine. L-shaped palatine; endopterygoid absent; six branchiostegals. Otolith rectangular longer than high, with sulcus shifted anteriorly.

*Remarks*. Those features together with D1 pterygiophore formula, squamation, and reduced number of principle caudal rays (13) led Gierl et al. [11] to the conclusion that *Lepidocottus* can most likely belongs to be placed within the Butidae.

*References*. Gierl et al. [11]; Gaudant [12]; Pandolfi et al [13].

**†*Paralates bleicheri* Sauvage, 1883**

*Material*. Skeletons.

*Holotype*. Naturhistorisches Museum Basel, NMB Ruf. 15b (neotype designated by Gaudant [14]; type material lost).

*Stratigraphy and distribution*. Lower Oligocene of France (Upper Alsace, Rouffach), 33.9–27.8 Ma.

*Short description*. 12 abdominal and 17 to 18 caudal vertebrae; first dorsal fin with seven spines; second dorsal fin with one spine and 8 to 10 rays; anal fin with one spine and 8 to 10 rays; postmaxillary process on premaxilla present.

*Remarks*. Probably belongs in member of the 6brG clade. It is currently not possible to assign it to a family.

*References*. Gaudant [14]; Gierl & Reichenbacher [15].

**†*Paralates chapelcorneri* Gierl & Reichenbacher, 2017**

*Material*. Skeletons.

*Holotype*. Natural History Museum, London; NHMUK PV P 59785 (part and counterpart)

*Stratigraphy and distribution*. Upper Eocene of the Isle of Wight (UK), 37.8–33.9 Ma.

*Short description*. 12 abdominal and 17 to 19 caudal vertebrae; first dorsal fin with seven spines; pterygiophore formula 3-2121100; second dorsal fin with one spine and 9 to 10 rays; anal fin with one spine and eight to nine rays; postmaxillary process on premaxilla present; long caudal peduncle; six branchiostegals.

*Remarks*. These features of †*Paralates* speak for a placement within the 6brG clade. However, it is currently not possible to assign it to a family.

*References*. Gaudant and Quayle [16]; Gierl and Reichenbacher [15].

**†*Pirskenius diatomaceus* Obrhelová, 1961**

*Material*. Skeletons.

*Holotype*. Národní Museum Prague, NMP PC 2769.

*Stratigraphy and distribution*. Lower Oligocene of the Czech Republic (Knížecí), 33.9–27.8 Ma.

*Short description*. 11 abdominal and 16–17(18) caudal vertebrae; first dorsal fin with seven spines; D1 pterygiophore formula 3-122110 and 3-12210; second dorsal fin with one spine and nine to ten rays; anal fin with one spine and nine to ten rays; precaudal anal pterygiophores 2–3; palatine with short ethmoid process; endopterygoid present; seven branchiostegals.

*Remarks*. Together with †*Pirskenius radoni* (see below), the species is placed in the extinct family †Pirskeniidae, which was recovered as sister to Thalasseleotridae + 5brG in Reichenbacher et al. [17].

*References*. Obrhelová [18]; Přikryl [19]; Reichenbacher et al. [17].

**†*Pirskenius radoni*** **Přikryl, 2014**

*Material*. Skeletons.

*Holotype*. Museum of Teplice; MT PA1480.

*Stratigraphy and distribution*. Lower Oligocene of the Czech Republic (Central Bohemian Uplands), 33.9–27.8 Ma.

*Short description*. 12 abdominal and 16 caudal vertebrae; first dorsal fin with seven spines; D1 pterygiophore formula 4-32110; second dorsal fin with one spine and eight rays; anal fin with one spine and nine rays; precaudal anal pterygiophores 4; palatine „not exactly L-shaped“ (Reichenbacher et al. [17], p. 21); endopterygoid present; seven branchiostegals.

*Remarks*. Together with †*Pirskenius diatomaceus* (see above), the species is placed in the extinct family †Pirskeniidae, which was recovered as sister to Thalasseleotridae + 5brG in Reichenbacher et al. [17].

*References*. Přikryl [19]; Reichenbacher et al. [17].

**References**

1. Bannikov AF, Carnevale G. †*Carlomonnius quasigobius* gen. et sp. nov.: the first gobioid fish from the Eocene of Monte Bolca, Italy. Bulletin of Geosciences. 2016;91(1):13–22. doi: 10.3140/bull.geosci.1577.

2. Agassiz L. Recherches sur les Poissons fossiles. Tome IV. Neuchâtel: Petitpierre; 1833–1843. 348 p.

3. Gierl C, Reichenbacher B. A new fossil genus of Gobiiformes from the Miocene characterized by a mosaic set of characters. Copeia. 2015;103(4):792–805. doi: 10.1643/ci-14-146.

4. Bradić-Milinović K, Ahnelt H, Rundić L, Schwarzhans W. The lost freshwater goby fish fauna (Teleostei, Gobiidae) from the early Miocene of Klinci (Serbia). Swiss Journal of Palaeontology. 2019;138(2):285–315. doi: 10.1007/s13358-019-00194-4.

5. Koken E. Neue Untersuchungen an tertiären Fisch-Otoloithen II. Zeitschrift der deutschen geologischen Gesellschaft. 1891;43:77–170.

6. Malz H. Vergleichend-morphologische Untersuchungen an aquitanen Fisch-Otolithen aus dem Untergrund von Frankfurt am Main. Senckenbergiana lethaea. 1978;59(4/6):441–81.

7. Weiler W. Die Fischfauna des Tertiärs im oberrheinischen Graben, des Mainzer Beckens, des unteren Maintals und der Wetterau, unter besonderer Berücksichtigung des Untermiozäns. Abhandlungen der Senckenbergischen Naturforschenden Gesellschaft. 1963;504:1–75.

8. Gierl C. Articulated gobioid skeletons from the Frankfurt-Formation (Lower Miocene). München: Ludwig-Maximilians-Universität München; 2012.

9. Reichenbacher B, Gregorová R, Holcová K, Šanda R, Vukić J, Přikryl T. Discovery of the oldest *Gobius* (Teleostei, Gobiiformes) from a marine ecosystem of Early Miocene age. Journal of Systematic Palaeontology. 2018;16(6):493–513. doi: 10.1080/14772019.2017.1313323.

10. Kovalchuk O, Świdnicka E, Stefaniak K. A new record of *Gobius jarosi* (Teleostei, Gobiidae) from the Early Miocene of Poland with inference to paleogeography and palaeoecology of the Carpathian Basin. Historical Biology. 2019;31(10):1394–401. doi: 10.1080/08912963.2018.1457032.

11. Gierl C, Reichenbacher B, Gaudant J, Erpenbeck D, Pharisat A. An extraordinary gobioid fish fossil from southern France. PloS one. 2013;8(5):e64117. doi: 10.1371/journal.pone.0064117.

12. Gaudant J. Présence du genre *Lepidocottus* Sauvage, 1875 (Teleostei, Gobioidei) dans l'Oligocène inférieur des environs de Céreste (Alpes-de-Haute-Provence, France). Geodiversitas. 2015;37(2):229–35. doi: 10.5252/g2015n2a4.

13. Pandolfi L, Carnevale G, Costeur L, Del Favero L, Fornasiero M, Ghezzo E, et al. Reassessing the earliest Oligocene vertebrate assemblage of Monteviale (Vicenza, Italy). Journal of Systematic Palaeontology. 2017;15(2):83–127. doi: 10.1080/14772019.2016.1147170.

14. Gaudant J. Sur la présence de Gobiidae (Poissons téléostéens) dans l'Oligocène inférieur de Rouffach (Haut-Rhin). Sci Géol Bull. 1979;32(3):131–7. doi: 10.3406/sgeol.1979.1560.

15. Gierl C, Reichenbacher B. Revision of so-called *Pomatoschistus* (Gobiiformes, Teleostei) from the late Eocene and early Oligocene. Palaeontologia Electronica. 2017;20.2.33A:1–17. doi: 10.26879/721.

16. Gaudant J, Quayle WJ. New palaeontological studies on the Chapelcorner fish bed (Upper Eocene, Isle of Wight). Bull Br Mus nat Hist (Geol). 1988;44(1):15–39.

17. Reichenbacher B, Přikryl T, Cerwenka AF, Keith P, Gierl C, Dohrmann M. Freshwater gobies 30 million years ago: New insights into character evolution and phylogenetic relationships of †Pirskeniidae (Gobioidei, Teleostei). PloS one. 2020;15(8):e0237366. doi: 10.1371/journal.pone.0237366.

18. Obrhelová N. Vergleichende Osteologie der tertiären Süsswasserfische Böhmens (Gobioidei). Sbornik Paläont. 1961;26:103–92.

19. Přikryl T. A new species of the sleeper goby (Gobioidei, Eleotridae) from the České Středohoří Mountains (Czech Republic, Oligocene) and analysis of the validity of the family Pirskeniidae. Paläontol Z. 2014;88(2):187–96. doi: 10.1007/s12542-013-0188-y.
