# Supplementary material for: An integrative phylogenetic approach for inferring relationships of fossil gobioids (Teleostei: Gobiiformes)
Source: PLoS One. 2022 Jul 8;17(7):e0271121. doi: 10.1371/journal.pone.0271121 (PMC9269936; doi:10.1371/journal.pone.0271121)
Supplement: S1 File — Maximum Parsimony trees with bootstrap values and 50% majority-rule consensus (MRC) Bayesian trees with posterior probabilities based on the different data sets used in this study. (DOCX) [file pone.0271121.s003.docx]

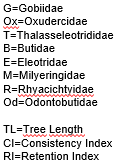

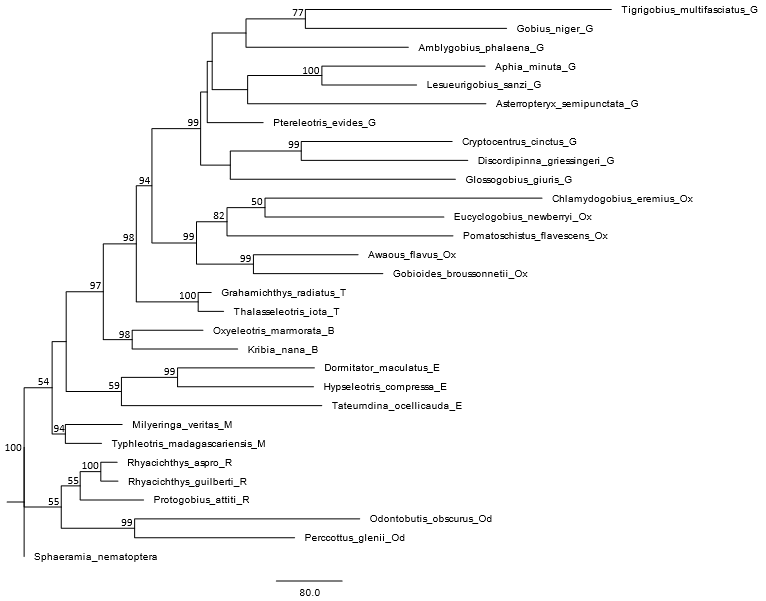


**Fig** **S1** Maximum Parsimony tree with bootstrap values based on published DNA data of the 29 gobioid species used in this study (single tree retained; for sources of molecular data see Table 1). TL = 10112, CI = 0.407, RI = 0.377.

**
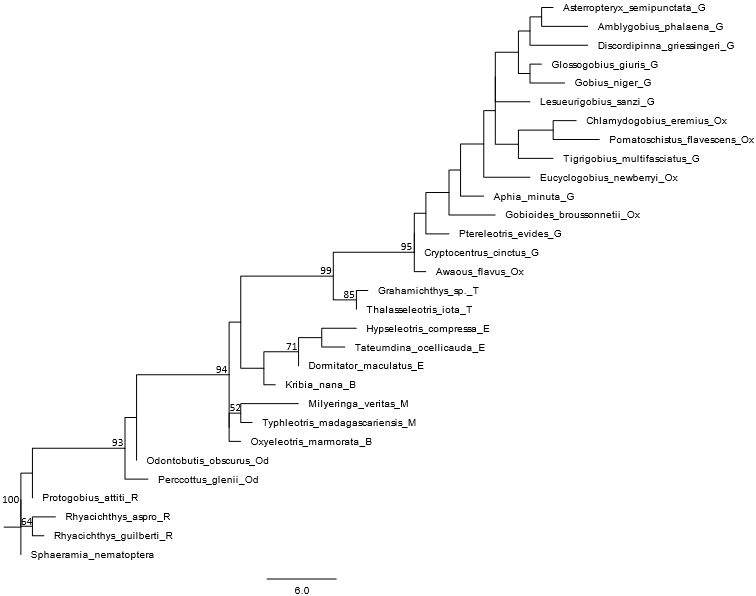
**

**Fig** **S2** Maximum Parsimony tree with bootstrap values based on morphological data of the extant species only (single tree retained). TL = 179, CI = 0.559, RI = 0.782.

**Fig S3** 50% MRC Bayesian tree with posterior probabilities based on a total evidence approach (morphology and DNA) based on the extant species only (ASDSF = 0.000521).

**
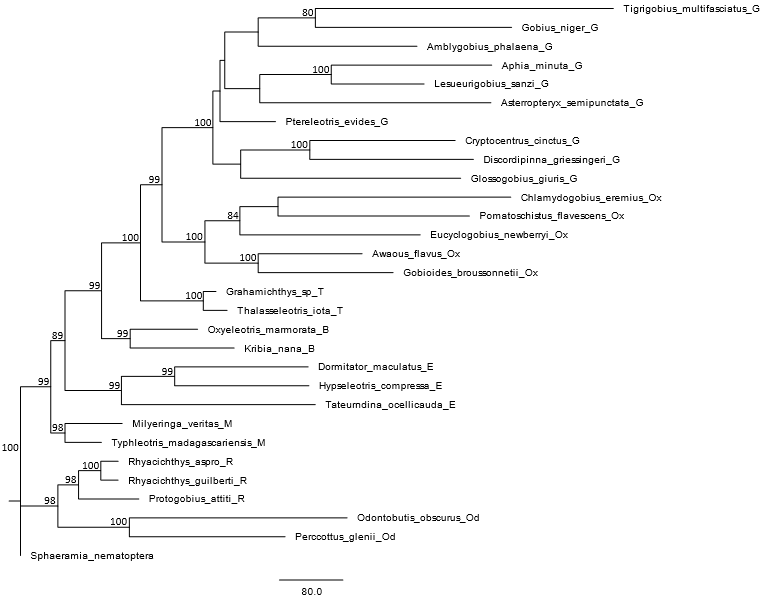
**

**Fig S4** Maximum Parsimony tree with bootstrap values based on a total evidence approach (morphology and DNA) of the extant species only (single tree retained). TL = 10312, CI = 0.409, RI = 0.390.


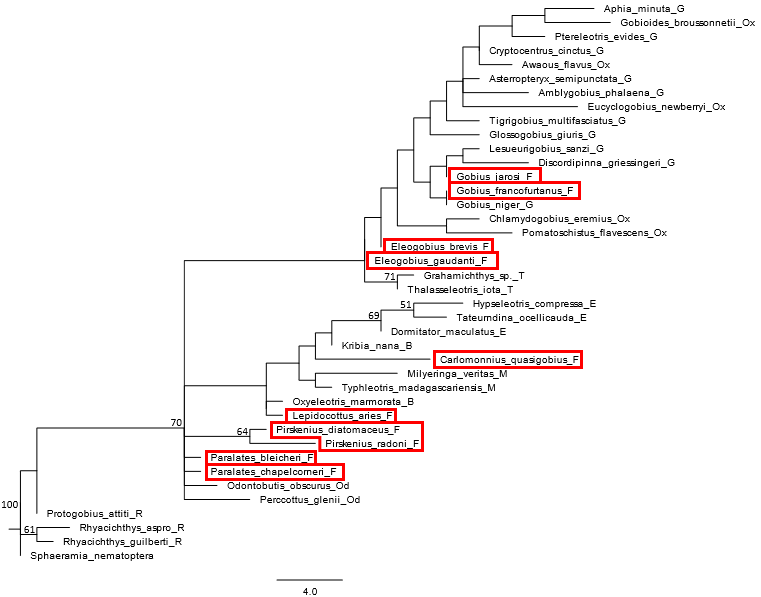


**Fig S5** Maximum Parsimony tree with bootstrap values based on morphological data (50% majority rule consensus of six trees) of the extant species plus ten fossil species (indicated with red frames). TL = 227, CI = 0.467, RI = 0.715.


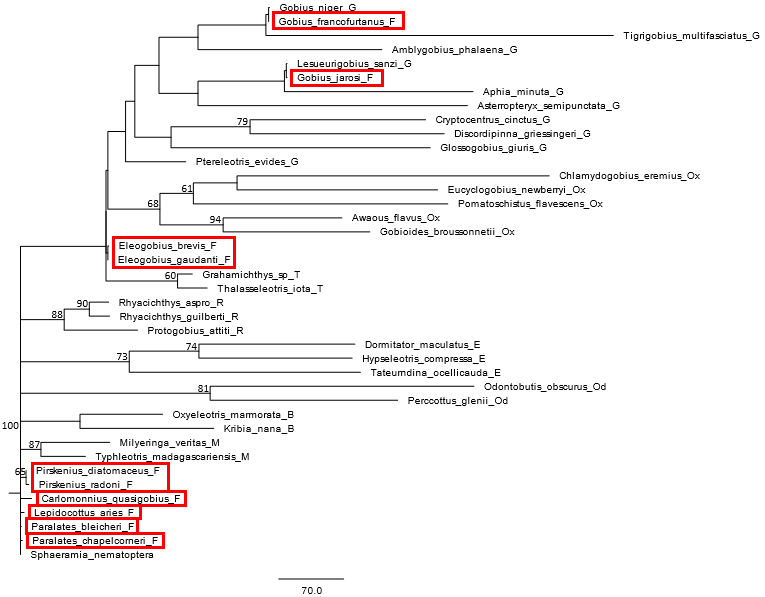


**Fig S6** Maximum Parsimony tree with bootstrap values based on a total evidence approach (morphology and DNA) of the extant species plus all ten fossil species included in this study (50% majority rule consensus of two trees). TL = 10633, CI = 0.397, RI = 0.362.

**
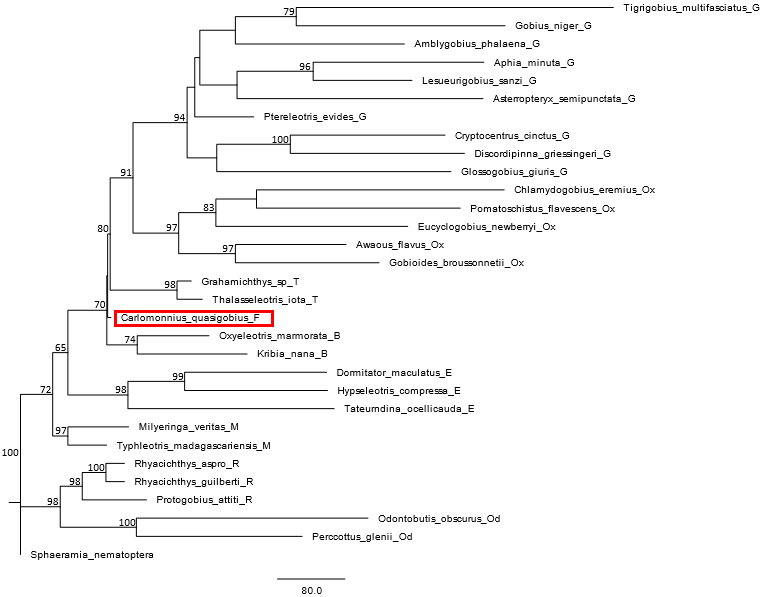
**

**Fig** **S7** Maximum Parsimony tree with bootstrap values based on a total evidence approach (morphology and DNA) of the extant species plus †*Carlomonnius quasigobius* (single tree retained). TL = 10321, CI = 0.409, RI = 0.390.


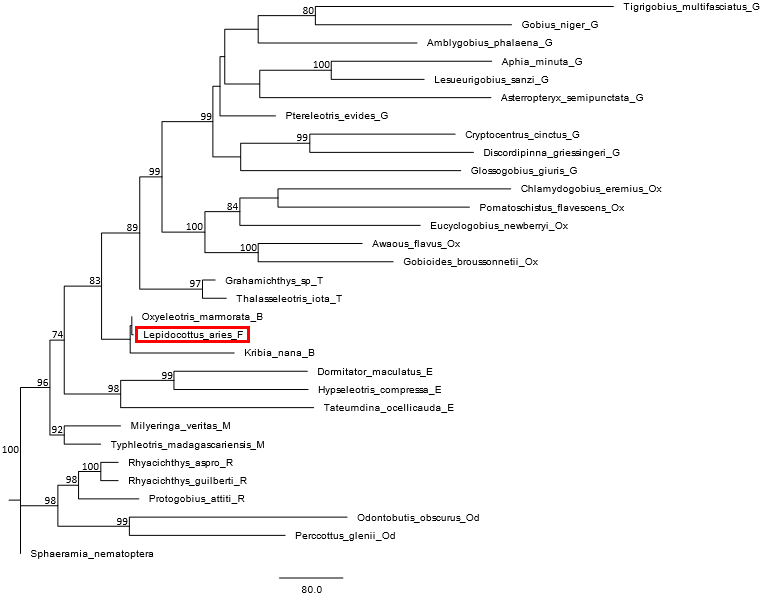


**Fig S8** Maximum Parsimony tree with bootstrap values based on a total evidence approach (morphology and DNA) of the extant species plus †*Lepidocottus aries* (single tree retained). TL = 10316, CI = 0.409, RI = 0.390.


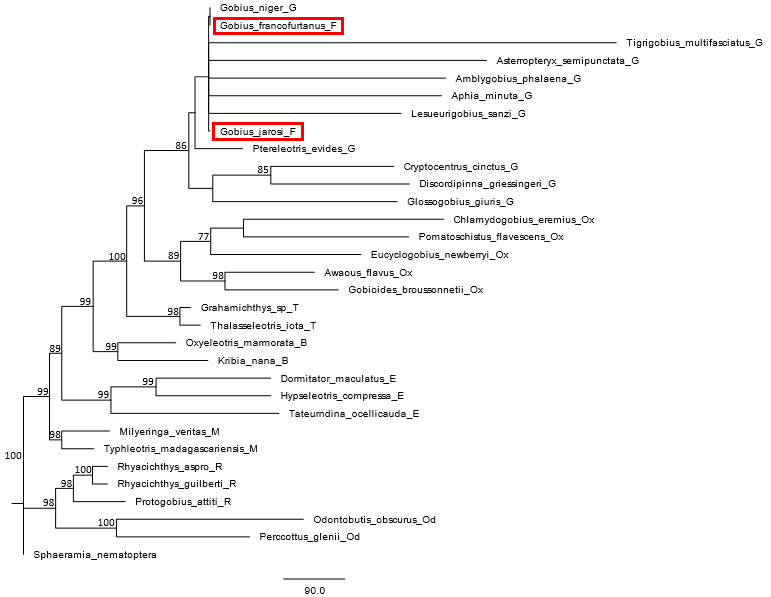


**Fig S9** Maximum Parsimony tree with bootstrap values based on a total evidence approach (morphology and DNA) of the extant species plus †“*Gobius*” *francofurtanus* and †*Gobius jarosi* (50% majority rule consensus tree of two trees). TL = 10623, CI = 0.397, RI = 0.359.

**
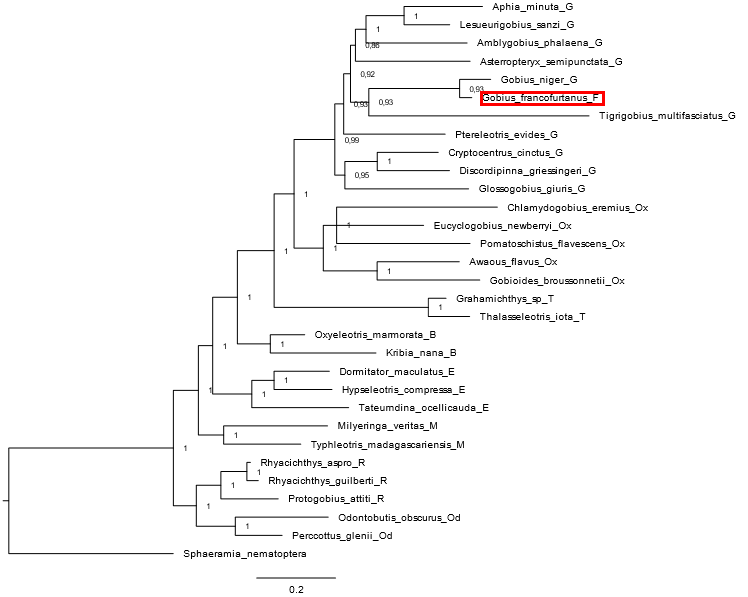
**

**Fig** **S10** 50% MRC Bayesian tree with posterior probabilities based on a total evidence approach (morphology and DNA) using the extant species and adding †*Gobius francofurtanus* (ASDSF = 0.005129).


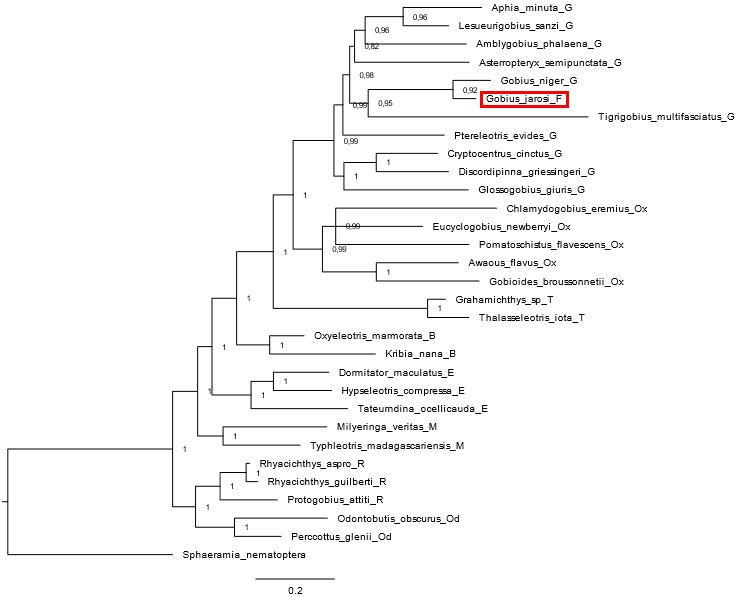


**Fig S11** 50% MRC Bayesian tree with posterior probabilities based on a total evidence approach (morphology and DNA) using the extant species and adding †*Gobius jarosi* (ASDSF = 0.002010).

**
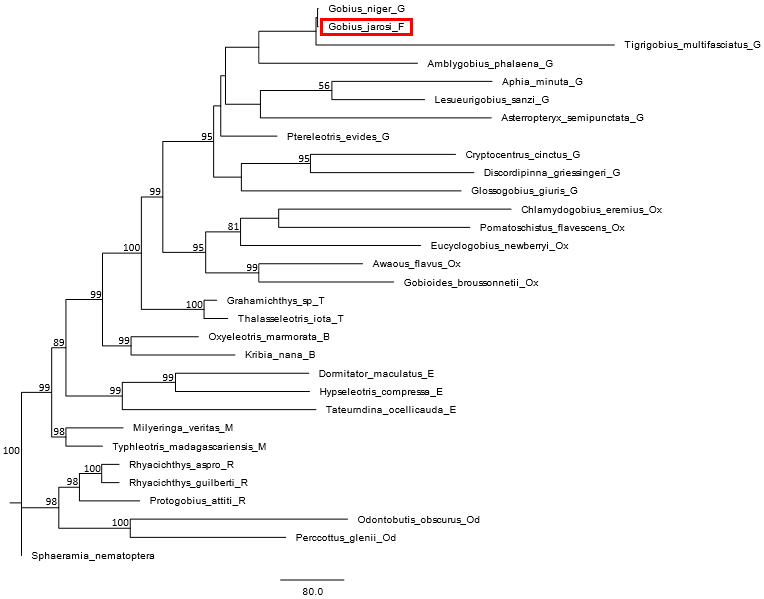
**

**Fig S12** Maximum Parsimony tree with bootstrap values based on a total evidence approach (morphology and DNA) of the extant species plus †*Gobius jarosi* (single tree retained). TL = 10313, CI = 0.409, RI = 0.390.


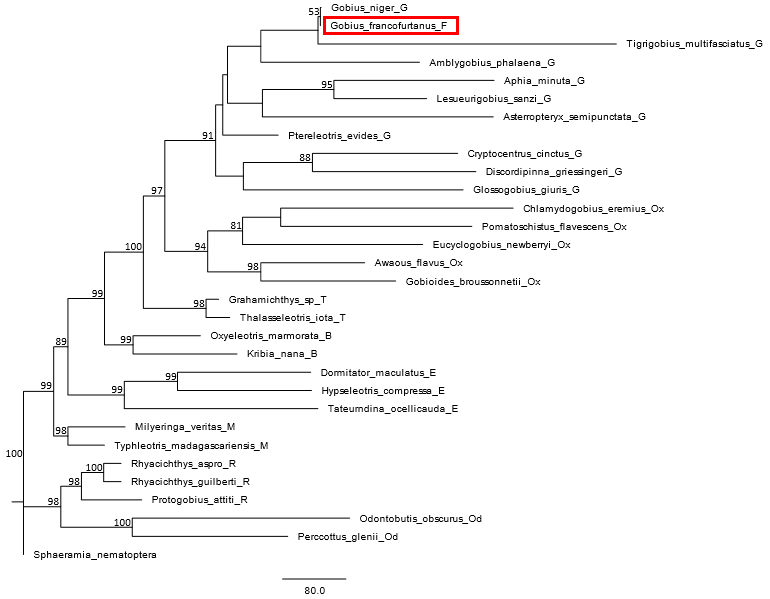


**Fig S13** Maximum Parsimony tree with bootstrap values based on a total evidence approach (morphology and DNA) of the extant species plus †*Gobius francofurtanus* (single tree retained). TL = 10312, CI = 0.409, RI = 0.390.

**Fig S14** 50% MRC Bayesian tree with posterior probabilities based on a total evidence approach (morphology and DNA) using the extant species and †*Eleogobius brevis* (ASDSF = 0.001331).

**Fig S15** 50% MRC Bayesian tree with posterior probabilities based on a total evidence approach (morphology and DNA) using the extant species and †*Eleogobius gaudanti* (ASDSF = 0.000569).

**
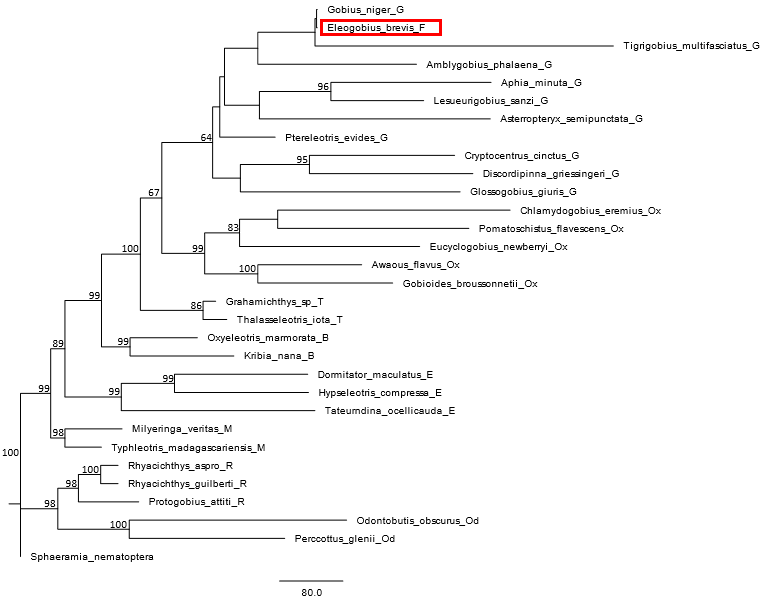
**

**Fig S16** Maximum Parsimony tree with bootstrap values based on a total evidence approach (morphology and DNA) of the extant species plus †*Eleogobius brevis* (single tree retained). TL = 10314, CI = 0.409, RI = 0.390.


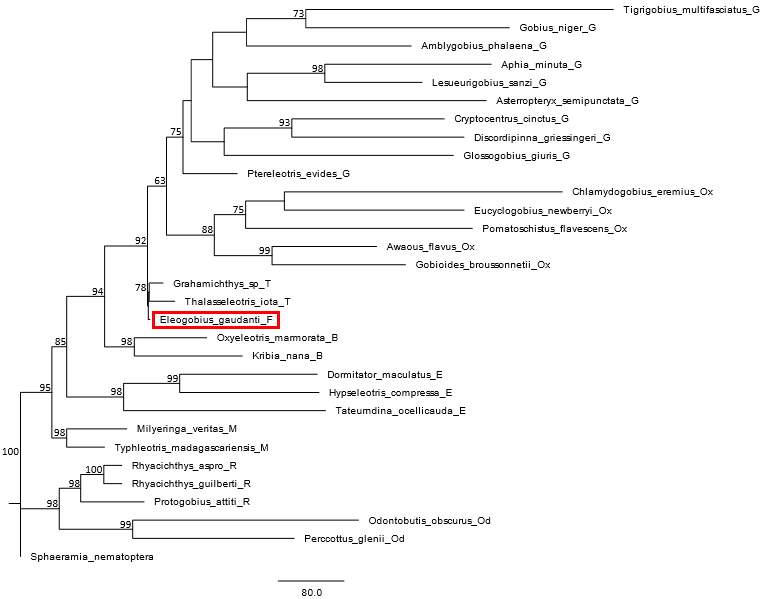


**Fig S17** Maximum Parsimony tree with bootstrap values based on a total evidence approach (morphology and DNA) of the extant species plus †*Eleogobius gaudanti* (single tree retained). TL = 10315, CI = 0.409, RI = 0.390.

**
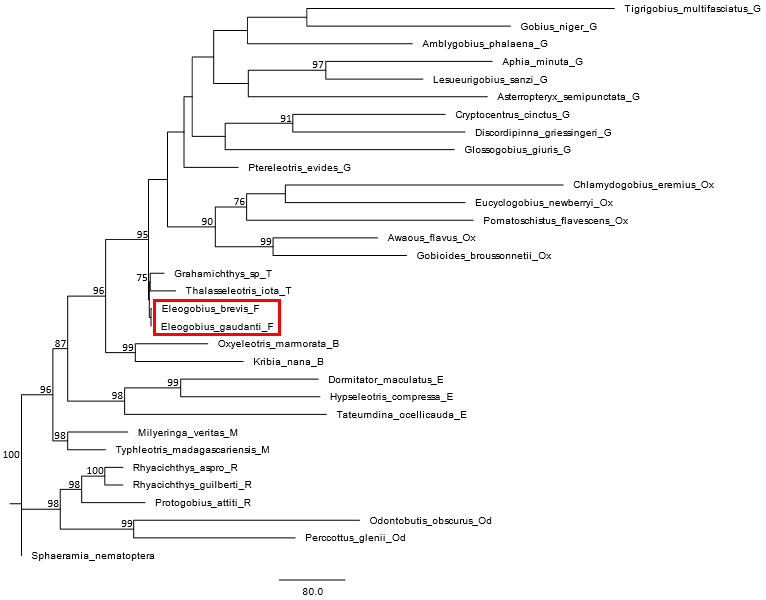
**

**Fig S18** Maximum Parsimony tree with bootstrap values based on a total evidence approach (morphology and DNA) of the extant species plus both †*Eleogobius* species (single tree retained). TL = 10317, CI = 0.409, RI = 0.390.

**Fig S19** 50% MRC Bayesian tree with posterior probabilities based on a total evidence approach (morphology and DNA) using the extant species and †*Pirskenius radoni* (ASDSF = 0.006008).

**Fig S20** 50% MRC Bayesian tree with posterior probabilities based on a total evidence approach (morphology and DNA) using the extant species and †*Pirskenius diatomaceus* (ASDSF = 0.003438).


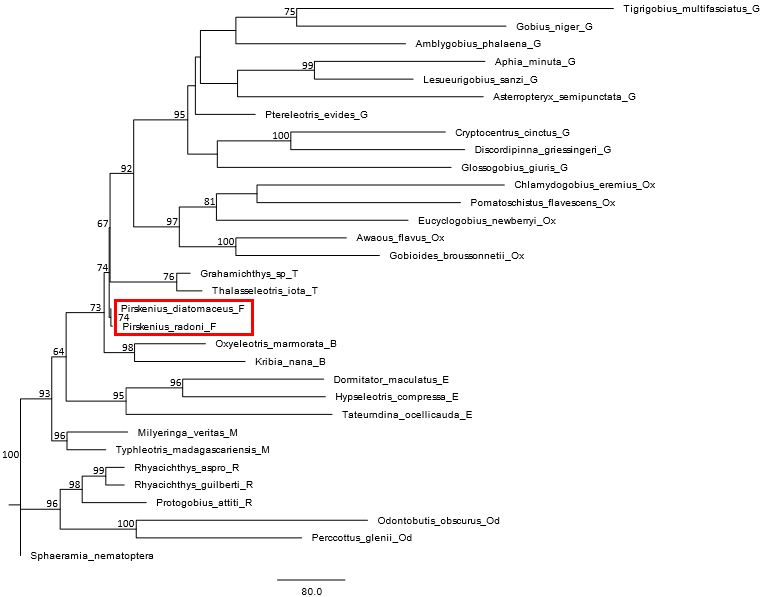


**Fig** **S21** Maximum Parsimony tree with bootstrap values based on a total evidence approach (morphology and DNA) of the extant species plus both †*Pirskenius* species (single tree retained). TL = 10324, CI = 0.409, RI = 0.390.


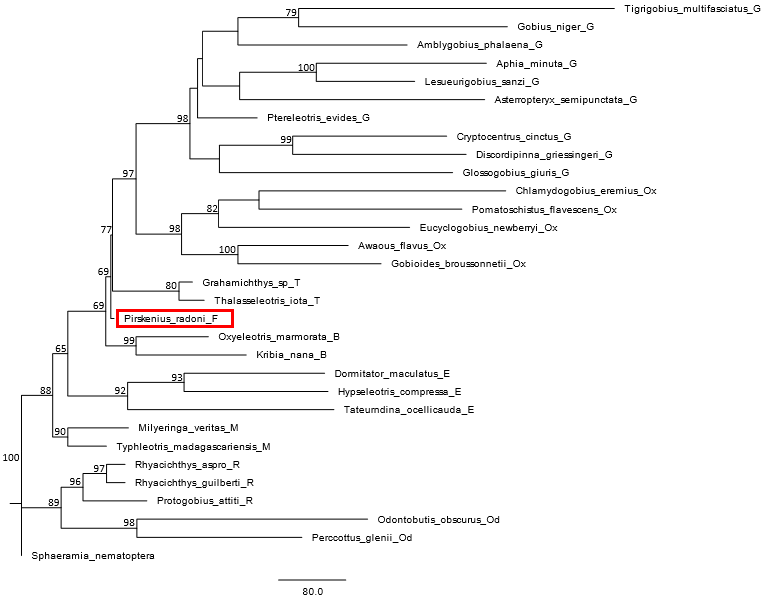


**Fig** **S22** Maximum Parsimony tree with bootstrap values based on a total evidence approach (morphology and DNA) of the extant species plus †*Pirskenius radoni* (single tree retained). TL = 10320, CI = 0.409, RI = 0.390.

**
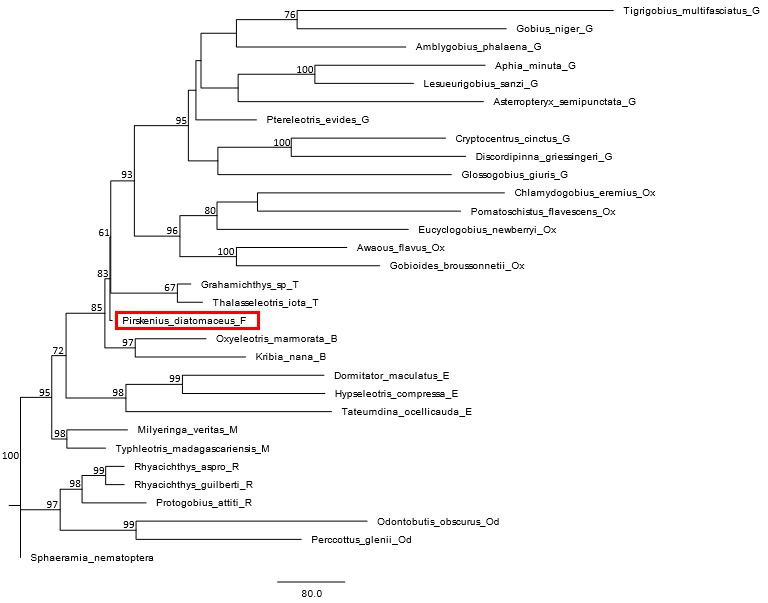
**

**Fig** **S23** Maximum Parsimony tree with bootstrap values based on a total evidence approach (morphology and DNA) of the extant species plus †*Pirskenius diatomaceus* (single tree retained). TL = 10318, CI = 0.409, RI = 0.390.

**Fig** **S24** 50% MRC Bayesian tree with posterior probabilities based on a total evidence approach (morphology and DNA) using the extant species and adding †*Paralates bleicheri* (ASDSF = 0.002460).

**Fig** **S25** 50% MRC Bayesian tree with posterior probabilities based on a total evidence approach (morphology and DNA) using the extant species and adding †*Paralates chapelcorneri* (ASDSF = 0.008340).

**
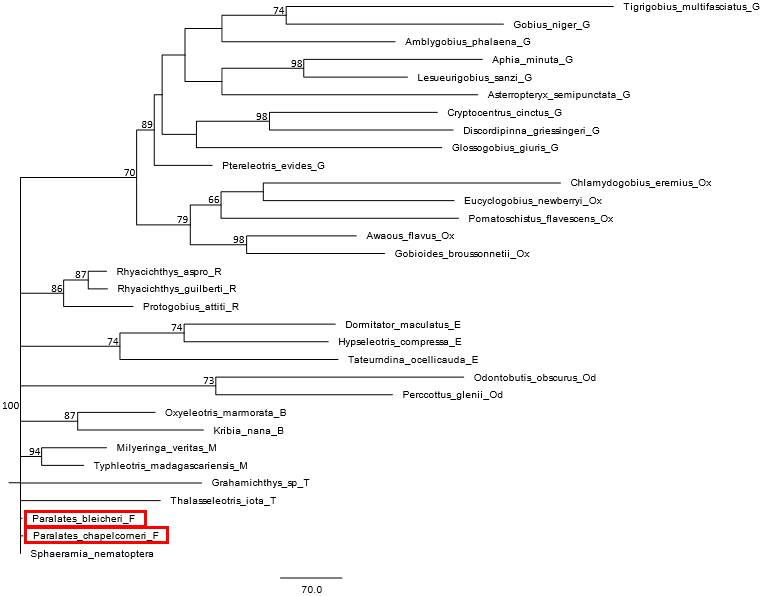
**

**Fig S26** Maximum Parsimony tree with bootstrap values based on a total evidence approach (morphology and DNA) of the extant species plus both †*Paralates* species (50% majority rule consensus of two trees). TL = 10824, CI = 0.390, RI = 0.339.

**
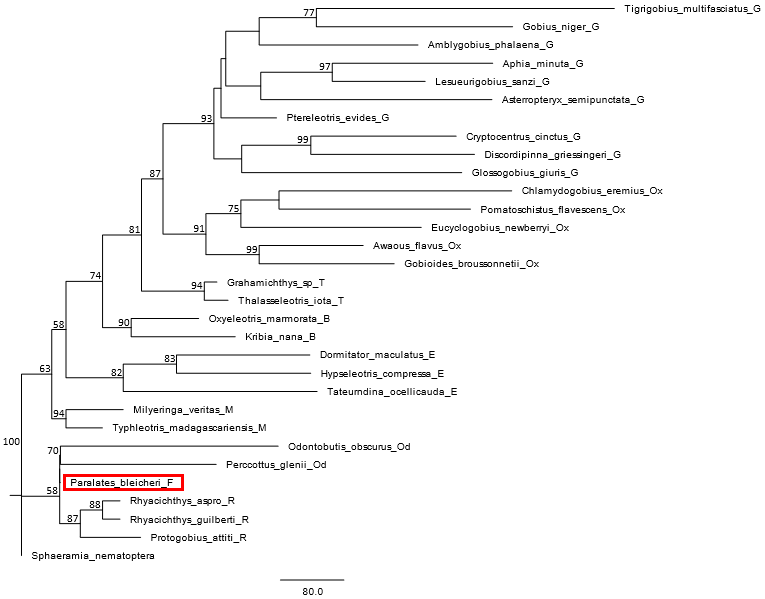
**

**Fig S27** Maximum Parsimony tree with bootstrap values based on a total evidence approach (morphology and DNA) of the extant species plus †*Paralates bleicheri* (single tree retained). TL = 10313, CI = 0.409, RI = 0.390.

**
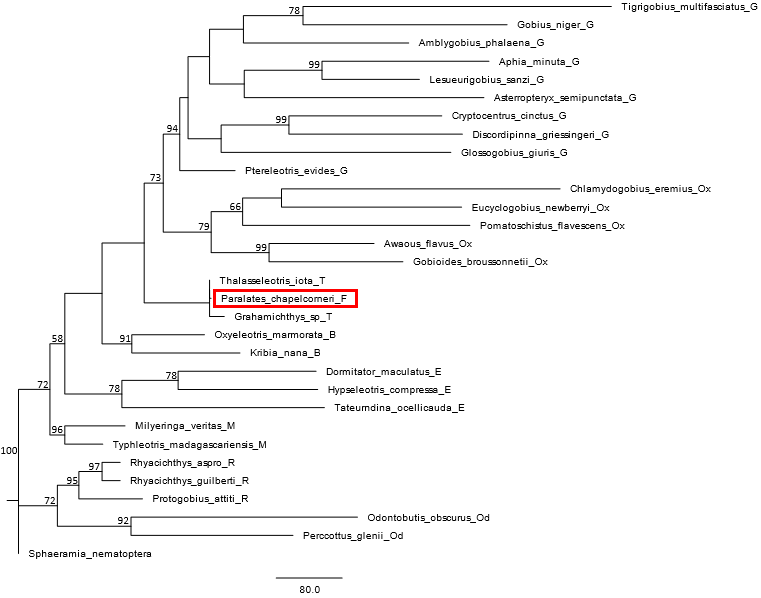
**

**Fig S28** Maximum Parsimony tree with bootstrap values based on a total evidence approach (morphology and DNA) of the extant species plus †*Paralates chapelcorneri* (single tree retained). TL = 10316, CI = 0.409, RI = 0.390.

**Fig S29** 50% MRC Bayesian tree with posterior probabilities based on a total evidence approach (morphology and DNA) using the extant species and adding the two species of †*Paralates* and the two species of †*Pirskenius* (ASDSF = 0.008077). Scale bars, average number of substitutions per site.


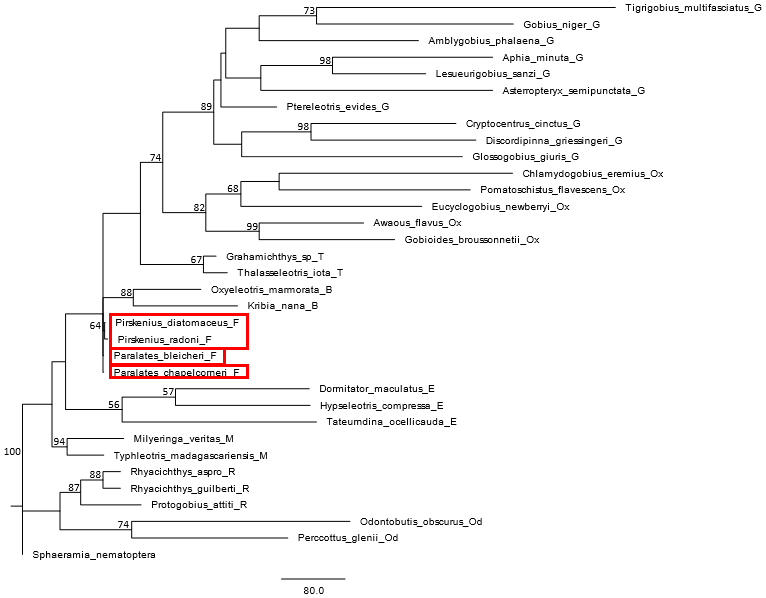


**Fig S30** Maximum Parsimony tree with bootstrap values based on a total evidence approach (morphology and DNA) of the extant species plus the species of †*Pirskenius* and †*Paralates* (50% majority rule consensus of seven trees). TL = 10333, CI = 0.408, RI = 0.389.
