# Supplementary material for: An integrative phylogenetic approach for inferring relationships of fossil gobioids (Teleostei: Gobiiformes)
Source: PLoS One. 2022 Jul 8;17(7):e0271121. doi: 10.1371/journal.pone.0271121 (PMC9269936; doi:10.1371/journal.pone.0271121)
Supplement: Attachment — Submitted filename: PONE-D-21-29896_R1-WWS.pdf [file pone.0271121.s009.pdf]

PLOS ONE

An integrative phylogenetic approach for inferring relationships of fossil gobioids (Teleostei: Gobiiformes)

--Manuscript Draft--

**Manuscript Number:**	PONE-D-21-29896R1
**Article Type:**	Research Article
**Full Title:**	An integrative phylogenetic approach for inferring relationships of fossil gobioids (Teleostei: Gobiiformes)
**Short Title:**	Inferring relationships of fossil gobioids
**Corresponding Author:**	Bettina Reichenbacher<br>Ludwig-Maximilians-Universität München: Ludwig-Maximilians-Universitat Munchen<br>Munich, GERMANY
**Keywords:**	Gobioidei; fossils; molecular characters; morphological characters; total-evidence phylogeny
**Abstract:**	The suborder Gobioidei is among the most diverse groups of vertebrates, comprising about 2310 species. In the fossil record gobioids date back to the early Eocene (c. 50 m.y. ago), and a considerable increase in numbers of described species is evident since the middle Miocene (c. 16 m.y. ago). About 40 skeleton-based gobioid species and > 100 otolith-based species have been described until to date. However, assignment of a fossil gobioid species to specific families has often remained tentative, even if well preserved complete specimens are available. The reasons are that synapomorphies that can be recognized in a fossil skeleton are rare (or absent) and that no phylogenetic framework applicable to fossils exists. Here we aim to overcome this problem by developing a phylogenetic total evidence framework that is suitable to place a fossil skeleton-based gobioid species at family level. Using both literature and newly collected data we assembled a morphological character matrix (48 characters) for 29 extant species, representing all extant gobioid families, and ten fossil gobioid species, and we compiled a multi-gene concatenated alignment (supermatrix; 6271 bp) of published molecular sequence data for the extant species. Bayesian and Maximum Parsimony analyses revealed that our selection of extant species was sufficient to achieve a molecular 'backbone' that fully conforms to previous molecular work. Inclusion of a single fossil species in the total evidence data set of the extant species provided new insight in its possible placement at family level, especially in a Bayesian framework. Five out of the ten fossil species were recovered in the same family as had been suggested in previous works based on comparative morphology. The remaining five fossil species had hitherto been left as family incertae sedis . Now, based on our phylogenetic framework, new and mostly well supported hypotheses to which clades they could belong can be presented. Additionally, the extinct genera † Eleogobius and † Paralates were recovered as paraphyletic and should be revised in future works. We conclude that the total evidence framework presented here will be beneficial for all future work dealing with the phylogenetic placement of a fossil skeleton-based gobioid and thus will help to improve our understanding of the evolutionary history of these fascinating fishes.
**Order of Authors:**	Christoph Gierl
	Martin Dohrmann
	Philippe Keith
	William Humphreys
	Hamid Reza Esmaeili
	Jasna Vukíc
	Radek Šanda
	Bettina Reichenbacher
**Response to Reviewers:**	Responses to specific reviewer comments are provided in the file "Response to

Powered by Editorial Manager® and ProduXion Manager® from Aries Systems Corporation

	Reviewers".<br>Response to the comments of the Editor are also included in the file "Response to Reviewers".
**Additional Information:**	
**Question**	**Response**
**Financial Disclosure**<br><br>Enter a financial disclosure statement that describes the sources of funding for the work included in this submission. Review the submission guidelines for detailed requirements. View published research articles from *PLOS ONE* for specific examples.<br><br>This statement is required for submission and **will appear in the published article** if the submission is accepted. Please make sure it is accurate.<br><br>**Unfunded studies**<br>Enter: *The author(s) received no specific funding for this work.*<br><br>**Funded studies**<br>Enter a statement with the following details:<br>• Initials of the authors who received each award<br>• Grant numbers awarded to each author<br>• The full name of each funder<br>• URL of each funder website<br>• Did the sponsors or funders play any role in the study design, data collection and analysis, decision to publish, or preparation of the manuscript?<br>• **NO** - Include this sentence at the end of your statement: *The funders had no role in study design, data collection and analysis, decision to publish, or preparation of the manuscript.*<br>• **YES** - Specify the role(s) played.<br><br>* typeset	BR received funding from the Deutsche Forschungsgemeinschaft (grant number RE-1113/20). RS received funding from the Ministry of Culture of the Czech Republic (grant number DKRVO 2019-2023/6.III.d National Museum, 00023272).<br>https://www.dfg.de/<br>https://www.mkcr.cz/<br>The funders had no role in study design, data collection and analysis, decision to publish, or preparation of the manuscript.
**Competing Interests**<br><br>Use the instructions below to enter a competing interest statement for this submission. On behalf of all authors, disclose any competing interests that could be perceived to bias this work—acknowledging all financial support	The authors have declared that no competing interests exist.

and any other relevant financial or non-financial competing interests.<br><br>This statement is required for submission and **will appear in the published article** if the submission is accepted. Please make sure it is accurate and that any funding sources listed in your Funding Information later in the submission form are also declared in your Financial Disclosure statement.<br><br>View published research articles from *PLOS ONE* for specific examples.<br><br>**NO authors have competing interests**<br><br>Enter: *The authors have declared that no competing interests exist.*<br><br>**Authors with competing interests**<br><br>Enter competing interest details beginning with this statement:<br><br>*I have read the journal's policy and the authors of this manuscript have the following competing interests: [insert competing interests here]*<br><br>* typeset	
**Ethics Statement**<br><br>Enter an ethics statement for this submission. This statement is required if the study involved:<br><br>• Human participants<br>• Human specimens or tissue<br>• Vertebrate animals or cephalopods<br>• Vertebrate embryos or tissues<br>• Field research<br><br>Write "N/A" if the submission does not require an ethics statement.<br><br>General guidance is provided below.	All specimens originate from museum collections (see Table 1) and no specimens were sacrificed for this work.<br>No permits were required for the described study, which complied with all relevant regulations.

Powered by Editorial Manager® and ProduXion Manager® from Aries Systems Corporation

Consult the submission guidelines for detailed instructions. **Make sure that all information entered here is included in the Methods section of the manuscript.**<br><br>**Format for specific study types**<br><br>**Human Subject Research (involving human participants and/or tissue)**<br>• Give the name of the institutional review board or ethics committee that approved the study<br>• Include the approval number and/or a statement indicating approval of this research<br>• Indicate the form of consent obtained (written/oral) or the reason that consent was not obtained (e.g. the data were analyzed anonymously)<br><br>**Animal Research (involving vertebrate animals, embryos or tissues)**<br>• Provide the name of the Institutional Animal Care and Use Committee (IACUC) or other relevant ethics board that reviewed the study protocol, and indicate whether they approved this research or granted a formal waiver of ethical approval<br>• Include an approval number if one was obtained<br>• If the study involved *non-human primates*, add *additional details* about animal welfare and steps taken to ameliorate suffering<br>• If anesthesia, euthanasia, or any kind of animal sacrifice is part of the study, include briefly which substances and/or methods were applied<br><br>**Field Research**<br><br>Include the following details if this study involves the collection of plant, animal, or other materials from a natural setting:<br>• Field permit number<br>• Name of the institution or relevant body that granted permission	
**Data Availability**<br><br>Authors are required to make all data underlying the findings described fully available, without restriction, and from the	Yes - all data are fully available without restriction

time of publication. PLOS allows rare exceptions to address legal and ethical concerns. See the PLOS Data Policy and FAQ for detailed information.<br><br>A Data Availability Statement describing where the data can be found is required at submission. Your answers to this question constitute the Data Availability Statement and **will be published in the article**, if accepted.<br><br>**Important:** Stating 'data available on request from the author' is not sufficient. If your data are only available upon request, select 'No' for the first question and explain your exceptional situation in the text box.<br><br>Do the authors confirm that all data underlying the findings described in their manuscript are fully available without restriction?	
**Describe where the data may be found in full sentences. If you are copying our sample text, replace any instances of XXX with the appropriate details.**<br><br>• If the data are **held or will be held in a public repository**, include URLs, accession numbers or DOIs. If this information will only be available after acceptance, indicate this by ticking the box below. For example: *All XXX files are available from the XXX database (accession number(s) XXX, XXX.*).<br>• If the data are all contained **within the manuscript and/or Supporting Information files**, enter the following: *All relevant data are within the manuscript and its Supporting Information files.*<br>• If neither of these applies but you are able to provide **details of access elsewhere**, with or without limitations, please do so. For example:<br><br>*Data cannot be shared publicly because of [XXX]. Data are available from the XXX Institutional Data Access / Ethics Committee (contact via XXX) for*	All relevant data are within the manuscript and its Supporting Information files. The data underlying the results presented in the study are available from https://figshare.com/s/ed10b9a5ac382f856a20

researchers who meet the criteria for access to confidential data.<br><br>*The data underlying the results presented in the study are available from (include the name of the third party and contact information or URL).*<br>• This text is appropriate if the data are owned by a third party and authors do not have permission to share the data.<br><br>* typeset	
Additional data availability information:	Tick here if the URLs/accession numbers/DOIs will be available only after acceptance of the manuscript for publication so that we can ensure their inclusion before publication.

Powered by Editorial Manager® and ProduXion Manager® from Aries Systems Corporation

Manuscript
Click here to access/download;Manuscript;Gierl et al Manuscript.docx

An integrative phylogenetic approach for inferring relationships of fossil gobioids (Teleostei: Gobiiformes)

Christoph Gierl[1], Martin Dohrmann[1], Philippe Keith[2], William Humphreys[3], Hamid R. Esmaeili[4], Jasna Vukić[5], Radek Šanda[6], Bettina Reichenbacher[1,7]*

[1]Department of Earth and Environmental Sciences, Palaeontology & Geobiology, Ludwig-Maximilians-Universität München, Munich, Germany

[2]UMR 8067 Biologie des organismes et écosystèmes aquatiques (BOREA), Muséum national d'Histoire naturelle, CNRS, IRD, SU, Paris, France

[3]School of Biological Sciences, University of Western Australia, Crawley, WA 6009, Australia; Western Australian Museum, 49 Kew Street, Welshpool, WA 6106, Australia

[4]Ichthyology and Molecular Systematics Research Laboratory, Zoology Section, Department of Biology, College of Sciences, Shiraz University, Shiraz, Iran

[5]Department of Ecology, Charles University, Prague, Czech Republic

[6]Department of Zoology, National Museum, Prague, Czech Republic

[7]GeoBio-Center LMU, Ludwig-Maximilians-Universität München, Munich, Germany

Corresponding author: Bettina Reichenbacher. E-mail: b.reichenbacher@lrz.uni-muenchen.de

Abstract

The suborder Gobioidei is among the most diverse groups of vertebrates, comprising about 2310 species. In the fossil record gobioids date back to the early Eocene (c. 50 m.y. ago), and a considerable increase in numbers of described species is evident since the middle Miocene (c. 16 m.y. ago). About 40 skeleton-based gobioid species and > 100 otolith-based species have been described until to date. However, assignment of a fossil gobioid species to specific families has often remained tentative, even if well preserved complete specimens are available. The reasons are that synapomorphies that can be recognized in a fossil skeleton are rare (or absent) and that no phylogenetic framework applicable to fossils exists. Here we aim to overcome this problem by developing a phylogenetic total evidence framework that is suitable to place a fossil skeleton-based gobioid species at family level. Using both literature and newly collected data we assembled a morphological character matrix (48 characters) for 29 extant species, representing all extant gobioid families, and ten fossil gobioid species, and we compiled a multi-gene concatenated alignment (supermatrix; 6271 bp) of published molecular sequence data for the extant species. Bayesian and Maximum Parsimony analyses revealed that our selection of extant species was sufficient to achieve a molecular 'backbone' that fully conforms to previous molecular work. Inclusion of a single fossil species in the total evidence data set of the extant species provided new insight in its possible placement at family level, especially in a Bayesian framework. Five out of the ten fossil species were recovered in the same family as had been suggested in previous works based on comparative morphology. The remaining five fossil species had hitherto been left as family *incertae sedis*. Now, based on our phylogenetic framework, new and mostly well supported hypotheses to which clades they could belong can be presented. Additionally, the extinct genera †*Eleogobius* and †*Paralates* were recovered as paraphyletic and should be revised in future works. We conclude that the total evidence framework presented here will be beneficial for all

future work dealing with the phylogenetic placement of a fossil skeleton-based gobioid and thus will help to improve our understanding of the evolutionary history of these fascinating fishes.

Introduction

The suborder Gobioidei (Percomorpha: Gobiiformes) is among the most diverse groups of fishes, encompassing about 320 genera and about 2310 species [1, 2]. Gobioids live in marine, brackish, and freshwater habitats, and form an important component of reef faunas [3-5]. They show a wide range of specializations, including the ability to survive on land for long periods of time (mudskippers; *e.g.* [6]), being diadromous and alternating freshwater and sea water during their lifecycle [7], and forming symbioses with certain shrimp species [8, 9].

Gobioidei belongs to Gobiiformes (*sensu* Thacker et al. [10]), together with Kurtidae, Apogonidae, and Trichonotidae, and consists of nine families: Rhyacichthyidae, Odontobutidae, Milyeringidae, Eleotridae, Butidae, Thalasseleotrididae, Gobiidae, and Oxudercidae (= formerly Gobionellidae [1]), and the extinct †Pirskeniidae [11] (Fig 1). Phylogenetic relationships within extant Gobioidei are generally well resolved based on molecular data [10, 12, 13]. Morphologically, the extant Gobioidei can be divided into two major groups based on the number of branchiostegal rays (bones supporting the gill membrane [14, 15]). Possession of five branchiostegal rays is considered to be the derived state within Gobioidei, supporting the clade Gobiidae + Oxudercidae (denoted 5brG here; see Fig 1), which is also recovered on the basis of molecular data [10, 12, 13]. In contrast, Gobioidei with six branchiostegal rays (6brG) form a paraphyletic group in molecular phylogenies [12, 16], indicating that this condition is plesiomorphic within Gobioidei. The only gobioid that has seven branchiostegal rays, of which the last ray is expanded as is typical for Gobioidei (see [17]), is the genus †*Pirskenius* (denoted 7brG here), and phylogenetic

analyses placed it within the paraphyletic 6brG [11]; see Fig 1. The closest living relatives of Gobioidei, the Apogonidae, Kurtidae, and Trichonotidae, all have seven branchiostegals and no expansion of the last branchiostegal ray [1, 17-19].

Fig 1. Current understanding of gobioid relationships showing the different nomenclatures in use. brG = branchiostegal-rayed Gobioidei.

Previous gobioid classifications date back to more than 100 years ago. Regan [20] had already recognized that two main groups within Gobioidei, which he termed Eleotridae and Gobiidae, can be distinguished based on the configuration of the pelvic fins (separate in Eleotridae vs. united in Gobiidae), the shape of the palatine (L- vs. T-shaped), the endopterygoid (present vs. mostly absent), and the composition of the shoulder girdle (presence vs. absence of the dorsal coracoid [=scapula]). Regan's Eleotridae conforms to the paraphyletic 6brG, and his Gobiidae are reflected in the 5brG. Since then, several efforts have been made to classify groups within Gobioidei using morphological characters (see [21] for a comprehensive compilation). Akihito [22] and Hoese [23] noted that the loss of the anteriormost branchiostegal ray appears to be characteristic for the most advanced groups in Gobioidei (= 5brG). Miller [24] erected the family Rhyacichthyidae and reclassified Gobioidei into Rhyacichthyidae and Gobiidae, with the latter comprising seven subfamilies, Eleotrinae and Xenisthminae (now Eleotridae), Gobionellinae and Tridentigerinae (now included in Oxudercidae), Gobiinae and Kraemeriinae (now Gobiidae), and †Pirskeniinae (referring to †Pirskeniidae Obrhelová, 1961) [10, 12, 25]. Harrison [26] tried to resolve gobioid interrelationships with the help of features of the palatopterygoquadrate complex and indicated some putative relationships among six taxa of the 5brG. Pezold [27] recognized a distinct head pore configuration as an autapomorphy of the subfamily Gobiinae (now in Gobiidae). Hoese and Gill [28] recognized the family Odontobutidae and used 16 characters

to propose interrelationships among Rhyacichthyidae, Odontobutidae, and Gobiidae; their Gobiidae included the Butinae, Eleotridinae and Gobiinae, each of which is now assigned family rank [12, 29]. Other authors used morphological data for phylogenetic studies of subgroups of various families, as Larson [30] has done for the *Mugilogobius* group of the Gobiidae, and Murdy [31] for the Oxudercinae. Most of the more recent works that include morphological characters have focused on smaller groups within Gobioidei (e.g. [29, 32-36]).

The known fossil record of Gobioidei is relatively modest in comparison to their recent diversity and comprises about 40 species that are known from skeletal material. In older studies, these species have been assigned to genera or families by comparing meristic characters, such as counts of vertebrae and fin rays (e.g. [37-40]). More recent authors have also considered the systematic value of certain osteological features (e.g. shape of the palatine, first dorsal fin-pterygiophore formula, number of branchiostegal rays, configuration of the palatine-ectopterygoid complex, presence of the entopterygoid), which enabled a tentative systematic placement of some fossil gobioids in relation to extant taxa [41-47].

However, determining the systematic context of fossil gobioids remains a challenge. Firstly, fossil gobioids apparently include several extinct lineages (e.g. [42, 43, 45, 48-51]). Secondly, a phylogenetic analysis of extant gobioids based on morphological characters is hampered by the rarity of synapomorphic characters [28], and becomes even more difficult in fossil gobioids, which usually preserve only skeletal traits and otoliths [11]. Thirdly, all comparative approaches suffer from a limited knowledge of the range of skeletal characters of extant gobioids, which is also due to the sheer number of species (currently 2310, see above) and the inaccessibility of some rare taxa [52, 53]. This explains why, with the exception of †Pirskeniidae [11], no phylogenetic analyses have yet been conducted to position fossil gobioids within the phylogeny of extant taxa.

The objective of this study was to place ten selected fossil gobioid species within an integrative ("total evidence") phylogenetic framework including published molecular

sequence data from extant species in combination with morphological data for both fossil and extant species. The choice of fossils was largely based on our previous works (see section Material and Methods). The overall idea was that placement of fossil gobioids in the context of a rigorous phylogenetic analysis would greatly help to improve our understanding of the evolutionary history of these fascinating fishes.

Institutional abbreviations

BMNH, former acronym for British Museum of Natural History (now NHMUK); FMNH, Field Museum of Natural History, Chicago, Illinois, USA; IRSNB, Royal Institute of Natural Sciences, Brussel, Belgium; LACM, Los Angeles County Museum of Natural History, Los Angeles, California, USA; MNHN, Muséum national d'Histoire naturelle, Paris, France; MRAC, Musée royal de l'Afrique centrale, Tervuren, Belgium; NHMUK, Natural History Museum in London, United Kingdom; NMP, National Museum, Prague, Czech Republic; SMNS, State Museum for Natural History, Stuttgart, Germany; WAM, Western Australian Museum, Welshpool, Australia; ZM-CBSU, Zoological Museum Collection of the Biology Department at Shiraz University, Iran; ZSM, Bavarian State Collection of Zoology, Munich, Germany.

Material and Methods

Ethic statement

All specimens originate from museum collections (see Table 1) and no specimens were sacrificed for this work.

Compilation of the taxon set

The ingroup species of the Gobioidei used in this study comprise 29 extant species; the apogonid *Sphaeramia nematoptera* is used as outgroup (Table 1). The Odontobutidae, Milyeringidae, Butidae, and Thalasseleotridae are each represented by two species, the Rhyacichthyidae and the Eleotridae each by three, the Oxudercidae by five, and the Gobiidae by ten species. Criteria for species selection were the availability of 'total-evidence data' including molecular as well as morphological data of the skeleton and otolith data. Morphological data of four species were coded based solely on published information: the odontobutid *Odontobutis obscurus* (data from [44, 54, 55]), the milyeringid *Typhleotris madagascariensis* (data from [56]), the thalasseleotridid *Thalasseleotris iota* (data from [57-59]), and the oxudercid *Eucyclogobius newberryi* (data from [60, 61]); no otolith data were available for *T. madagascariensis*. This selection of extant species, although modest in terms of species numbers of Oxudercidae and Gobiidae, was sufficient to achieve a molecular 'backbone' as its phylogenetic analysis produced a well-resolved tree that fully agrees with published hypotheses (see Results).

Ten fossil gobioid species were added to the extant taxon set (Table 1). We selected those that we had examined in previous works [11, 42-44, 46, 62], and added also the oldest known putative gobioid so far, †*Carlomonnius quasigobius* (Table 1). A short overview of all fossil species is provided in Appendix 1.

Table 1. Species and specimens used in this study. Birdsong et al. [60], Hoese and Gill [28] and Gill and Mooi [15] are used for morphology of many or all species. Colour codes for molecular data: Agorreta et al. [12] =green; Near et al. [63] =blue; Thacker et al. [10] =red; for not color-coded cells see entries in Genbank via given accession numbers.

**Family**	**Species**	**Morphology**	**rRNA**	***cytb***	***rag1***	***zic1***	***sreb2***
Fossil	†*Carlomonnius quasigobius* Bannikov and Carnevale, 2016	Bannikov and Carnevale 2016 [64]	n/a	n/a	n/a	n/a	n/a
Fossil	†*Eleogobius brevis* (Agassiz, 1839)	NHMUK PV OR 42779–80; Gierl and Reichenbacher 2015 [42]	n/a	n/a	n/a	n/a	n/a
Fossil	†*Eleogobius gaudanti* Gierl and Reichenbacher, 2015	Gierl and Reichenbacher 2015 [65]	n/a	n/a	n/a	n/a	n/a
Fossil	†“*Gobius*” *francofurtanus* Koken, 1891	Weiler 1963; Gierl 2012 [62, 66]	n/a	n/a	n/a	n/a	n/a
Fossil	†*Gobius jarosi* Prikryl and Reichenbacher, 2018	Reichenbacher et al. 2018 [46]	n/a	n/a	n/a	n/a	n/a
Fossil	†*Lepidocottus aries* (Agassiz, 1839)	Gierl et al. 2013 [44]	n/a	n/a	n/a	n/a	n/a

Fossil	†*Paralates bleicheri* Sauvage, 1883	Gierl and Reichenbacher 2017 [43]	n/a	n/a	n/a	n/a	n/a
Fossil	†*Paralates chapelcorneri* Gierl and Reichenbacher, 2017	Gierl and Reichenbacher 2017 [43]	n/a	n/a	n/a	n/a	n/a
Fossil	†*Pirskenius diatomaceus* Obrhelová, 1961	Obrhelová 1961; Přikryl 2014; Reichenbacher et al. 2020 [11, 49, 67]	n/a	n/a	n/a	n/a	n/a
Fossil	†*Pirskenius radoni* Přikryl 2014	Přikryl 2014; Reichenbacher et al. 2020 [11, 67]	n/a	n/a	n/a	n/a	n/a
Gobiidae	*Amblygobius phalaena* (Valenciennes, 1837)	ZSM-PIS-P-GO-0594, -0600; Harrison 1989 [26]	KF415301	KF415498	KF415693	KF415897	KF416109
Gobiidae	*Aphia minuta* (Risso, 1810)	NMP 6V; Rojo 1985; Birdsong et al. 1988; Harrison 1989; La Mesa et al. 2005 [26, 60, 68, 69]	KF415305	FR851417	KF415697	KF415901	KF416113
Gobiidae	*Asterropteryx semipunctata* Rüppell, 1830	SMNS-Z-PI-026591, -005882; Birdsong et al. 1988; Van Tassell et al. 1988; Harrison 1989 (skeleton data); Schwarzhans et al. 2020: pl. 7, fig. 5 (otolith) [26, 50, 60, 70]	KF415309	KF415506	KF415701	KF415906	KF416117
Gobiidae	*Cryptocentrus cinctus* (Herre, 1936)	ZSM-PIS-P-GO-0619, -0639; Karplus and Thompson 2011 [9]	KF415340	KF415536	KF415732	KF415940	KF416150
Gobiidae	*Discordipinna griessingeri* Hoese and Fourmanoir, 1978	ZSM-PIS-P-GO-0632, -0633; Hoese and Fourmanoir 1978; Birdsong et al. 1988 [60, 71]	KF415345	KF415540	KF415737	KF415945	KF416155

Gobiidae	*Glossogobius giuris* (Hamilton, 1822)	ZM-CBSU; Harrison 1989; Esmaeili et al. 2009 [26, 72]	KF41 5369	KF41 5566	KF41 5767	KF41 5972	KF41 6185
Gobiidae	*Gobius niger* Linnaeus, 1758	NMP6V 146072, - 146073; Harrison 1989 [26]	KF41 5385	KF41 5583	KF41 5786	KF41 5990	KF41 6203
Gobiidae	*Lesueurigobius sanzi* (de Buen, 1918)	ZSM-PIS-035529_1, _4; Miller 1986; Birdsong et al. 1988; Harrison 1989; McKay and Miller 1997 (skeleton data); Schwarzhans et al. 2020: pl. 2, fig. 3 (otolith) [26, 60, 73, 74]	KF41 5406	KF41 5603	KF41 5808	KF41 6012	
Gobiidae	*Ptereleotris evides* (Jordan and Hubbs, 1925)	ZSM-PIS-P-GO-0587, -0588; Randall and Hoese 1985 [75]			KF14 1341	KF14 0623	KF14 0263
Gobiidae	*Tigrigobius multifasciatus* (Steindachner, 1876)	ZSM-PIS-P-GO-0622, -0642	AF49 1102	AY84 6402	KF41 5878	KF41 6088	KF41 6302
Oxudercidae	*Awaous flavus* (Valenciennes, 1837)	ZSM-PIS-43853 (P-GO-1050, -1051)	KF41 5311	KF41 5508	KF41 5703	KF41 5908	KF41 6119
Oxudercidae	*Chlamydogobius eremius* (Zietz, 1896)	ZSM-PIS-43854 (P-GO-1052, -1053); Miller 1987 [76]	KF41 5329	KF41 5526	KF41 5720	KF41 5928	KF41 6138
Oxudercidae	*Eucyclogobius newberryi* (Girard, 1856)	Birdsong et al. 1988; Kindermann et al. 2007 [60, 61]; otolith from the collection of W. Schwarzhans ex LACM-58239	KF41 5355	EU38 0942	KF41 5751	KF41 5958	KF41 6169
Oxudercidae	*Gobioides broussonnetii* Lacepède, 1800	ZSM-PIS-43852 (P-GO-1048, -1049)	KF41 5374		KF41 5772	KF41 5977	KF41 6189

Oxudercidae	*Pomatoschistus flavescens* (Fabricius, 1779)	ZSM-PIS-043982 (2); Vigo, Spain, Sanda, Prag; Mestermann and Zander 1984; Harrison 1989 [26, 77]	KF415386	KF415584	KF415787	KF415991	KF416204
Thalasseleotrididae	*Grahamichthys* sp.	ZSM-P-GO-0783; Whitley 1956; McDowall 1965; Akihito 1986; Gierl and Reichenbacher 2015 [42, 54, 78-80]			KT266412	KT266520	KT266467
Thalasseleotrididae	*Thalasseleotris iota* Hoese and Roberts, 2005	Hoese and Larson 1987; Hoese and Roberts 2005 (skeleton data); Schwarzhans 2019: fig. 99.10 (otolith) [57-59]			KT266413	KT266521	
Butidae	*Kribia nana* (Boulenger, 1901)	MRAC (1-5); Wongrat 1977 [81]		AY722211	KF235468		
Butidae	*Oxyeleotris marmorata* (Bleeker, 1852)	ZSM-PIS-43857 (P-GO-1058, -1059); Regan 1911; Harrison 1989 [20, 26]	KF415429	KF415623	KF415829	KF416035	KF416250
Eleotridae	*Dormitator maculatus* (Bloch, 1792)	ZSM-PIS-009383 – 85; Harrison 1989 (skeleton data) [26]; otolith from the collection of W. Schwarzhans ex LACM (collection Fitch)	KF415347	KF415542	KF415739	KF415947	KF416157
Eleotridae	*Hypseleotris compressa* (Krefft, 1864)	ZSM-PIS-43863 (P-GO-1068, -1069); Akihito 1986; Akihito et al. 2000; Thacker and Unmack 2005 [14, 25, 54]	KF415398	KF415596	KF415800	KF416004	KF416217
Eleotridae	*Tateurndina ocellicauda* Nichols, 1955	ZSM-PIS-43855 (P-GO-1054, -1055)	KF415480	KF415672	KF415875	KF416085	KF416299

Milyeringidae	*Milyeringa veritas* Whitley, 1945	WAM-BES1151(B), -BES18735, -BES9768.2; Larson et al. 2013 [82]		HM590598	KT266404	KT266511	KT266459
Milyeringidae	*Typhleotris madagascariensis* Petit, 1933	FMNH 116494-98 (skeleton data only); Sparks and Chakrabarty 2012 [56]		JQ619661	KY981273		
Odontobutidae	*Odontobutis obscurus* (Temminck and Schlegel, 1845)	IRSNB ex BMNH 1983.11.4 (otolith only); Iwata et al. 1985; Akihito 1986; Iwata and Sakai 2002; Gierl et al. 2013 [44, 54, 55, 83]	KF415424	KF415618	KF415825	KF416030	KF416245
Odontobutidae	*Perccottus glenii* Dybowski, 1877	ZSM-PIS-43867 (P-GO-1078, -1079); Akihito 1986; Birdsong et al. 1988; Harrison 1989 [26, 54, 60]	KF415440	KF415632	KF415837	KF416044	JX190055
Rhyacichthyidae	*Rhyacichthys aspro* (Valenciennes, 1837)	ZSM-PIS-044262 (BAyFi 13481); Miller 1973; Birdsong et al. 1988 [24, 60]	KF415462	KF415654	KF415858	KF416066	KF416282
Rhyacichthyidae	*Rhyacichthys guilberti* Dingerkus and Séret, 1992	MNHN 2019-0113-1, MNHN 2019-0113-2		KF669052			
Rhyacichthyidae	*Protogobius attiti* Watson and Pöllabauer, 1998	MNHN 2019-0112; Akihito et al. 2000; Shibukawa et al. 2001 [14, 84]		AB021257			
Apogonidae	*Sphaeramia nematoptera* (Bleeker, 1856)	ZSM-PIS-P-GO-0621, -0634; McAllister 1968; Fraser 1972; Bergman 2004 [19, 85, 86]	AB889673		KT266401	KT266508	KT266456

168

Study and compilation of morphological characters

Literature data, when available, were used to compile phylogenetically informative morphological characters for the extant species (see Table 1 for details). Additionally, X-ray images were produced for the extant species with a Faxitron Ultrafocus facility at the ZSM and examined to determine numbers of vertebrae, fin elements and pterygiophores, and configuration of the caudal skeleton. After radiography, otoliths were extracted from the same specimens and prepared for scanning electron microscopy (SEM) imaging (using a HITACHI SU 5000 Schottky FE-SEM at the Department of Earth- and Environmental Sciences, LMU Munich). SEM images served as the basis for the identification of the otolith characters (Fig. 2), which are used here for the first time within a phylogenetic matrix. They include (i) the overall otolith shape (six character states: trapezoid/triangular; long rectangular; high rectangular; quadratic; rounded; longish-ovate), (ii) the posterodorsal projection (two states: present; absent), (iii) the sulcus shape (three states: perciform-like, shoe sole, shoe sole/specialized), and (iv) the sulcus shape and position (three states: shoe sole + centred, shoe sole + shifted anteriorly, not shoe sole). Plesiomorphic otolith character states were defined according to the condition seen in the otolith of the outgroup (see Supplementary Table for details). The otoliths of the included extant gobioid species are shown in Figure 3, the otolith of the outgroup species *Sphaeramia nematoptera* is depicted in Figure 2. For the fossil species, skeletal and otolith characters were largely compiled from previous works, but some additional characters could also be added (see Results).

Fig 2. Otolith terminology and otolith characters and character states used here for the phylogenetic analyses. Images depict left otoliths (sagittae) in medial view. Scale bars: 0.5 mm.

A total of 48 morphological characters were assembled. Thirty-eight characters concern bony structures of the skeleton, four characters relate to cartilage, membrane, or tendon configurations, four refer to otolith morphology, two concern the presence and type of ctenii on the scales, and one is a morphometric character (see Supplementary Table Part B for all characters). Character states were determined according to literature data and our morphological investigations (based on X-rays, SEM images of otoliths) (see Supplementary Table Part A for details).

All taxa and characters were assembled in Mesquite 3.61 [87]. We used presence/absence coding (1/0) or up to ten states, depending on the character (see Supplementary Table).

Preparation of the molecular data matrix

Molecular sequence data for extant species were assembled based on previously published sequences of five markers: *rDNA* (12S rRNA, tRNA-Val, 16S rRNA), *cytb*, *rag1*, *zic1*, and *sreb2*. The sequences were mainly from the study of Agorreta et al. [12], supplemented by some data from Near et al. [63] and Thacker et al. [10]. Molecular data were downloaded from GenBank, aligned in AliView 1.26 [88] with MUSCLE [89], followed by manual adjustment and exclusion of ambiguous regions where necessary. Individual gene alignments were then concatenated into a supermatrix (6271 bp) with SeaView 5.0.4 [90]. For details and GenBank accession numbers see Table 1. All data matrices are available on figshare (https://figshare.com/s/ed10b9a5ac382f856a20).

Phylogenetic analyses

We conducted phylogenetic analyses for the extant species based on the morphological character matrix, the molecular supermatrix, and based on the combined

molecular and morphological (total evidence) datasets. Adding all ten fossils to the total evidence character set resulted in a collapse of the molecular backbone (see below). Therefore we used a step-wise approach: (i) a single fossil species was added, (ii) two fossil species of the same genus were added, (iii) four fossil species were added. We used the Bayesian Markov Chain Monte Carlo (MCMC) approach implemented in MrBayes versions 3.2.6 and 3.2.7a [91], as well as implied-weights maximum parsimony (IW-MP, [92]) implemented in TNT 1.5 [93] to infer phylogenies.

In MrBayes, we assigned the Mkv+G model [94, 95] to the morphological data, and separate GTR+G models [95, 96] to each molecular partition. For each analysis, we ran 2 x 4 MCMC chains in parallel for 5 x 10^6 generations. We used the "sump" command in MrBayes as well as Tracer 1.7.1 [97] to check for convergence and discarded the first 10% of samples of each analysis as burn-in before summarizing the remaining samples in 50% majority-rule consensus (MRC) trees including posterior probability (PP) values for all clades.

In TNT, we employed new-technology searches (with sectorial search, ratchet, drift, and tree fusing enabled; init. addseqs = 100; find min. length = 10) and a concavity constant of K = 12. A 50% majority-rule consensus tree was calculated if more than one most parsimonious tree was found. For assessing clade support in the IW-MP analysis, we used standard bootstrap resampling [98] (500 replicates; new technology search; init. addseq = 10; find min. length = 5). Phylogenetic trees were visualized in FigTree 1.4.4 [99]; tree files are available on figshare (https://figshare.com/s/ed10b9a5ac382f856a20).

Results

Skeleton data

The character state of each character of each species (extant and fossil) is provided in the Supplementary Table; unknown character states were coded with a question mark.

Three characters could be coded for the first time for †*Eleogobis brevis* based on the specimens NHMUK PV OR 42779 and 42780, deposited in the Natural History Museum in London: the pterygiophore formula (3-12210), the presence of a single anal fin pterygiophore inserting before the haemal spine of the first caudal vertebra (AP = 1), and the position of the penultimate branchiostegal on the ceratohyal. Re-inspection of the type specimens of †*Gobius jarosi* revealed that in this species the penultimate branchiostegal is located on the ceratohyal. Finally, a count of three to four anal fin pterygiophores (AP = 3-4) could be determined for †*Lepidocottus aries* based on re-inspection of the specimens used in Gierl et al. [44].

Otolith data

Figure 3 depicts the otoliths of the extant species included in this study. Short descriptions of the otoliths are given below.

Fig 3. Otoliths of the extant gobioid species used in this study. Images depict left otoliths (sagittae) in medial view, except for *Lesueurigobius sanzi*, *Asterropteryx semipunctata*, *Dormitator maculatus* and *Odontobutis obscurus*, which represent right sagittae that were mirrored for better comparison. Otoliths were not available for the milyeringid *Typhleotris madagascariensis*. For sources of otoliths see Table 1. Scale bars: 0.5 mm.

Family Gobiidae

Amblygobius phalaena (Valenciennes, 1837). – High-rectangular, margins crenate, dorsal rim strongly lobed, pronounced posterodorsal projection present. Sulcus centered, shoe-sole shape with slender cauda and wide ostium with dorsal lobe.

Aphia minuta (Risso, 1810). – Rounded, margins smooth, short projection slightly below level of ostium tip. Sulcus shifted anteriorly, rather shoe-sole shape, cauda strongly reduced, ostium with dorsal lobe and pointed tip.

Asterropteryx semipunctata Rüppell, 1830. – Rectangular, higher than long. Dorsal, anterior and ventral margins slightly crenulate, small posterodorsal projection. Sulcus centered, shoe-sole shape, with rounded cauda and ostium. See Schwarzhans et al. [50] for additional details of the same otolith.

Cryptocentrus cinctus (Herre, 1936). – High-rectangular, margins smooth, with a projection in the middle of the dorsal margin and a median constriction on anterior and posterior rims. Sulcus centered, shoe-sole shape with small, rounded cauda and a broad ostium with ostial lobe.

Discordipinna griessingeri Hoese and Fourmanoir, 1978. – Long-rectangular, smooth margins with median constrictions anterior and posterior defining four projections. Sulcus centered, shortened, oval specialized, surrounded by a bulging crista.

Glossogobius giuris (Hamilton, 1822). – Long-rectangular, slightly crenate margins, posterodorsal projection and a sub-median projection on the posterior rim. Sulcus centered, shoe-sole shape, cauda elongated, ostium with strong dorsal lobe.

Gobius niger Linnaeus, 1758. – Long-rectangular, dorsal margin slightly undulated, prominent posterodorsal and posteroventral projections, small anteroventral projection. Sulcus centered, shoe-sole shape, elongated with rounded cauda and ostium with dorsal lobe.

Lesueurigobius sanzi (de Buen 1918) – Quadratic with rounded dorsal margin, dorsal and anterior margins faintly lobed, other margins smooth, submedian constriction on posterior margin. Posterodorsal projection short and rounded, weak anteroventral projection. Sulcus centered, shoe-sole shape, rounded cauda, ostium with pointed tip. See Schwarzhans et al. [50] for additional details of the same otolith.

Ptereleotris evides (Jordan and Hubbs, 1925). – High-rectangular, margins sinuate; small posteroventral and more pronounced anteroventral projection. Sulcus centered, shoe-sole shape, with rounded cauda and ostium.

Tigrigobius multifasciatus (Steindachner, 1876). – Quadratic, margins undulated, cusp on dorsal rim. Sulcus shifted anteriorly, shoe-sole shape, cauda rounded, ostium with pronounced elongate tip.

Family Oxudercidae

Awaous flavus (Valenciennes, 1837). – High-rectangular, dorsal and posterior margins crenate. Sulcus centered, shoe-sole shape. Cauda and ostium rounded, ostium with marked dorsal lobe.

Chlamydogobius eremius (Zietz, 1896). – Quadratic, margins slightly undulated, small posterodorsal projection. Sulcus centered, shoe-sole shape, with small, rounded cauda and broad ostium with marked dorsal lobe.

Eucyclogobius newberryi (Girard, 1856). – Quadratic with slightly curved dorsal margin, small posterodorsal projection, sulcus centered, shoe-sole shape, with well-developed cauda and ostium.

Gobioides broussonnetii Lacepède, 1800. – Rounded, margins irregularly crenate, small posterodorsal cusp. Sulcus centered, shoe-sole shape, rounded cauda, large ostium with rounded tip.

Pomatoschistus flavescens (Fabricius, 1779). – Quadratic, margins smooth. Sulcus centered, shoe-sole shape, weakly developed cauda, rounded ostium.

Family Thalasseleotrididae

Grahamichthys sp. – Rounded, all margins smooth, no projections. Sulcus centered, shoe-sole shape, cauda and ostium rounded.

Thalasseleotris iota Hoese and Roberts, 2005. – Long-rectangular, margins smooth, with prominent praeventral projection and slightly rounded anterodorsal expansion. Sulcus centered, shoe-sole shape, cauda and ostium slender. See Schwarzhans [58] for additional details of the same otolith.

Family Butidae

Kribia cf. *nana* (Boulenger, 1901). – Long-rectangular, all margins relatively smooth, small posterodorsal projection, posteroventral expansion (bulge). Sulcus centered, shoe-sole shape, slightly shifted anteriorly. Rounded cauda about half the length of the ostium.

Oxyeleotris marmorata (Bleeker, 1852). – Long-rectangular, anterior and posterior margins distinctly incised in the middle, pointed posterodorsal and rounded anterodorsal projections. Sulcus centered, slender-to-shoe-sole shape, slightly shifted anteriorly.

Family Eleotridae

Dormitator maculatus (Bloch, 1792). – Long-rectangular, dorsally rounded. Anterior and ventral margins finely crenulated, other margins smooth. Sulcus centered, shoe-sole shape; cauda almost as large as ostium and posteriorly pointed.

Hypseleotris compressa (Krefft, 1864). – High-rectangular. All margins smooth, anterior and posterior margins with sub-median constriction at the level of the sulcus. Sulcus centered, shoe-sole shape, rounded cauda. Ostium with angled dorsal lobe.

Tateurndina ocellicauda Nichols, 1955. – High-rectangular, all margins relatively smooth except for dorsal margin, which is serrate. Sulcus centered, rounded-to-shoe-sole shape, cauda and ostium only weakly differentiated.

Family Milyeringidae

Milyeringa veritas Whitley, 1945. – Trapezoid and smooth with posteroventral projection and dorsally pointing anterodorsal projection. Sulcus centered, rounded-to-shoe-sole shape, surrounded by a crista, cauda and ostium not clearly differentiated.

Family Odontobutidae

Odontobutis obscurus (Temminck and Schlegel, 1845). – Trapezoid, dorsal margin with tip in its posterior half, anterior margin with spine-like dorsal projection, ventral margin crenulated and with spine-like posterior projection, posterior margin deeply sinuate. Sulcus centered, supra-median, relatively small, perciform-like. Cauda slender, posteriorly bent, ostium rounded and distant from anterior rim.

Perccottus glenii Dybowski, 1877. – Trapezoid with sinuate dorsal and ventral margins, posterior margin with median notch, anterior margin with sub-median notch. Sulcus centered, rounded-to-shoe-sole shape, cauda slightly shorter than ostium.

Family Rhyacichthyidae

Protogobius attiti Watson and Pöllabauer, 1998. – Trapezoid/triangular, rounded with short median projection on posterior margin, dorsal margin smooth, ventral margin lobed. Sulcus shifted anteriorly, perciform-like with slender, relatively short cauda and broad ostium; ostium opened to anterior rim.

Rhyacichthys aspro (Valenciennes, 1837). – Trapezoid/triangular with short median projection on posterior and dorsal margins, dorsal margin slightly undulated with prominent

anterodorsal bulge, ventral margin crenate. Sulcus shifted anteriorly, perciform-like with slender, relatively short cauda and broad ostium; ostium anteriorly closed.

Rhyacichthys guilberti Dingerkus and Séret, 1992. – Trapezoid/triangular, ventral margin lobed, dorsal margin smooth. Sulcus shifted anteriorly, perciform-like with slender, relatively short cauda and broad ostium; ostium opened to anterior rim.;

Family Apogonidae (outgroup)

Sphaeramia nematoptera (Bleeker, 1856). – Overall shape longish-ovate, with projections on the shallower dorsal rim. Perciform sulcus slightly above the middle, with broad ostium and slender cauda which are of equal length.

Phylogenetic analyses

Phylogenetic relationships of the extant species

Molecular data. – The Bayesian phylogeny based on molecular data (Fig 4A) recovers all families as monophyletic. Notably, although we used a restricted number of species, the tree is completely congruent with the trees published by Agorreta et al. [12] and Thacker et al. [10]: Rhyacichthyidae and Odontobutidae are sister groups, and together they are sister to the rest of the gobioid families. Milyeringidae, Eleotridae, Butidae, and Thalasseleotrididae are successive sister groups to the 5brG clade, which is composed of well-supported Oxudercidae and Gobiidae.

Maximum Parsimony analysis produced a single most parsimonious tree (Fig S1), which is similar to the Bayesian tree, but some nodes have poor bootstrap support (BS). The tree is topologically identical to the Bayesian tree on family-level, only within Gobiidae there

are some minor differences concerning the positions of *Amblygobius* and *Asterropteryx*; BS for several nodes is rather low compared to the support in the Bayesian tree.

Morphological data. – In the Bayesian phylogeny restricted to the extant species, the 5brG clade (Gobiidae + Oxudercidae) is recovered with maximum support, but the internal structure of the clade is completely unresolved (Fig 4B). The thalasseleotridid species *G. radiatus* and *Th. iota* group together and are sister to 5brG. The Thalasseleotrididae + 5brG clade is maximally supported, consistent with molecular phylogenetic results. Also, similar to the established molecular phylogeny, Eleotridae, Butidae, and Milyeringidae are closely related to that clade (1.00). However, only Eleotridae and Milyeringidae are recovered as monophyletic (0.91, 0.82), whereas Butidae are paraphyletic (Fig 4B). Monophyly of Odontobutidae is also not resolved, and in contrast to the molecular phylogeny the odontobutid species are closer to the above assemblage (1.00) instead of being sister to Rhyacichthyidae, which are recovered paraphyletic (Fig 4B).

The Maximum Parsimony analysis recovered one most parsimonious tree (Fig S2), which is overall similar to the Bayesian tree, but many nodes have very poor bootstrap support (BS). Within the 5brG clade (BS = 95%) the reciprocal monophyly of Gobiidae and Oxudercidae is not recovered. Thalasseleotrididae is monophyletic and highly supported as sister to 5brG (99%). As in the Bayesian tree, among the remaining families Eleotridae (71%) and Milyeringidae (52%) are supported as monophyletic.

Total-evidence approach. – The Bayesian phylogeny inferred from the total-evidence dataset (=combined molecular and morphological data) including only extant species is topologically identical to the molecular phylogeny but shows maximum support for Rhyacichthyidae and Rhyacichthyidae + Odontobutidae (1.00 and 1.00 vs. 0.62 and 0.64, respectively) (Fig S3).

The Maximum Parsimony analysis recovered one most parsimonious tree (Fig S4), which is similar to the Bayesian tree. Most nodes have good bootstrap support, except for some clades within 5brG that are weakly supported. Concerning the topology, the only difference is that the positions of the oxudercid genera *Eucyclogobius*, *Pomatoschistus* and *Chlamydogobius* are resolved while they form a polytomy in the Bayesian tree (see Fig S3 vs. Fig S4).

Fig 4. 50% MRC Bayesian tree with posterior probabilities. A Tree based on published DNA data of the 29 gobioid species used in this study (average standard deviation of split frequencies between two independent runs [ASDSF] = 0.003776; for sources of molecular data see Table 1). **B** Tree based on morphological characters of the extant species only (ASDSF = 0.002446). Scale bar, average number of substitutions per site respectively character changes per character.

Phylogenies including extant and all ten fossil species

Morphological data. – In the Bayesian phylogeny, the 5brG clade (Gobiidae + Oxudercidae) – including the two fossil *Gobius* spp. – is recovered only with moderate support (0.70), and internal relationships are again unresolved (Fig 5A). The rest of the tree is topologically similar to the one based on the morphology of the extant species only (Fig 4B), Thalasseleotrididae are now recovered with slightly higher support (0.80 vs. 0.77). The two †*Eleogobius* spp. are placed between Thalasseleotrididae and 5brG (0.60), but monophyly of the genus is not resolved. The †*Pirskenius* and †*Paralates* spp. form a weakly supported clade (0.64) that is sister to the above assemblage (0.73), but only †*Pirskenius* (i.e., †Pirskeniidae) is resolved as monophyletic (0.87). The remaining two fossil species, †*Lepidocottus aries* and

†*Carlomonnius quasigobius*, are clearly more related to Thalasseleotrididae + 5brG than to Rhyacichthyidae and Odontobutidae (0.84), but their exact placements are not resolved.

The Maximum Parsimony analysis using the same data set recovered six most parsimonious trees. The resulting 50% majority rule consensus tree (Fig S5) is overall consistent with the one based on extant species only (Fig S2) but is very poorly resolved on a deeper level; most nodes have BS < 50%. The two fossil *Gobius* spp. are included in the 5brG in a clade with *Gobius niger*, *Discordipinna* and *Lesueurigobius*. The two †*Eleogobius* spp. are sister to 5brG but monophyly of the genus remains unresolved. †*Carlomonnius quasigobius* is placed in a clade containing Eleotridae and *Kribia nana* (Butidae). †*Pirskenius* is monophyletic (64%) but its position, as well as that of the remaining three fossil species, is not further resolved.

Total-evidence approach. – The Bayesian phylogenetic analysis of the total-evidence dataset including all 29 extant and the ten fossil species produced a consensus tree with a largely collapsed backbone (Fig 5B) compared to the tree based on only the morphological data set of the same taxa (Fig 5A). It contains numerous polytomies and shows poor support for many deeper nodes. The 5brG clade (Gobiidae + Oxudercidae) forms a polytomy with the Thalasseleotrididae, the Butidae, the clade of †*Paralates* + †*Pirskenius* and three further fossil species (†*Eleogobius* brevis, †*E. gaudanti*, †"*Gobius*" *francofurtanus*). Nevertheless, several groups can be recovered within this polytomy. Oxudercidae, which were not resolved in the phylogeny based only on morphology (Fig 5A), are now recovered with high support (0.93), as well as some clades within Gobiidae that correspond to the molecular phylogeny, i.e. the *Aphia* (0.89) and *Glossogobius* (0.72) clades. †*Gobius jarosi* is weakly (0.55) placed as sister to the extant *G. niger*. The clade of †*Pirskenius* + †*Paralates* (see above) is recovered with slightly weaker support (0.59 vs. 0.64), while support for monophyly of †Pirskeniidae is

slightly increased (0.93 vs. 0.87). †*Lepidocottus aries* and †*Carlomonnius quasigobius* are resolved as members of Butidae, albeit with low support (0.61).

The consensus tree of the two most parsimonious trees of the same data set (Fig S6) is even less well resolved, with very weak BS for many nodes. The 5brG clade is recovered, with reciprocally monophyletic Oxudercidae and Gobiidae. In contrast to the Bayesian tree, †"*Gobius*" *francofurtanus* is sister to *Gobius niger* (< 50%) (vs. not resolved in the Bayesian tree), whereas †*Gobius jarosi* is sister to *Lesueurigobius* (also < 50%) (vs. sister to *G. niger*). †*Eleogobius* is monophyletic and sister to the 5brG clade, but again BS for this is negligible. †*Pirskenius* is monophyletic (65%) but its position, as well as that of the remaining four fossil species, is not further resolved.

Fig 5. 50% MRC Bayesian trees with posterior probabilities based on the extant plus ten fossil species. A Tree based on only the morphological data set (ASDSF = 0.006790). **B** Tree based on the total evidence data set (ASDSF = 0.010856). Scale bars, average number of substitutions per site respectively character changes per character.

Total-evidence phylogenies including one to four fossil species

In the following analyses, a single fossil was added to the data set of the extant species and the trees were inferred based on the morphological and molecular data (total-evidence approach).

†*Carlomonnius quasigobius*. – The Bayesian phylogeny is topologically identical to the molecular and the total evidence phylogenies based on extant species only; †*Carlomonnius quasigobius* is placed within Butidae (Fig 6A). Support values are high (>0.90) for most clades, but not for Butidae (0.74 vs. 1.00 in the molecular tree). The single Maximum

Parsimony tree retained recovered all recent families, like the Bayesian tree (Fig S7). However, in this tree †*C. quasigobius* is placed as sister to Thalasseleotrididae + 5brG, albeit with very low support (< 50%).

†*Lepidocottus aries*. – The Bayesian phylogeny (Fig 6B) and the single Maximum Parsimony tree retained (Fig S8) reveal the same topology and almost the same support values as described above for the trees including †*C. quasigobius*. †*Lepidocottus aries* is placed within Butidae in both the Bayesian and the Maximum Parsimony tree, with high support values in the former (0.95), but very low support in the latter (< 50%).

Fig 6. 50% MRC Bayesian trees with posterior probabilities based on the total-evidence dataset. A †*Carlomonnius quasigobius* was added to the extant species (ASDSF = 0.003939). **B** †*Lepidocottus aries* was added to the extant species (ASDSF = 0.000854). Scale bars, average number of substitutions per site respectively character changes per character.

†*Gobius jarosi* and †“*Gobius*” *francofurtanus*. – In the Bayesian tree (Fig 7A), topologies and support values are similar as described above. In comparison to the Bayesian total-evidence phylogeny based on extant taxa only (Fig S3), somewhat decreased support values concern two internal gobiid clades: the *Aphia*-lineage (0.95 vs. 1.00) and the clade containing *Glossogobius* and two members of the *Cryptocentrus*-lineage (0.90 vs. 1.00). The two fossil species are recovered as successive sister groups to *G. niger* with moderate support (0.77) (Fig 5D). In the consensus tree of two Maximum Parsimony trees (Fig S9), †“*Gobius*” *francofurtanus* is sister to *Gobius niger* while the position of †*G. jarosi* is unresolved in a clade with *Lesueurigobius sanzi*, *Tigrigobius multifasciatus*, *Asterropteryx semipunctata*, *Amblygobius phalaena*, and *Aphia minuta* (Fig S9). When only one of the two fossil species is

included, a sister-relation with *G. niger* is apparent in each case, in both the Bayesian and the Maximum Parsimony tree (Figs S10–S13).

†*Eleogobius brevis* and †*E. gaudanti*. – The Bayesian phylogeny inferred from the total-evidence dataset including all extant species and either †*E. brevis* or †*E. gaudanti* (Figs S14, 15) is topologically identical to the molecular phylogeny, and almost no decrease of support values is seen. †*Eleogobius brevis* is recovered as sister to *Gobius niger* with good support (0.87), while †*E. gaudanti* shows a well-supported (0.87) sister relation to the Thalasseleotrididae. When both species of †*Eleogobius* are added, they are recovered in a polytomy with the Thalasseleotrididae (0.88) (Fig 7B), and there is slightly decreased support for the 5brG clade (0.90 vs. 1.00), and also for some of the gobiid clades (e.g. *Tigrigobius* + *G. niger*: 0.91 vs. 1.00) compared to the total evidence tree using only the extant species (Fig S3).

In the Maximum Parsimony trees, when only one of the †*Eleogobius* spp. is included, its position matches that recovered in the Bayesian analyses, albeit with poor support (Figs S16, 17). When both species are included, they form a clade and are sister to the Thalasseleotrididae (< 50%, Fig S18).

Fig 7. 50% MRC Bayesian trees with posterior probabilities based on the total-evidence dataset. A †“*Gobius*” *francofurtanus* and †*G. jarosi* were added to the extant species (ASDSF = 0.014107). **B** Both †*Eleogobius* species were added to the extant species (ASDSF = 0.004114). Scale bars, average number of substitutions per site respectively character changes per character.

†*Pirskenius diatomaceus* and †*P. radoni*. – In the Bayesian tree, topologies are the same as described above; the two species of †*Pirskenius* are recovered as sister to Thalasseleotrididae

(Fig 8A). However, in comparison to the Bayesian total-evidence phylogeny based on extant taxa only, decreased support values occur for the 5brG clade (0.89 vs. 1.00), the Thalasseleotrididae (0.65 vs. 1.00), and for the gobiid clade of *Tigrigobius* + *Gobius niger* (0.90 vs. 1.00). Support for the clade Thalasseleotrididae plus one of the †*Pirskenius* species is similar when only †*P. radoni* is added (0.69) (Fig S19), but higher when only †*P. diatomaceus* is involved (0.91) (Fig S20).

The single Maximum Parsimony tree including both species of †*Pirskenius* recovers all recent families as monophyletic (Fig S21) and the genus is recovered as sister to Thalasseleotrididae + 5brG; its monophyly is supported with 74% BS. Adding solely †*P. radoni* or †*P. diatomaceus* results in the same topology and similar support values (Figs. S22, S23) as seen in the tree including both species.

†*Paralates bleicheri* and †*Pa. chapelcorneri*. – Inclusion of both species of †*Paralates* does not recover a relationship of these two fossil taxa with any of the extant clades in the Bayesian phylogeny, rather they form a polytomy with the Odontobutidae + Rhyacichthyidae clade and the clade containing all other families (Fig 8B). The Bayesian phylogeny that includes only †*Pa. bleicheri* resolves this species as sister to Odontobutidae (Fig S24), albeit with relatively low support (0.60). In comparison with the total-evidence phylogeny including only extant taxa, support for Odontobutidae decreased (0.61 vs. 1.00), while the high support for all other deeper nodes was not affected. The Bayesian phylogeny that contains only †*Pa. chapelcorneri* recovers this species as sister to the clade containing the 5brG, Thalasseleotrididae, Butidae and Eleotridae, but with very low support (0.52) (Fig S25; note also decreased support for some backbone nodes in this tree).

In the Maximum Parsimony analyses, inclusion of both species could not resolve their phylogenetic position, as in the Bayesian tree, but here the resolution of the backbone of the tree is even more severely reduced (Fig S26). The Maximum Parsimony results for †*Pa.*

bleicheri (Fig S27) match those of the Bayesian analyses, whereas when only †*Pa. chapelcorneri* is included, it is placed within Thalasseleotrididae (< 50%, Fig S28).

†*Pirskenius* spp. and †*Paralates* spp. – When all four †*Paralates* and †*Pirskenius* species were included in the Bayesian analysis, they were recovered in a †*Paralates* + †*Pirskenius* clade (0.74), which was resolved as sister to Thalasseleotrididae with moderate support (0.79) (Fig S29). In the Maximum Parsimony tree †*Pirskenius* is recovered monophyletic (64%) but its position and the positions of the two †*Paralates* species within a clade together with Butidae, Thalasseleotrididae and 5brG are not resolved; overall, this tree shows very poor resolution at its backbone (Fig S30).

Fig 8. 50% MRC Bayesian trees with posterior probabilities based on the total-evidence dataset. A †*Pirskenius diatomaceus* and †*P. radoni* were added to the extant species (ASDSF = 0.017673). **B** †*Paralates bleicheri* and †*Pa. chapelcorneri* were added to the extant species (ASDSF = 0.006995). Scale bars, average number of substitutions per site respectively character changes per character.

Discussion

In this study, we have assembled for the first time a dataset comprising molecular and morphological data for Gobioidei that encompasses both extant and fossil species. This approach was necessary as a phylogenetic analysis of the extant species based solely on their morphological characters could only resolve those clades for which morphological apomorphies are known, i.e. 5brG, Thalasseleotrididae, Thalasseleotrididae+5brG, and Eleotridae [see 15, 28], while Butidae, Odontobutidae and Rhyacichthyidae each were recovered as paraphyletic. The overall objective was to investigate whether a fossil gobioid

species can be confidently placed at family level in the tree of the extant Gobioidei using a Bayesian or Maximum Parsimony total-evidence phylogenetic approach. The results reveal mostly well supported placement at family level when a single fossil species is added to the total evidence data set of the extant species, especially in the Bayesian setting.

Five of the fossil species used here had previously been assigned at family level based on a comparative approach: †*Lepidocottus aries* had been placed within Butidae [44], †"*Gobius*" *francofurtanus* and †*Gobius jarosi* had been proposed as members of Gobiidae [46, 100], †*Pirskenius* spp. had been placed in its own family †Pirskeniidae [49] and a sister group relation of †Pirskeniidae to Thalasseleotrididae + 5brG has been suggested [11]. Each of those fossil taxa have been recovered in corresponding positions in the present study (Figs 6B, 7A, 8A). This implies that using comparative morphology has been a very appropriate method to classify those fossils. The family assignment of the remaining five fossil species analyzed here (†*Carlomonnius quasigobius,* †*Eleogobius* spp., †*Paralates* spp.) had been left as *incertae sedis* in previous work because they possess a mosaic set of characters that is not known among extant gobioids [42, 43, 64].

†*Carlomonnius quasigobius* originates from the Eocene of Monte Bolca in northern Italy [64], from the lower to middle Eocene (Ypresian to Lutetian, c. 50–40 Ma, see [101]). It is placed in Butidae in our study (Fig 6A), and it shares with some Butidae (especially with *Kribia*) a very small size, but any comparative approach would not have assigned †*C. quasigobius* to this family because it has only five branchiostegal rays (vs. six in Butidae) and a continuous dorsal fin (vs. divided). The feature that seems to have placed †*C. quasigobius* within the Butidae and close to *Kribia* is the number of 11 branched and segmented caudal fin rays, which is uncommon among other Gobioidei. Nevertheless, given that †*C. quasigobius* is the oldest gobioid species currently known [64], an assignment to the Gobiidae (with which it shares the number of five branchiostegal rays) seems unlikely and its classification within the Butidae appears to be more plausible. It would expand the fossil record of Butidae from the

early Oligocene (c. 30 Ma, [102]) to the early-middle Eocene (c. 50–40 Ma). However, an additional possibility is that †*C. quasigobius* is a member of an extinct gobioid family or a "stem gobioid" showing a mixture of derived characters (five branchiostegal rays, dorsal postcleithrum absent, 11 (7+6) segmented and branched caudal-fin rays, four pelvic-fin rays) and plesiomorphic ones (dorsal fin continuous, 24 (10+14) vertebrae, autogenous haemal spine of the second preural centrum, first two abdominal centra shortened, first dorsal-fin pterygiophore inserting in the second interneural space) [64].

In case of †*Eleogobius* and †*Paralates*, the resulting phylogenies indicate that these genera are not monophyletic and their species may not even belong to the same family (see Figs 7B, 8B, S14–S18, S24–S28). The two species of †*Eleogobius* have been reported from the lower and middle Miocene (c. 17–14 Ma) of Central Europe, specifically southern Germany [42], Austria ([103], as *Gobius*), Switzerland [104, 105], and Croatia [106]. They have been interpreted as belonging to the same genus because they share a T-shaped palatine, absence of an endopterygoid and presence of six branchiostegal rays, and their otoliths are superficially similar, but show differences to recognize the two species [42]. Of those characters, the T-shaped palatine and absence of an endopterygoid can be considered as apomorphic [23, 28], and would support assignment to Gobiidae, which is proposed, based on our phylogenetic results, for †*E. brevis* (Fig 7A). In contrast, the phylogenetic position of †*E. gaudanti* near Thalasseleotrididae (Fig 7B) is difficult to understand as no potential synapomorphies are known that can be recognized in a fossil. However, it is more or less consistent with the hypothesis of Gierl and Reichenbacher [42] that †*Eleogobius* is somewhat "in-between" the 5brG clade and the 6brG gobioids. Furthermore, Bradić-Milinović et al. [41] have recognized a difference in the arrangement of the branchiostegals in the two *Eleogobius* species, which appears to support the possibility that †*Eleogobius* is not monophyletic.

In the case of †*Paralates*, a family assignment had not previously been proposed. †*Paralates bleicheri* has only been found in lower Oligocene deposits of the southern Upper

Rhinegraben [40]. An assignment of †*P. bleicheri* to the Odontobutidae, as indicated in our analysis, receives little support based on its skeletal traits as all shared characters with the Odontobutidae represent plesiomorphic character states (e.g. seven spines in the first dorsal fin, 8–9 rays in the second dorsal fin, presence of postmaxillary process) (see Suppl. Table 1). Finds of fossil skeletons of †*P. bleicheri* with otoliths preserved in situ would be necessary to reinforce this hypothesis.

†*Paralates chapelcorneri* originates from the upper Eocene "Chapelcorner Fish bed" of southern England (Isle of Wight) [38, 43]. No otoliths have yet been reported from the "Chapelcorner Fish bed". The family assignment of this species remains a topic of future research based on new material of this species. Moreover, a possible relationship between †*Paralates* spp. and †*Pirskenius* spp. was indicated in the Bayesian and Maximum Parsimony trees (Figs S25, S26), which is possibly due to their specific combination of plesiomorphic (e.g. seven spines in the first dorsal fin, nine anal-fin rays) and apomorphic traits (e.g. 12 abdominal vertebrae, presence of interneural gap).

Our study also yields some new insights from a methodological point of view. Adding morphological data from only extant species to the molecular dataset had practically no influence on the tree topology and support values (Fig S3). Likewise, the inclusion of a single fossil or of two congeneric species did not change the tree topology, only sometimes some support values decreased (Figs 6–8). However, when all fossils were included in the total evidence phylogenetic framework, the resolution of relationships between families and most fossil taxa dramatically collapsed (Fig 5B). Notably, the morphological phylogeny including the extant and all fossil species was less severe collapsed in the backbone of the tree as the 5brG clade and the Thalasseleotrididae were resolved (Fig 5A). It seems that in the case of the total evidence phylogeny the fossil taxa added a high level of conflicting phylogenetic signals, which could not be overcome by the molecular data despite the latter having orders of magnitude more characters and harbouring strong signal for resolving gobioid phylogeny. A

possible explanation is that the fossil taxa do not only add morphological information, but also a lot of question marks to the matrix, because, depending on their preservation, some morphological traits cannot be determined. An additional (or alternative) explanation is that many extinct gobioid clades and families, each with a unique character combination, existed in the past [11, 41, 45, 49]. These cannot be 'forced' in the tree of extant species and eventually may also be responsible for the collapse of the molecular backbone of the extant families. This highlights that increased sampling of fossil taxa in a total-evidence context is not universally beneficial, as might be expected, but strongly depends on the study group and peculiarities of the morphological data.

Conclusion

We have presented a total-evidence dataset comprising molecular and morphological data of 29 extant gobioid species representing all families. Bayesian and Maximum Parsimony analyses revealed that this dataset is sufficient to achieve a molecular 'backbone' that fully conforms to previous molecular work. The new dataset can be used to analyze the family assignment of fossil skeletal-based gobioid species using Bayesian and Maximum Parsimony total-evidence phylogenetic approaches, which has not been possible before.

Our phylogenetic analyses confirmed the family assignment of those fossil gobioid species for which such a placement had been proposed in previous works. It is thus evident that comparative morphology remains an appropriate method to classify some gobioid fossils. However, our phylogenetic analyses could also suggest relationships of fossil gobioid species for cases where the comparative approach did not yield conclusive results. An example is †*Carlomonnius quasigobius*, which is the oldest gobioid fossil to date and our phylogeny suggests that it could be a possible member of the Butidae, which would expand the known

age of fossil butids from the early Oligocene (30 Ma) to the early-middle Eocene (40–50 Ma). Although such positioning of †*C. quasigobius* remains somewhat speculative for now, it can give hints to look at certain fossil species from a different and new perspective.

We think that the total evidence framework presented here will be beneficial for all future work dealing with the phylogenetic placement of fossil gobioids and thus will help to improve our understanding of the evolutionary history of these fascinating fishes.

Acknowledgements

For providing technical assistance and access to specimens from the SNSB-ZSM collection we thank D. Neumann and U. Schliewen, respectively, (both SNSB-ZSM, Munich, Germany), with special thanks to the latter for help in the acquisition of specimens and insightful discussions. We are grateful to E. Bernard (NHMUK, London, UK), S. Merker (SMNS, Stuttgart, Germany), M. Parrent (MRAC, Tervuren, Belgium), and T. Přikryl (Charles University, Prague, Czech Republic), who all helped to study the specimens kept in the collections of their institutions. Sincere thanks go to W. Schwarzhans (Hamburg, Germany) for providing photographs of the otoliths of *Lesueurigobius sanzi*, *Asterropteryx semipunctata*, *Eucyclogobius newberry, Thalasseleotris iota* and *Dormitator maculatus* (shown in Fig 3) and D. Nolf and K. Hoedemakers (both IRSNB, Brussels, Belgium) for supplying the otolith SEM image of *Odontobutis obscurus* (shown in Fig 3). We thank G. Wörheide (LMU Munich, Germany) for providing access to computational resources. Finally we thank the reviewers and the Academic Editor of PLOS One for their constructive and valuable remarks, which greatly helped to improve the manuscript.

Author Contributions

Conceptualization: Christoph Gierl, Bettina Reichenbacher.

Data curation: Christoph Gierl, Bettina Reichenbacher.

Formal analysis: Martin Dohrmann, Christoph Gierl, Bettina Reichenbacher.

Funding acquisition: Bettina Reichenbacher, Radek Šanda, Philippe Keith.

Investigation: Bettina Reichenbacher, Christoph Gierl.

Methodology: Martin Dohrmann, Christoph Gierl, Bettina Reichenbacher.

Resources: Bettina Reichenbacher, Radek Šanda, Jasna Vukić, Philippe Keith, William Humphreys, Hamid R. Esmaeili.

Supervision: Bettina Reichenbacher.

Validation: Bettina Reichenbacher, Christoph Gierl, Martin Dohrmann.

Visualization: Bettina Reichenbacher, Martin Dohrmann, Christoph Gierl.

Writing – original draft: Christoph Gierl, Martin Dohrmann, Bettina Reichenbacher.

Writing – review & editing: Bettina Reichenbacher, Martin Dohrmann, Christoph Gierl, Philippe Keith, Radek Šanda, Jasna Vukić, Hamid R. Esmaeili, William Humphreys.

References

1. Nelson JS, Grande TC, Wilson MVH. Fishes of the World, Fifth Edition. Hoboken, New Jersey: John Wiley & Sons, inc.; 2016. 752 p.

2. Parenti P. A checklist of the gobioid fishes of the world (Percomorpha: Gobiiformes). Iran J Ichthyol. 2021;8:1–480. doi: 10.22034/iji.v8i0.556.

3. Brandl SJ, Goatley CHR, Bellwood DR, Tornabene L. The hidden half: ecology and evolution of cryptobenthic fishes on coral reefs. Biological Reviews. 2018;93(4):1846–73. Epub May 7. doi: 10.1111/brv.12423. PubMed PMID: WOS:000446427600008.

4. Patzner RA, Van Tassell JL, Kovačić M, Kapoor BG, editors. The biology of gobies. 1 ed. Enfield, New Hampshire: Science Publishers Inc.; 2011.

5. Tornabene L, Robertson DR, Baldwin CC. *Varicus lacerta*, a new species of goby (Teleostei, Gobiidae, Gobiosomatini, *Nes* subgroup) from a mesophotic reef in the southern Caribbean. ZooKeys. 2016;596:143–56. doi: 10.3897/zookeys.596.8217. PubMed PMID: WOS:000378055700010; PubMed Central PMCID: PMC4926659.

6. Jaafar Z, Murdy EO, editors. Fishes Out of Water: Biology and Ecology of Mudskippers. 1st ed. Boca Raton: Taylor & Francis; 2017.

7. Keith P. Biology and ecology of amphidromous Gobiidae of the Indo-Pacific and the Caribbean regions. J Fish Biol. 2003;63(4):831–47. Epub Sep 26. doi: 10.1046/j.1095-8649.2003.00197.x. PubMed PMID: WOS:000186047000001.

8. Karplus I, Szlep R, M T. Goby-shrimp partner specificity. I. Distribution in the northern Red Sea and partner specificity. J Exp Mar Biol Ecol. 1981;51(1):1–19. Epub Mar 31, 2003. doi: 10.1016/0022-0981(81)90151-9.

9. Karplus I, Thompson AR. The partnership between gobiid fishes and burrowing alpheid shrimps. In: Patzner RA, Van Tassell JL, Kovačić M, Kapoor BG, editors. The biology of gobies. Enfield, NH: Science Publishers Inc.; 2011. p. 559–607.

10. Thacker CE, Satoh TP, Katayama E, Harrington RC, Eytan RI, Near TJ. Molecular phylogeny of Percomorpha resolves *Trichonotus* as the sister lineage to Gobioidei (Teleostei: Gobiiformes) and confirms the polyphyly of Trachinoidei. Molecular phylogenetics and evolution. 2015;93:172–9. Epub Aug. doi: 10.1016/j.ympev.2015.08.001. PubMed PMID: MEDLINE:26265255; PubMed Central PMCID: PMC26265255.

11. Reichenbacher B, Přikryl T, Cerwenka AF, Keith P, Gierl C, Dohrmann M. Freshwater gobies 30 million years ago: New insights into character evolution and phylogenetic relationships of †Pirskeniidae (Gobioidei, Teleostei). PloS one. 2020;15(8):e0237366. Epub Aug 24. doi: 10.1371/journal.pone.0237366. PubMed PMID: WOS:000565550400035.

12. Agorreta A, San Mauro D, Schliewen U, Van Tassell JL, Kovačić M, Zardoya R, et al. Molecular phylogenetics of Gobioidei and phylogenetic placement of European gobies. Molecular phylogenetics and evolution. 2013;69(3):619–33. doi: 10.1016/j.ympev.2013.07.017. PubMed PMID: MEDLINE:23911892.

13. McCraney WT, Thacker CE, Alfaro ME. Supermatrix phylogeny resolves goby lineages and reveals unstable root of Gobiaria. Molecular phylogenetics and evolution. 2020;151:106862. Epub May 28. doi: 10.1016/j.ympev.2020.106862.

14. Akihito, Iwata A, Kobayashi T, Ikeo K, Imanishi T, Ono H, et al. Evolutionary aspects of gobioid fishes based upon a phylogenetic analysis of mitochondrial cytochrome *b* genes. Gene. 2000;259(1–2):5–15. doi: 10.1016/S0378-1119(00)00488-1. PubMed PMID: ISI:000166338300003.

15. Gill AC, Mooi RD. Thalasseleotrididae, new family of marine gobioid fishes from New Zealand and temperate Australia, with a revised definition of its sister taxon, the Gobiidae (Teleostei: Acanthomorpha). Zootaxa. 2012;3266(1):41–52. doi: 10.11646/zootaxa.3266.1.3.

16. Thacker CE. Biogeography of goby lineages (Gobiiformes: Gobioidei): origin, invasions and extinction throughout the Cenozoic. J Biogeogr. 2015;42(9):1615–25. Epub Jun 13. doi: 10.1111/jbi.12545. PubMed PMID: WOS:000359376600004.

17. McAllister DE. Evolution of the branchiostegals and classification of teleostome fishes. Bull natn Mus Can (Biol Ser). 1968;77:1–239.

18. Carpenter KE, Niem VH. The living marine resources of the Western Central Pacific. Volume 5. Bony fishes part 3 (Menidae to Pomacentridae). Rome: Food and Agriculture Organization of the United Nations (FAO); 2001. 2791–3380 p.

19. Fraser TH. Comparative osteology of the shallow water cardinal fishes (Perciformes: Apogonidae) with reference to the systematics and evolution of the family. Ichthyological Bulletin of the J L B Smith Institute of Ichthyology. 1972;34:1–105.

20. Regan CT. The osteology and classification of the gobioid fishes. The Annals and Magazine of Natural History [Eighth Series]. 1911;8(48):729–33.

21. Van Tassell JL, Tornabene L, Taylor MS. A history of gobioid morphological systematics. In: Patzner RA, Van Tassell JL, Kovačić M, Kapoor BG, editors. The biology of gobies. Enfield, NH: Science Publishers Inc.; 2011. p. 3–22.

22. Akihito. A systematic examination of the gobiid fishes based on the Mesopterygoid, Postcleithra, Branchiostegals, pelvic fins, Scapula, and Suborbital. Jpn J Ichthyol. 1969;16(3):93–104. Epub Jun 28, 2010. doi: 10.11369/jji1950.16.93. PubMed PMID: BCI:BCI197051058231.

23. Hoese DF. Gobioidei: relationships. In: Moser HG, Richards WJ, Cohen DM, Fahay MP, Kendall J, A. W., Richardson SL, editors. Ontogeny and systematics of fishes. Gainesville, Florida: American Society of Ichthyologists and Herpetologists; 1984. p. 588–91.

24. Miller PJ. The osteology and adaptive features of *Rhyacichthys aspro* (Teleostei: Gobioidei) and the classification of gobioid fishes. Journal of Zoology. 1973;171(3):397–434. doi: 10.1111/j.1469-7998.1973.tb05347.x.

25. Thacker CE, Hardman MA. Molecular phylogeny of basal gobioid fishes: Rhyacichthyidae, Odontobutidae, Xenisthmidae, Eleotridae (Teleostei: Perciformes: Gobioidei). Molecular phylogenetics and evolution. 2005;37(3):858–71. Epub 2005/06/25. doi: 10.1016/j.ympev.2005.05.004. PubMed PMID: 15975831.

26. Harrison IJ. Specialization of the gobioid palatopterygoquadrate complex and its relevance to gobioid systematics. Journal of Natural History. 1989;23(2):325–53. doi: 10.1080/00222938900770211.

27. Pezold F. Evidence for a monophyletic Gobiinae. Copeia. 1993;1993(3):634–43. doi: 10.2307/1447224. PubMed PMID: BCI:BCI199396109145.

28. Hoese DF, Gill AC. Phylogenetic relationships of eleotridid fishes (Perciformes, Gobioidei). B Mar Sci. 1993;52(1):415–40. PubMed PMID: ISI:A1993KX23000015.

29. Thacker CE. Phylogeny of Gobioidei and placement within Acanthomorpha, with a new classification and investigation of diversification and character evolution. Copeia. 2009;2009(1):93–104. doi: 10.1643/Ci-08-004. PubMed PMID: ISI:000263748000013.

30. Larson HK. A revision of the gobiid fish genus *Mugilogobius* (Teleostei: Gobioidei), and its systematic placement. Rec West Aust Mus. 2001;62:1–233. doi: 10.18195/issn.0313-122x.62.2001.001-233.

31. Murdy EO. A taxonomic revision and cladistic analysis of the oxudercine gobies (Gobiidae: Oxudercinae). Rec West Aust Mus Suppl. 1989;11:1–93. Epub Jun 16, 2009. doi: 10.3853/j.0812-7387.11.1989.93. PubMed PMID: BCI:BCI199089035472.

32. Harold AS, Winterbottom R, Munday PL, Chapman RW. Phylogenetic relationships of Indo-Pacific coral gobies of the genus *Gobiodon* (Teleostei: Gobiidae), based on morphological and molecular data. B Mar Sci. 2008;82(1):119–36. PubMed PMID: WOS:000252842600008.

33. Lord C, Bellec L, Dettaï A, Bonillo C, Keith P. Does your lip stick? Evolutionary aspects of the mouth morphology of the Indo-Pacific clinging goby of the *Sicyopterus* genus (Teleostei: Gobioidei: Sicydiinae) based on mitogenome phylogeny. Journal of Zoological Systematics and Evolutionary Research. 2019;57(4):910–25. Epub May 30. doi: 10.1111/jzs.12291.

34. Pezold F. Phylogenetic analysis of the genus *Gobionellus* (Teleostei: Gobiidae). Copeia. 2004;2004(2):260–80. doi: 10.1643/CI-02-218R3. PubMed PMID: WOS:000221336700006.

35. Tornabene L, Deis B, Erdmann MV. Evaluating the phylogenetic position of the goby genus *Kelloggella* (Teleostei: Gobiidae), with notes on osteology of the genus and description of a new species from Niue in the South Central Pacific Ocean. Zool J Linn Soc. 2018;183(1):143–62. Epub Dec 16, 2017. doi: 10.1093/zoolinnean/zlx060. PubMed PMID: WOS:000432303500006.

36. Tornabene L, Greenfield DW, Erdmann MV. A review of the *Eviota zebrina* complex, with descriptions of four new species (Teleostei, Gobiidae). ZooKeys. 2021;1057:149–84. doi: 10.3897/zookeys.1057.66675.

37. Gaudant J. Sur les conditions de gisement de l'ichthyofaune oligocène d'Aix-en-Provence (Bouches-du-Rhône): Essai de définition d'un modèle paléoécologique et paléogéographique. Géobios. 1978;11(3):393–7. doi: 10.1016/S0016-6995(78)80039-4.

38. Gaudant J, Quayle WJ. New palaeontological studies on the Chapelcorner fish bed (Upper Eocene, Isle of Wight). Bull Br Mus nat Hist (Geol). 1988;44(1):15–39.

39. Gaudant J. Nouvelles observations sur les poissons oligocènes de Monteviale (Vicenza - Italie). Mem Sci Geol. 1978;32:1–9.

40. Gaudant J. Sur la présence de Gobiidae (Poissons téléostéens) dans l'Oligocène inférieur de Rouffach (Haut-Rhin). Sci Géol Bull. 1979;32(3):131–7. doi: 10.3406/sgeol.1979.1560.

41. Bradić-Milinović K, Ahnelt H, Rundić L, Schwarzhans W. The lost freshwater goby fish fauna (Teleostei, Gobiidae) from the early Miocene of Klinci (Serbia). Swiss Journal of Palaeontology. 2019;138(2):285–315. Epub June 1. doi: 10.1007/s13358-019-00194-4.

42. Gierl C, Reichenbacher B. A new fossil genus of Gobiiformes from the Miocene characterized by a mosaic set of characters. Copeia. 2015;103(4):792–805. Epub Nov 12. doi: 10.1643/ci-14-146. PubMed PMID: WOS:000366679700006.

43. Gierl C, Reichenbacher B. Revision of so-called *Pomatoschistus* (Gobiiformes, Teleostei) from the late Eocene and early Oligocene. Palaeontologia Electronica. 2017;20.2.33A:1–17. doi: 10.26879/721. PubMed PMID: WOS:000405188500014.

44. Gierl C, Reichenbacher B, Gaudant J, Erpenbeck D, Pharisat A. An extraordinary gobioid fish fossil from southern France. PloS one. 2013;8(5):e64117. Epub May 15. doi: 10.1371/journal.pone.0064117. PubMed PMID: 23691158; PubMed Central PMCID: PMCPMC3655028.

45. Reichenbacher B, Bannikov AF. Diversity of gobioid fishes in the late middle Miocene of northern Moldova, Eastern Paratethys – part I: an extinct clade of *Lesueurigobius* look-alikes. PalZ. 2022;96:67–112. Epub Aug 26, 2021. doi: 10.1007/s12542-021-00573-8.

46. Reichenbacher B, Gregorová R, Holcová K, Šanda R, Vukić J, Přikryl T. Discovery of the oldest *Gobius* (Teleostei, Gobiiformes) from a marine ecosystem of Early Miocene age. Journal of Systematic Palaeontology. 2018;16(6):493–513. Epub May 2, 2017. doi: 10.1080/14772019.2017.1313323. PubMed PMID: WOS:000424810000002.

47. Schwarzhans W, Ahnelt H, Carnevale G, Japundžić S, Bradić K, Bratishko A. Otoliths in situ from Sarmatian (Middle Miocene) fishes of the Paratethys. Part III: tales from the cradle of the Ponto-Caspian gobies. Swiss Journal of Palaeontology. 2017;136(1):45–92. Epub Nov 10, 2016. doi: 10.1007/s13358-016-0120-7.

48. Gaudant J. Mise en évidence des plus anciens Gobioidei (Poissons téléostéens) connus dans le Lutétien inférieur marin de Catalogne (Espagne). Comptes rendus de l'Académie des sciences Série II, Sciences de la terre et des planètes. 1996;322(1):71–6. PubMed PMID: ISI:A1996TT86800009.

49. Obrhelová N. Vergleichende Osteologie der tertiären Süsswasserfische Böhmens (Gobioidei). Sbornik Paläont. 1961;26:103–92.

50. Schwarzhans W, Brzobohatý R, Radwańska U. Goby otoliths from the Badenian (middle Miocene) of the Central Paratethys from the Czech Republic, Slovakia and Poland: A baseline for the evolution of the European Gobiidae (Gobiiformes; Teleostei). Bollettino della Società Paleontologica Italiana. 2020;59(2):125–73. doi: 10.4435/bspi.2020.10. PubMed PMID: WOS:000562503100004.

51. Schwarzhans W, Agiadi K, Carnevale G. Late Miocene-Early Pliocene evolution of Mediterranean gobies and their environmental and biogeographic significance. Riv It Paleont Strat. 2020;126(3):657–724. PubMed PMID: WOS:000584561400003.

52. Keith P. Threatened fishes of the world: *Rhyacichthys guilberti* Dingerkus & Séret, 1992 (Rhyacichthyidae). Environ Biol Fish. 2002;63(1):40–. doi: 10.1023/a:1013827820971. PubMed PMID: WOS:000173466700006.

53. Lord C, Keith P. Threatened fishes of the world: *Protogobius attiti* Watson & Pöllabauer, 1998 (Rhyacichthyidae). Environ Biol Fish. 2006;77(1):101–2. doi: 10.1007/s10641-006-9060-1. PubMed PMID: WOS:000239741100010.

54. Akihito. Some morphological characters considered to be important in gobiid phylogeny. In: Matsuura K, editor. Indo-Pacific Fish Biology: Proceedings of the Second International Conference on Indo-Pacific Fishes. Tokyo: Ichthyological Society of Japan; 1986. p. 629–39.

55. Iwata A, Jeon S-R, Mizuno N, Choi K-C. A revision of the eleotrid goby genus *Odontobutis* in Japan, Korea and China. Jpn J Ichthyol. 1985;31(4):373–88. Epub Feb 23, 2011. doi: 10.11369/jji1950.31.373. PubMed PMID: WOS:A1985ADJ0200005.

56. Sparks JS, Chakrabarty P. Revision of the endemic Malagasy Cavefish genus *Typhleotris* (Teleostei: Gobiiformes: Milyeringidae) with discussion of its phylogenetic placement and description of a new species. American Museum Novitates. 2012;3764:1–28. doi: 10.1206/3764.2. PubMed PMID: WOS:000312236300001.

57. Hoese DF, Larson HK. New Australian fishes. Part 11. A new genus and species of eleotridid (Gobioidei) from Southern Australia with a discussion of relationships. Memoirs of the Museum of Victoria. 1987;48(1):43–50. PubMed PMID: BCI:BCI198987014125.

58. Schwarzhans W. Reconstruction of the Fossil Marine Bony Fish Fauna (Teleostei) from the Eocene to Pleistocene of New Zealand by Means of Otoliths: With Studies of Recent Congroid, Morid and Trachinoid Otoliths. Milano: Museo Civico di Storia Naturale di Milano; 2019. 390 p.

59. Hoese DF, Roberts CD. A new species of the eleotrid genus *Thalasseleotris* (Teleostei: Gobioidei) from New Zealand coastal waters. J Roy Soc New Zeal. 2005;35(4):417–31. doi: 10.1080/03014223.2005.9517793. PubMed PMID: ISI:000234492300004.

60. Birdsong RS, Murdy EO, Pezold FL. A study of the vertebral column and median fin osteology in gobioid fishes with comments on gobioid relationships. B Mar Sci. 1988;42(2):174–214. PubMed PMID: ISI:A1988N503000003.

61. Kindermann G, Miljković N, Ahnelt H, Stevenson DE. The osteology of *Eucyclogobius newberryi* and *Quietula guaymasiae* (Teleostei: Gobiidae), two closely related Gobionellines from the East Pacific. Annalen des Naturhistorischen Museums in Wien Serie B Botanik und Zoologie. 2007;108:13–56. PubMed PMID: BCI:BCI200700465922.

62. Gierl C. Articulated gobioid skeletons from the Frankfurt-Formation (Lower Miocene). München: Ludwig-Maximilians-Universität München; 2012.

63. Near TJ, Dornburg A, Eytan RI, Keck BP, Smith WL, Kuhn KL, et al. Phylogeny and tempo of diversification in the superradiation of spiny-rayed fishes. Proc Natl Acad Sci U S A. 2013;110(31):12738–43. doi: 10.1073/pnas.1304661110. PubMed PMID: 23858462; PubMed Central PMCID: PMC3732986.

64. Bannikov AF, Carnevale G. †*Carlomonnius quasigobius* gen. et sp. nov.: the first gobioid fish from the Eocene of Monte Bolca, Italy. Bulletin of Geosciences. 2016;91(1):13–22. Epub Dec 2, 2015. doi: 10.3140/bull.geosci.1577. PubMed PMID: WOS:000372546600002.

65. Gaudant J. Présence du genre *Lepidocottus* Sauvage, 1875 (Teleostei, Gobioidei) dans l'Oligocène inférieur des environs de Céreste (Alpes-de-Haute-Provence, France). Geodiversitas. 2015;37(2):229–35. doi: 10.5252/g2015n2a4. PubMed PMID: WOS:000357401300004.

66. Reichenbacher B, Gaudant J. On *Prolebias meyeri* (Agassiz) (Teleostei, Cyprinodontiformes) from the Oligo-Miocene of the Upper Rhinegraben area, with the establishment of a new genus and a new species. Eclogae Geologicae Helvetiae. 2003;96(3):509–20. doi: 10.1007/s00015-003-1098-x. PubMed PMID: WOS:000189178600014.

67. Přikryl T. A new species of the sleeper goby (Gobioidei, Eleotridae) from the České Středohoří Mountains (Czech Republic, Oligocene) and analysis of the validity of the family Pirskeniidae. Paläontol Z. 2014;88(2):187–96. Epub Jun 28, 2013. doi: 10.1007/s12542-013-0188-y.

68. La Mesa M, Arneri E, Caputo V, Iglesias M. The transparent goby, *Aphia minuta*: review of biology and fisheries of a paedomorphic European fish. Rev Fish Biol Fisheries. 2005;15(1–2):89–109. doi: 10.1007/s11160-005-1613-4. PubMed PMID: WOS:000233725400006.

69. Rojo AL. Osteología del chanquete, *Aphya minuta* (Risso, 1810) (Pisces: Gobiidae). Bol Inst Esp Oceanogr. 1985;2(1):165–79. PubMed PMID: BCI:BCI198681089950.

70. Van Tassell JL, Miller PJ, Brito A. A revision of *Vanneaugobius* (Teleostei: Gobiidae), with description of a new species. Journal of Natural History. 1988;22(2):545–67. Epub Feb 17, 2007. doi: 10.1080/00222938800770371.

71. Hoese DF, Fourmanoir P. *Discordipinna griessingeri*, a new genus and species of gobiid fish from tropical Indo-West Pacific. Jpn J Ichthyol. 1978;25(1):19–24. doi: 10.11369/jji1950.25.19. PubMed PMID: ISI:A1978FG43000003.

72. Esmaeili HR, Baghbani S, Zareian H, Shahryari F. Scale morphology of tank goby *Glossogobius giuris* (Hamilton-Buchanan, 1822) (Perciformes: Gobiidae) using scanning electron microscope. Journal of Biological Sciences. 2009;9(8):899–903. doi: 10.3923/jbs.2009.899.903.

73. McKay SI, Miller PJ. The affinities of European sand gobies (Teleostei: Gobiidae). Journal of Natural History. 1997;31(10):1457–82. doi: 10.1080/00222939700770791. PubMed PMID: ISI:A1997XZ44500001.

74. Miller PJ. Gobiidae. In: Whitehead PJP, Bauchot M-L, Hureau J-C, Nielsen J, Tortonese E, editors. Fishes of the north-eastern Atlantic and the Mediterranean (FNAM). III. Paris: UNESCO; 1986. p. 1019–85.

75. Randall JE, Hoese DF. Revision of the Indo-Pacific dartfishes, genus *Ptereleotris* (Perciformes: Gobioidei). Indo-Pacific Fishes. 1985;7:1–36.

76. Miller PJ. Affinities, origin and adaptive features of the Australian Desert Goby *Chlamydogobius eremius* (Zietz, 1896) (Teleostei: Gobiidae). Journal of Natural History. 1987;21(3):687–705. Epub Feb 17, 2007. doi: 10.1080/00222938700770391. PubMed PMID: WOS:A1987H259500009.

77. Mestermann K, Zander CD. Vergleichende osteologische Untersuchungen an *Pomatoschistus*-Arten (Gobioidei, Pisces). Zool Jb Anat. 1984;111:501–42.

78. Whitley GP. New fishes from Australia and New Zealand. Proceedings of the Royal Zoological Society of New South Wales. 1956;for the Year 1954–55:34–8.

79. McDowall RM. Descriptive and taxonomic notes on *Grahamichthys radiatus* (Valenciennes), Eleotridae. Trans Roy Soc NZ, Zool. 1965;7(2):51–6.

80. Schwarzhans W. Reconstruction of the fossil marine bony fish fauna (Teleostei) from the Eocene to Pleistocene of New Zealand by means of otoliths. Memorie della Società Italiana di Scienze Naturali e del Museo di Storia Naturale di Milano. 2019;46:1–326.

81. Wongrat P. Systematics, comparative anatomy, and phylogeny of eleotrine gobies (Teleostei: Gobioidei): University of Bristol; 1977.

82. Larson HK, Foster R, Humphreys WF, Stevens MI. A new species of the blind cave gudgeon *Milyeringa* (Pisces: Gobioidei, Eleotridae) from Barrow Island, Western Australia, with a redescription of *M. veritas* Whitley. Zootaxa. 2013;3616(2):135–50. doi: 10.11646/zootaxa.3616.2.3. PubMed PMID: WOS:000315021500003; PubMed Central PMCID: PMC24758799.

83. Iwata A, Sakai H. *Odontobutis hikimius* n. sp.: A new freshwater goby from Japan, with a key to species of the genus. Copeia. 2002;2002(1):104–10. doi: 10.1643/0045-8511(2002)002[0104:ohnsan]2.0.co;2. PubMed PMID: BCI:BCI200200185878.

84. Shibukawa K, Iwata A, Viravong S. *Terateleotris*, a new gobioid fish genus from the Laos (Teleostei, Perciformes), with comments on its relationships. Bulletin of the National Science Museum Series A (Zoology). 2001;27(4):229–57. PubMed PMID: BCI:BCI200200292381.

85. Bergman LMR. The cephalic lateralis system of cardinalfishes (Perciformes: Apogonidae) and its application to the taxonomy and systematics of the family: University of Hawaii at Manoa; 2004.

86. McAllister DE. Mandibular pore pattern in the sculpin family Cottidae. Nat Mus Can Bull. 1968;223:58–69. PubMed PMID: BCI:BCI19684900097393.

87. Maddison WP, Maddison DR. Mesquite: a modular system for evolutionary analysis. Version 3.61 https://www.mesquiteproject.org. 3.61 ed2019.

88. Larsson A. AliView: a fast and lightweight alignment viewer and editor for large datasets. Bioinformatics. 2014;30(22):3276–8. Epub Aug 5. doi: 10.1093/bioinformatics/btu531. PubMed PMID: WOS:000344774600022; PubMed Central PMCID: PMCPMC4221126.

89. Edgar RC. MUSCLE: multiple sequence alignment with high accuracy and high throughput. Nucleic Acids Research. 2004;32(5):1792–7. doi: 10.1093/nar/gkh340. PubMed PMID: WOS:000220487200025; PubMed Central PMCID: PMCPMC390337.

90. Gouy M, Guindon S, Gascuel O. SeaView version 4: A multiplatform graphical user interface for sequence alignment and phylogenetic tree building. Molecular biology and evolution. 2010;27(2):221–4. doi: 10.1093/molbev/msp259. PubMed PMID: WOS:000273704400003.

91. Ronquist F, Teslenko M, van der Mark P, Ayres DL, Darling A, Höhna S, et al. MrBayes 3.2: efficient Bayesian phylogenetic inference and model choice across a large model space. Systematic Biology. 2012;61(3):539–42. doi: 10.1093/sysbio/sys029. PubMed PMID: WOS:000303336200013; PubMed Central PMCID: PMC3329765.

92. Goloboff PA, Farris JS, Nixon KC. TNT, a free program for phylogenetic analysis. Cladistics. 2008;24(5):774–86. doi: 10.1111/J.1096-0031.2008.00217.X. PubMed PMID: WOS:000259611200009.

93. Goloboff PA, Catalano SA. TNT version 1.5, including a full implementation of phylogenetic morphometrics. Cladistics. 2016;32(3):221–38. Epub Apr 25. doi: 10.1111/cla.12160. PubMed PMID: WOS:000376269000001.

94. Lewis PO. A likelihood approach to estimating phylogeny from discrete morphological character data. Systematic Biology. 2001;50(6):913–25. doi: 10.1080/106351501753462876.

95. Yang Z. Maximum likelihood phylogenetic estimation from DNA sequences with variable rates over sites: Approximate methods. Journal of Molecular Evolution. 1994;39(3):306–14. Epub Sep 1. doi: 10.1007/bf00160154. PubMed PMID: 7932792.

96. Lanave C, Preparata G, Saccone C, Serio G. A new method for calculating evolutionary substitution rates. Journal of Molecular Evolution. 1984;20(1):86–93. doi: 10.1007/bf02101990. PubMed PMID: WOS:A1984SN04000011.

97. Rambaut A, Drummond AJ, Xie D, Baele G, Suchard MA. Posterior summarization in Bayesian phylogenetics using Tracer 1.7. Systematic Biology. 2018;67(5):901–4. Epub 2018/05/03. doi: 10.1093/sysbio/syy032. PubMed PMID: WOS:000443580600012; PubMed Central PMCID: PMCPMC6101584.

98. Felsenstein J. Confidence Limits on Phylogenies: An Approach Using the Bootstrap. Evolution. 1985;39(4):783–91. doi: 10.2307/2408678. PubMed PMID: WOS:A1985APJ8100007.

99. Rambaut A. FigTree. Tree Figure Drawing Tool version 1.4.4. http://tree.bio.ed.ac.uk/software/figtree/. 1.4.4 ed. Edinburgh: University of Edinburgh; 2018.

100. Weiler W. Die Fischfauna des Tertiärs im oberrheinischen Graben, des Mainzer Beckens, des unteren Maintals und der Wetterau, unter besonderer Berücksichtigung des Untermiozäns. Abhandlungen der Senckenbergischen Naturforschenden Gesellschaft. 1963;504:1–75.

101. Marramà G, Bannikov AF, Tyler JC, Zorzin R, Carnevale G. Controlled excavations in the Pesciara and Monte Postale sites provide new insights about the palaeoecology and taphonomy of the fish assemblages of the Eocene Bolca Konservat-Lagerstätte, Italy. Palaeogeography, Palaeoclimatology, Palaeoecology. 2016;454:228-45.

102. Pandolfi L, Carnevale G, Costeur L, Del Favero L, Fornasiero M, Ghezzo E, et al. Reassessing the earliest Oligocene vertebrate assemblage of Monteviale (Vicenza, Italy). Journal of Systematic Palaeontology. 2017;15(2):83–127. Epub Mar 16, 2016. doi: 10.1080/14772019.2016.1147170.

103. Brzobohatý R, Gaudant J. *Gobius brevis* (Agassiz, 1839), a gobiid fish with otoliths in situ (Pisces, Teleostei) in the Karpatian (Lower Miocene) of the Vienna Basin. Annalen des Naturhistorischen Museums in Wien - Serie A (Mineralogie und Petrographie, Geologie und Paläontologie, Archäozoologie, Anthropologie und Prähistorie). 2009;111:245–55. PubMed PMID: BCI:BCI200900349722.

104. Jost J, Kälin D, Börner S, Vasilyan D, Lawver D, Reichenbacher B. Vertebrate microfossils from the Upper Freshwater Molasse in the Swiss Molasse Basin: Implications for the evolution of the North Alpine Foreland Basin during the Miocene Climate Optimum. Palaeogeography, Palaeoclimatology, Palaeoecology. 2015;426:22–33. doi: 10.1016/j.palaeo.2015.02.028. PubMed PMID: WOS:000353603000003.

105. Jost J, Kälin D, Schulz-Mirbach T, Reichenbacher B. Late Early Miocene lake deposits near Mauensee, central Switzerland: fish fauna (otoliths, teeth), accompanying biota and palaeoecology. Eclogae Geologicae Helvetiae. 2006;99(3):309–26. Epub Jan 12, 2007. doi: 10.1007/s00015-006-1198-5.

106. Mandic O, Hajek-Tadesse V, Bakrač K, Reichenbacher B, Grizelj A, Miknić M. Multiproxy reconstruction of the middle Miocene Požega palaeolake in the Southern Pannonian Basin (NE Croatia) prior to the Badenian transgression of the Central Paratethys Sea. Palaeogeography, Palaeoclimatology, Palaeoecology. 2019;516:203–19. Epub Dec 7, 2018. doi: 10.1016/j.palaeo.2018.12.003. PubMed PMID: WOS:000456903000018.

Appendix 1

Short description of the 10 fossil specimens included in this study.

Additional Information

All data matrices and trees are available on figshare (https://figshare.com/s/ed10b9a5ac382f856a20).

Supporting information

Suppl. Table. Part A. Distribution of character states of the characters 1–54 among the examined 29 extant and 10 fossil gobioid species. **Part B.** List of characters, their states and literatur sources. Colour indicates how the character state was determined; ? indicates that character state is not known.

Captions for Figures S1–S30

Fig S1 Maximum Parsimony tree with bootstrap values based on published DNA data of the 29 gobioid species used in this study (single tree retained; for sources of molecular data see Table 1). TL = 10112, CI = 0.407, RI = 0.377.

Fig S2 Maximum Parsimony tree with bootstrap values based on morphological data of the extant species only (single tree retained). TL = 179, CI = 0.559, RI = 0.782.

Fig S3 50% MRC Bayesian tree with posterior probabilities based on a total evidence approach (morphology and DNA) based on the extant species only (ASDSF = 0.000521).

Fig S4 Maximum Parsimony tree with bootstrap values based on a total evidence approach (morphology and DNA) of the extant species only (single tree retained). TL = 10312, CI = 0.409, RI = 0.390.

Fig S5 Maximum Parsimony tree with bootstrap values based on morphological data (50% majority rule consensus of six trees) of the extant species plus ten fossil species (indicated with red frames). TL = 227, CI = 0.467, RI = 0.715.

Fig S6 Maximum Parsimony tree with bootstrap values based on a total evidence approach (morphology and DNA) of the extant species plus all ten fossil species included in this study (50% majority rule consensus of two trees). TL = 10633, CI = 0.397, RI = 0.362.

Fig S7 Maximum Parsimony tree with bootstrap values based on a total evidence approach (morphology and DNA) of the extant species plus †*Carlomonnius quasigobius* (single tree retained). TL = 10321, CI = 0.409, RI = 0.390.

Fig S8 Maximum Parsimony tree with bootstrap values based on a total evidence approach (morphology and DNA) of the extant species plus †*Lepidocottus aries* (single tree retained). TL = 10316, CI = 0.409, RI = 0.390.

Fig S9 Maximum Parsimony tree with bootstrap values based on a total evidence approach (morphology and DNA) of the extant species plus †“*Gobius*” *francofurtanus* and †*Gobius jarosi* (50% majority rule consensus tree of two trees). TL = 10623, CI = 0.397, RI = 0.359.

Fig S10 50% MRC Bayesian tree with posterior probabilities based on a total evidence approach (morphology and DNA) using the extant species and adding †*Gobius francofurtanus* (ASDSF = 0.005129).

Fig S11 50% MRC Bayesian tree with posterior probabilities based on a total evidence approach (morphology and DNA) using the extant species and adding †*Gobius jarosi* (ASDSF = 0.002010).

Fig S12 Maximum Parsimony tree with bootstrap values based on a total evidence approach (morphology and DNA) of the extant species plus †*Gobius jarosi* (single tree retained). TL = 10313, CI = 0.409, RI = 0.390.

Fig S13 Maximum Parsimony tree with bootstrap values based on a total evidence approach (morphology and DNA) of the extant species plus †*Gobius francofurtanus* (single tree retained). TL = 10312, CI = 0.409, RI = 0.390.

Fig S14 50% MRC Bayesian tree with posterior probabilities based on a total evidence approach (morphology and DNA) using the extant species and †*Eleogobius brevis* (ASDSF = 0.001331).

Fig S15 50% MRC Bayesian tree with posterior probabilities based on a total evidence approach (morphology and DNA) using the extant species and †*Eleogobius gaudanti* (ASDSF = 0.000569).

Fig S16 Maximum Parsimony tree with bootstrap values based on a total evidence approach (morphology and DNA) of the extant species plus †*Eleogobius brevis* (single tree retained). TL = 10314, CI = 0.409, RI = 0.390.

Fig S17 Maximum Parsimony tree with bootstrap values based on a total evidence approach (morphology and DNA) of the extant species plus †*Eleogobius gaudanti* (single tree retained). TL = 10315, CI = 0.409, RI = 0.390.

Fig S18 Maximum Parsimony tree with bootstrap values based on a total evidence approach (morphology and DNA) of the extant species plus both †*Eleogobius* species (single tree retained). TL = 10317, CI = 0.409, RI = 0.390.

Fig S19 50% MRC Bayesian tree with posterior probabilities based on a total evidence approach (morphology and DNA) using the extant species and †*Pirskenius radoni* (ASDSF = 0.006008).

Fig S20 50% MRC Bayesian tree with posterior probabilities based on a total evidence approach (morphology and DNA) using the extant species and †*Pirskenius diatomaceus* (ASDSF = 0.003438).

Fig S21 Maximum Parsimony tree with bootstrap values based on a total evidence approach (morphology and DNA) of the extant species plus both †*Pirskenius* species (single tree retained). TL = 10324, CI = 0.409, RI = 0.390.

Fig S22 Maximum Parsimony tree with bootstrap values based on a total evidence approach (morphology and DNA) of the extant species plus †*Pirskenius radoni* (single tree retained). TL = 10320, CI = 0.409, RI = 0.390.

Fig S23 Maximum Parsimony tree with bootstrap values based on a total evidence approach (morphology and DNA) of the extant species plus †*Pirskenius diatomaceus* (single tree retained). TL = 10318, CI = 0.409, RI = 0.390.

Fig S24 50% MRC Bayesian tree with posterior probabilities based on a total evidence approach (morphology and DNA) using the extant species and adding †*Paralates bleicheri* (ASDSF = 0.002460).

Fig S25 50% MRC Bayesian tree with posterior probabilities based on a total evidence approach (morphology and DNA) using the extant species and adding †*Paralates chapelcorneri* (ASDSF = 0.008340).

Fig S26 Maximum Parsimony tree with bootstrap values based on a total evidence approach (morphology and DNA) of the extant species plus both †*Paralates* species (50% majority rule consensus of two trees). TL = 10824, CI = 0.390, RI = 0.339.

Fig S27 Maximum Parsimony tree with bootstrap values based on a total evidence approach (morphology and DNA) of the extant species plus †*Paralates bleicheri* (single tree retained). TL = 10313, CI = 0.409, RI = 0.390.

Fig S28 Maximum Parsimony tree with bootstrap values based on a total evidence approach (morphology and DNA) of the extant species plus †*Paralates chapelcorneri* (single tree retained). TL = 10316, CI = 0.409, RI = 0.390.

Fig S29 50% MRC Bayesian tree with posterior probabilities based on a total evidence approach (morphology and DNA) using the extant species and adding the two species of †*Paralates* and the two species of †*Pirskenius* (ASDSF = 0.008077). Scale bars, average number of substitutions per site.

Fig S30 Maximum Parsimony tree with bootstrap values based on a total evidence approach (morphology and DNA) of the extant species plus the species of †*Pirskenius* and †*Paralates* (50% majority rule consensus of seven trees). TL = 10333, CI = 0.408, RI = 0.389.

Figure1

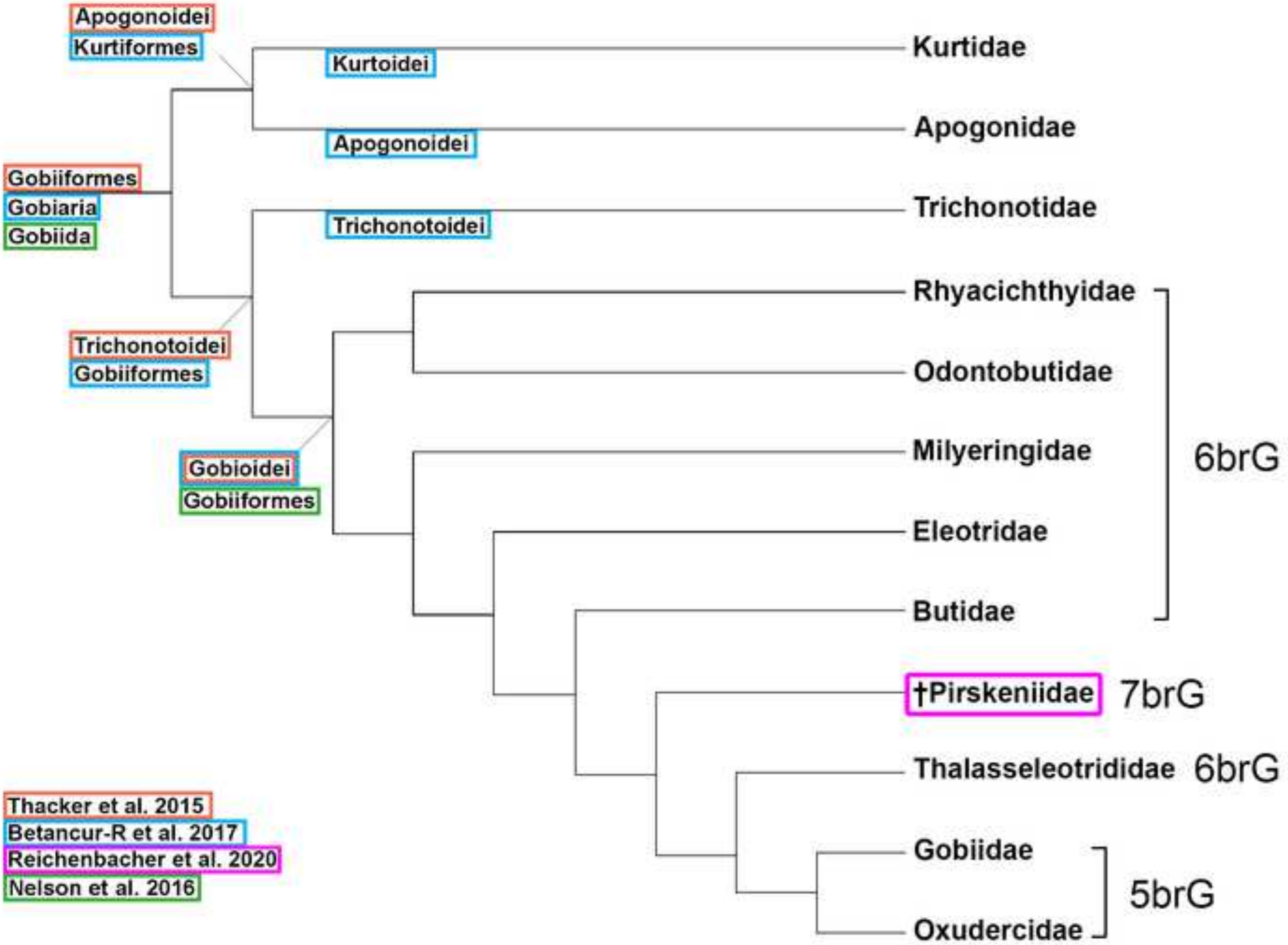

Apogonoidei
Kurtiformes
Kurtoidei
Kurtidae
Apogonidae
Apogonoidei
Gobiiformes
Gobiaria
Gobiida
Trichonotidae
Trichonotoidei
Rhyacichthyidae
Trichonotoidei
Gobiiformes
Odontobutidae
Milyeringidae
6brG
Gobioidei
Gobiiformes
Eleotridae
Butidae
†Pirskeniidae
7brG
Thalasseleotrididae
6brG
Gobiidae
5brG
Oxudercidae
Thacker et al. 2015
Betancur-R et al. 2017
Reichenbacher et al. 2020
Nelson et al. 2016

Figure2

Click here to access/download;Figure;Fig2 revised_LZW.tif

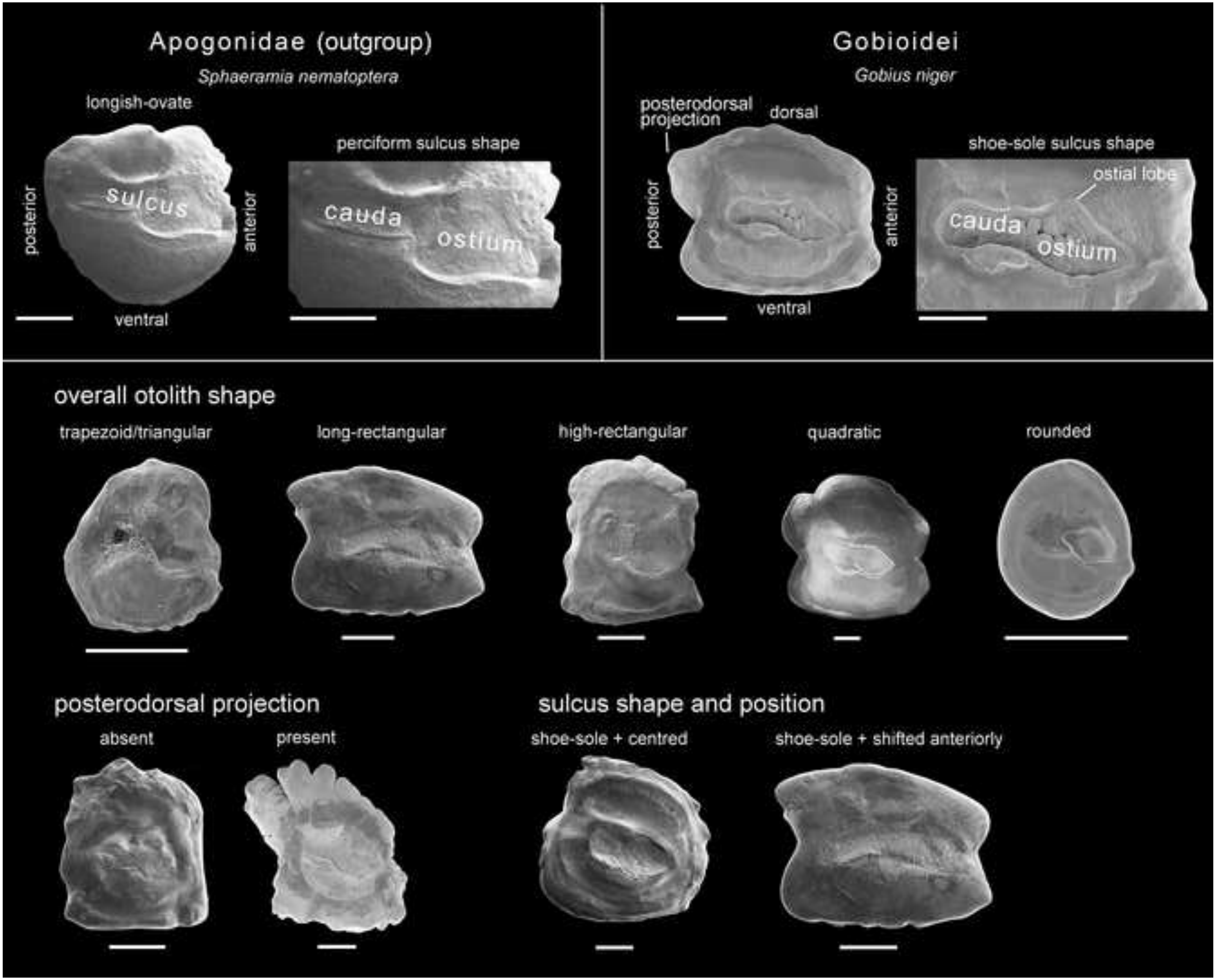

Figure3

Click here to access/download;Figure;Fig3 revised_LZW.tif

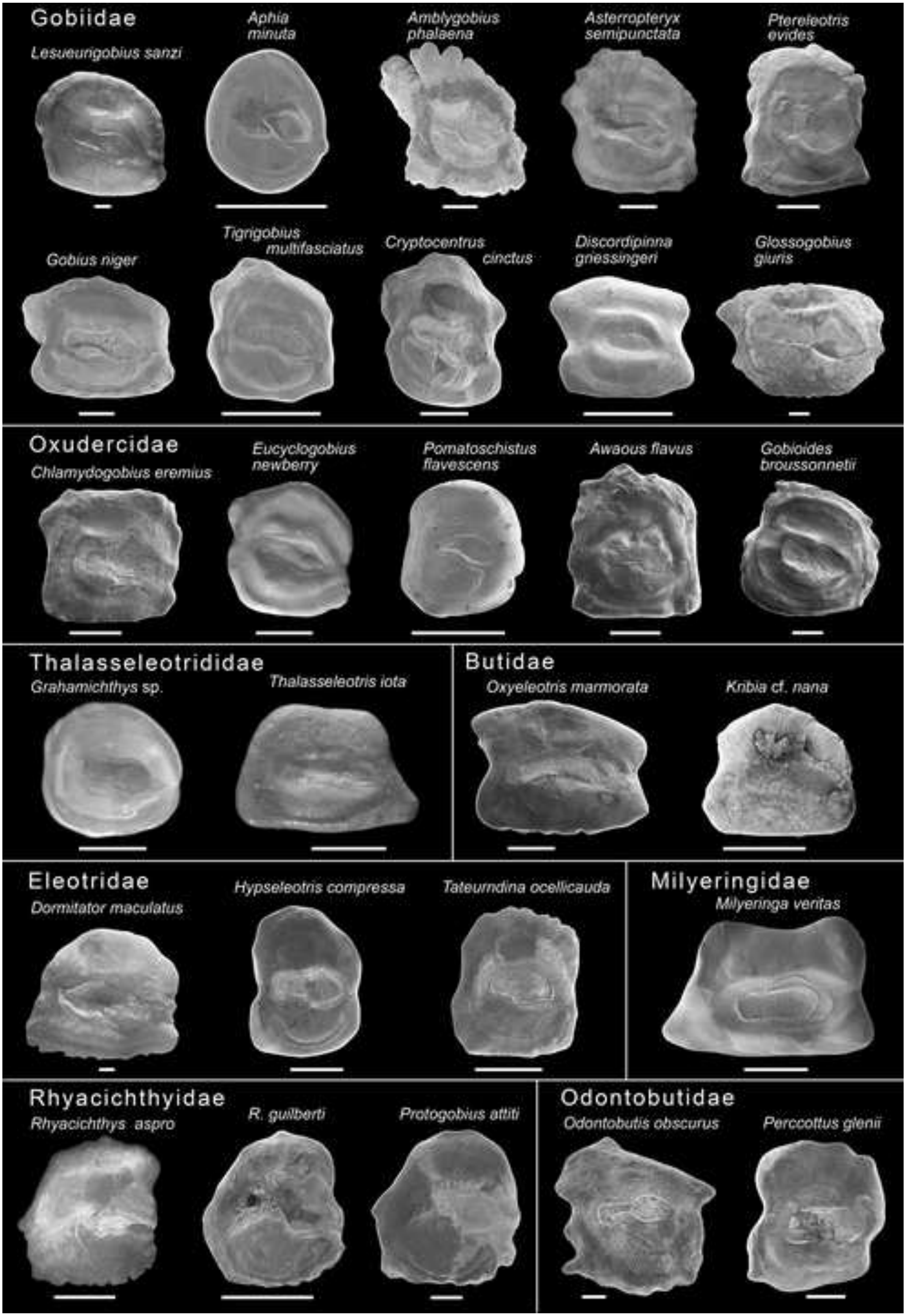

Figure4
Click here to access/download;Figure;Fig4-revised_LZW.tif

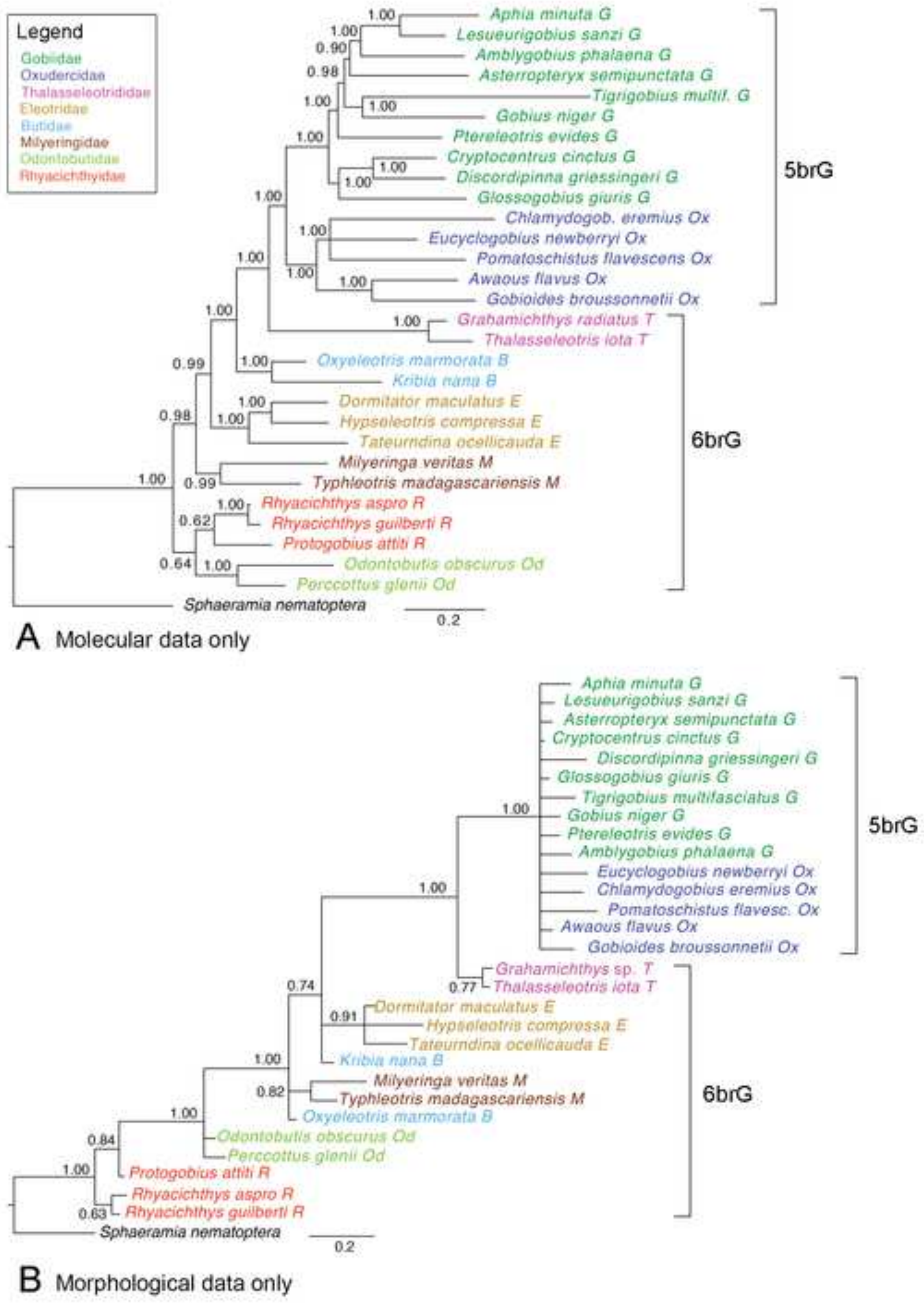

Figure5

Click here to access/download;Figure;Fig5-revised_LZW.tif

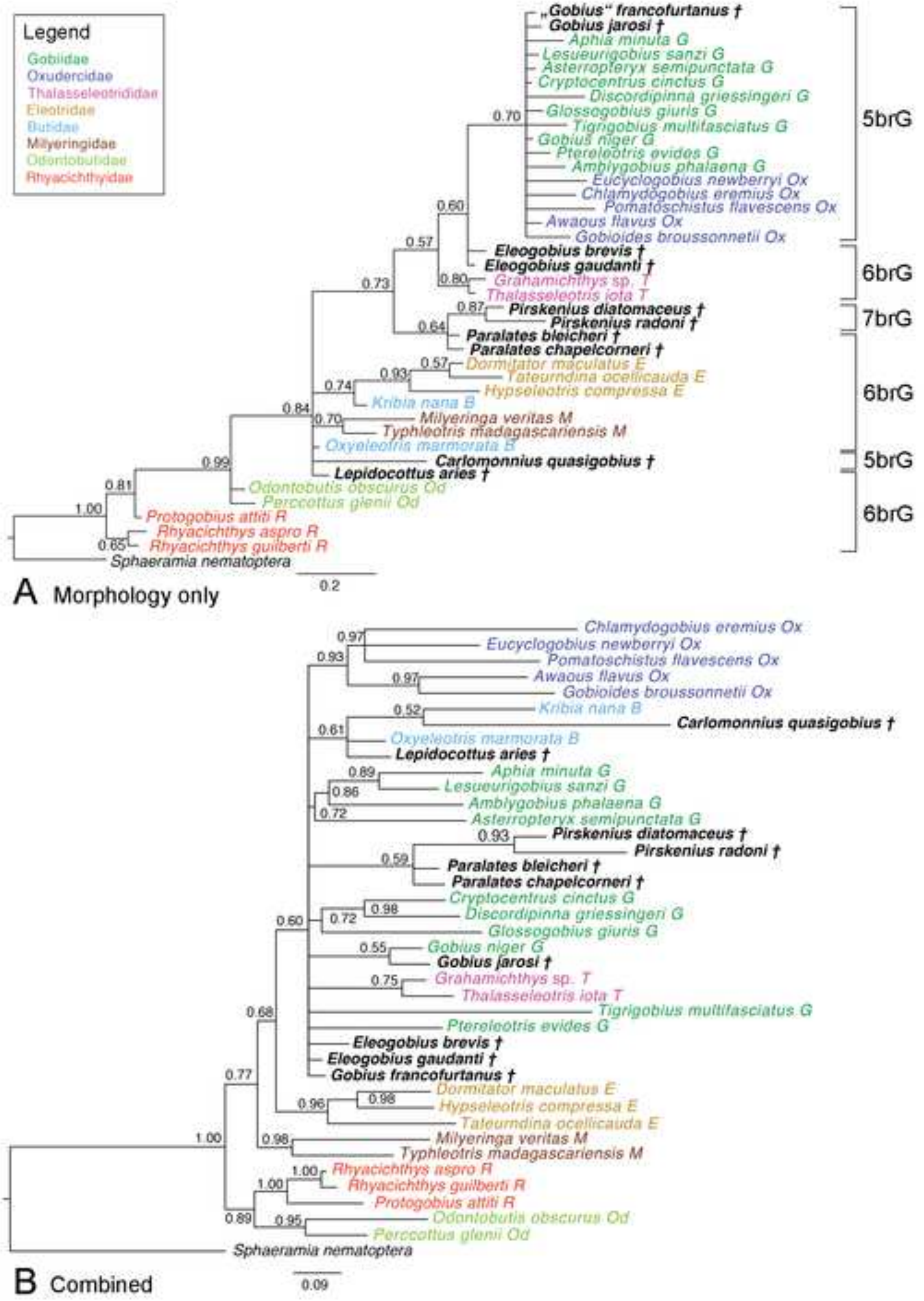

Figure6
Click here to access/download;Figure;Fig6-revised_LZW.tif

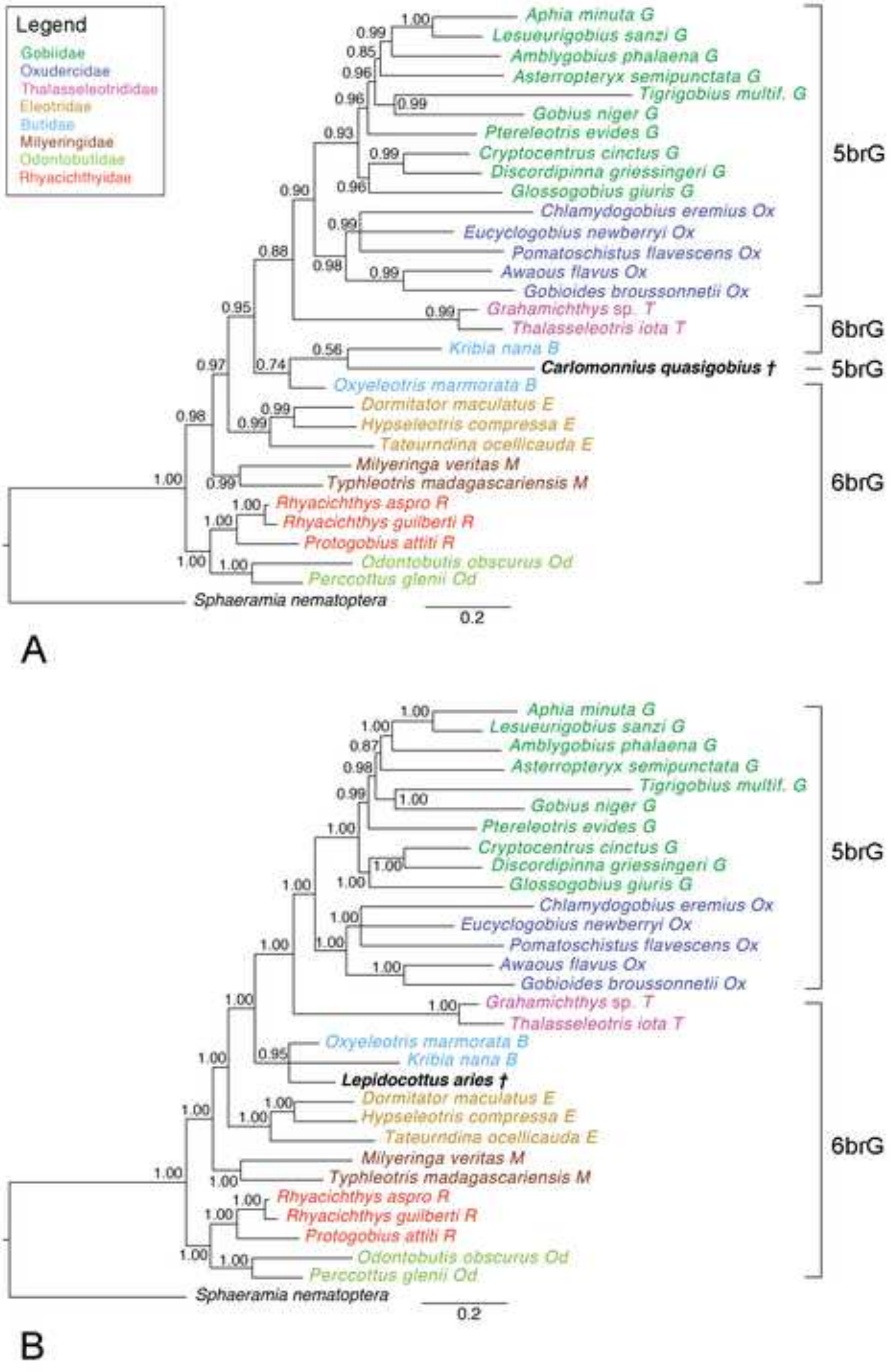

Figure7 Click here to access/download;Figure;Fig7-revised_LZW.tif

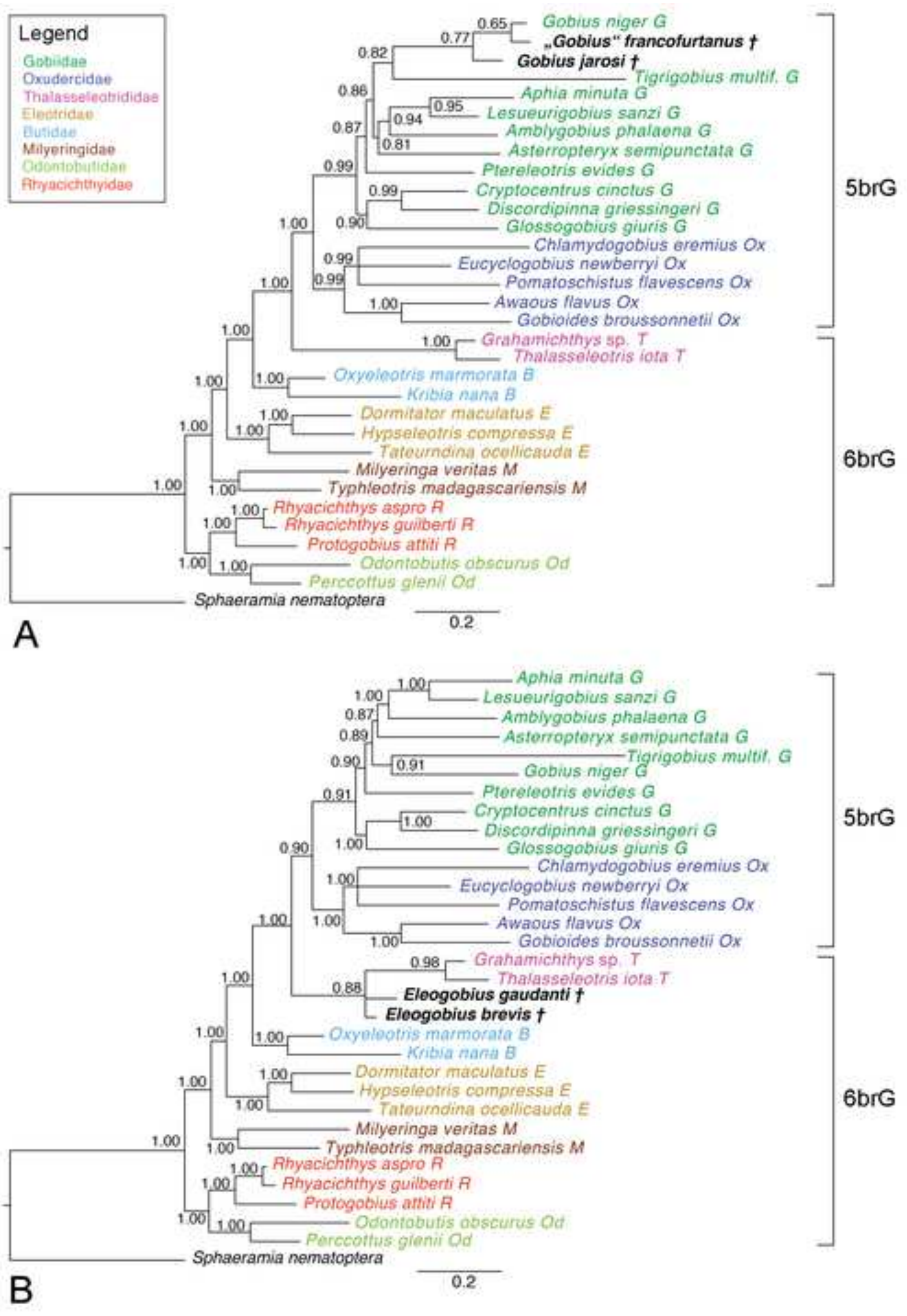

Figure8

Click here to access/download;Figure;Fig8-revised_LZW.tif

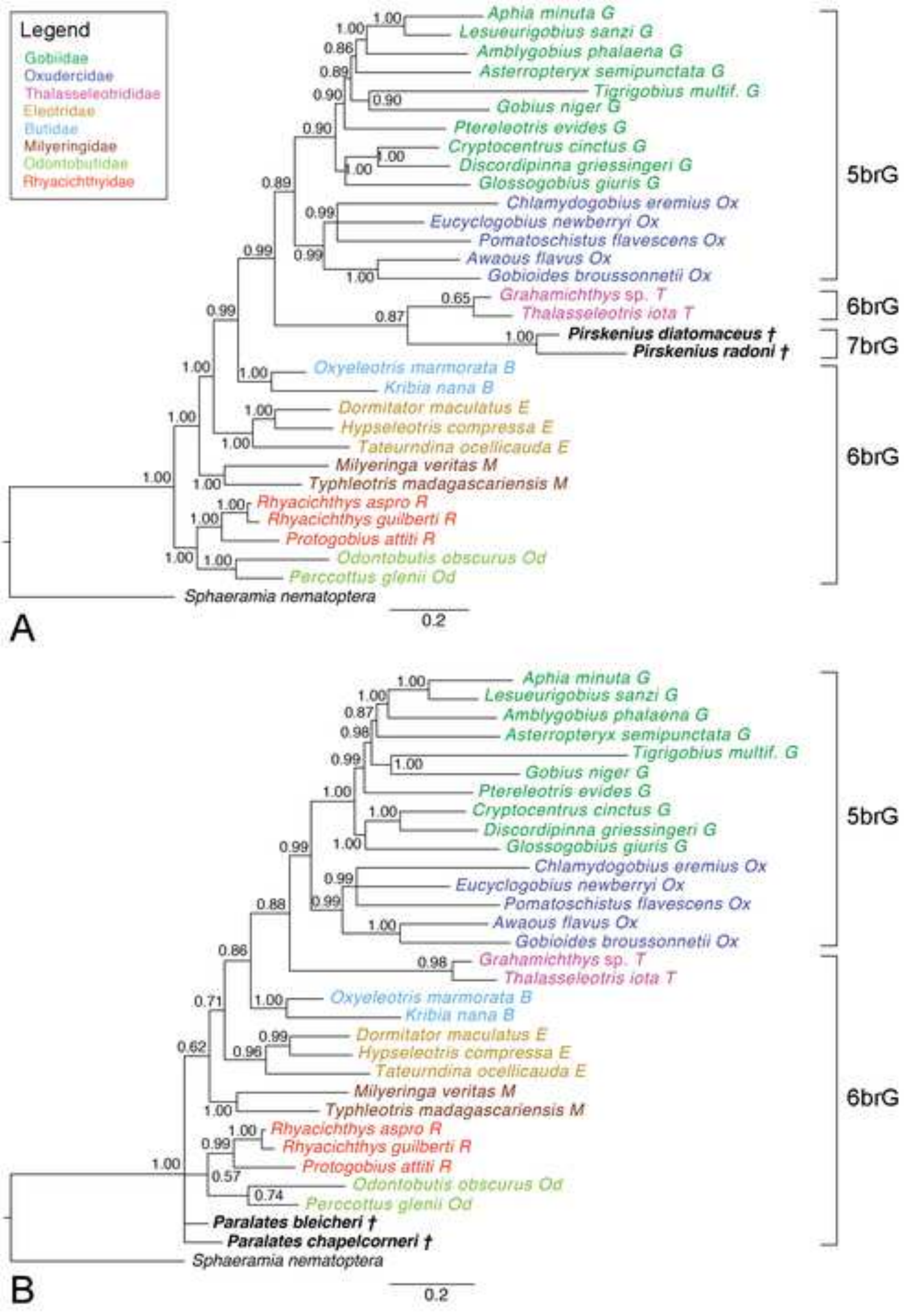

Appendix 1

Click here to access/download
Supporting Information
Gierl et al Appendix 1-revised.docx

Supplementary Table

Click here to access/download
Supporting Information
Gierl et al Suppl Table.xlsx

Supplementary Figures 1-30

Click here to access/download
Supporting Information
renamed_4e981.docx

An integrative phylogenetic approach for inferring relationships of fossil gobioids (Teleostei: Gobiiformes)

Christoph Gierl[1], Martin Dohrmann[1], Philippe Keith[2], William Humphreys[3], Hamid R. Esmaeili[4], Jasna Vukić[5], Radek Šanda[6], Bettina Reichenbacher[1,7]*

[1]Department of Earth and Environmental Sciences, Palaeontology & Geobiology, Ludwig-Maximilians-Universität München, Munich, Germany

[2]UMR 8067 Biologie des organismes et écosystèmes aquatiques (BOREA), Muséum national d'Histoire naturelle, CNRS, IRD, SU, Paris, France

[3]School of Biological Sciences, University of Western Australia, Crawley, WA 6009, Australia; Western Australian Museum, 49 Kew Street, Welshpool, WA 6106, Australia

[4]Ichthyology and Molecular Systematics Research Laboratory, Zoology Section, Department of Biology, College of Sciences, Shiraz University, Shiraz, Iran

[5]Department of Ecology, Charles University, Prague, Czech Republic

[6]Department of Zoology, National Museum, Prague, Czech Republic

[7]GeoBio-Center LMU, Ludwig-Maximilians-Universität München, Munich, Germany

Corresponding author: Bettina Reichenbacher. E-mail: b.reichenbacher@lrz.uni-muenchen.de

Abstract

The suborder Gobioidei is among the most diverse groups of vertebrates, comprising about 2310 species. In the fossil record gobioids date back to the early Eocene (c. 50 m.y. ago), and a considerable increase in numbers of described species is evident since the middle Miocene (c. 16 m.y. ago). About 40 skeleton-based gobioid species and > 100 otolith-based species have been described until to date. However, assignment of a fossil gobioid species to specific families has often remained tentative, even if well preserved complete specimens are available. The reasons are that synapomorphies that can be recognized in a fossil skeleton are rare (or absent) and that no phylogenetic framework applicable to fossils exists. Here we aim to overcome this problem by developing a phylogenetic total evidence framework that is suitable to place a fossil skeleton-based gobioid species at family level. Using both literature and newly collected data we assembled a morphological character matrix (~~54~~ 48 characters) for 29 extant species, representing all extant gobioid families, and ten fossil gobioid species, and we compiled a multi-gene concatenated alignment (supermatrix; 6271 bp) of published molecular sequence data for the extant species. Bayesian and Maximum Parsimony analyses revealed that our selection of extant species was sufficient to achieve a molecular 'backbone' that fully conforms to previous molecular work. Inclusion of a single fossil species in the total evidence data set of the extant species provided new insight in its possible ~~resulted in mostly well supported~~ placement~~s~~ at family level, especially in a Bayesian framework. Five out of the ten fossil species were recovered in the same family as had been suggested in previous works based on comparative morphology. The remaining five fossil species had hitherto been left as family *incertae sedis*. Now, based on our phylogenetic framework, new and mostly well supported ~~and could now be placed for the first time~~ hypotheses to which clades they could belong can be presented~~at family level, albeit not always with high support values~~. Additionally, the extinct genera †*Eleogobius* and †*Paralates* were recovered as paraphyletic

and should be revised in future works. ~~Notably, inclusion of all fossil species simultaneously produced very poorly resolved trees, even for some extant taxa, despite the very strong signal for gobioid relationships contained in the molecular data.~~ We conclude that the total evidence framework presented here will be beneficial for all future work dealing with the phylogenetic placement of a fossil skeleton-based gobioid and thus will help to improve our understanding of the evolutionary history of these fascinating fishes.

Introduction

The suborder Gobioidei (Percomorpha: Gobiiformes) is among the most diverse groups of fishes, encompassing about 320 genera and about 2310 species [1, 2]. Gobioids live in marine, brackish, and freshwater habitats, and form an important component of reef faunas [3-5]. They show a wide range of specializations, including the ability to survive on land for long periods of time (mudskippers; *e.g.* [6]), being diadromous and alternating freshwater and sea water during their lifecycle [7], and forming symbioses with certain shrimp species [8, 9].

Gobioidei belongs to Gobiiformes (*sensu* Thacker et al. [10]), together with Kurtidae, Apogonidae, and Trichonotidae, and consists of nine families: Rhyacichthyidae, Odontobutidae, Milyeringidae, Eleotridae, Butidae, Thalasseleotrididae, Gobiidae, and Oxudercidae (= formerly Gobionellidae [1]), and the extinct †Pirskeniidae [11] (Fig 1). Phylogenetic relationships within extant Gobioidei are generally well resolved based on molecular data [10, 12, 13]. Morphologically, the extant Gobioidei can be divided into two major groups based on the number of branchiostegal rays (bones supporting the gill membrane [14, 15]). Possession of five branchiostegal rays is considered to be the derived state within Gobioidei, supporting the clade Gobiidae + Oxudercidae (denoted 5brG here; see Fig 1), which is also recovered on the basis of molecular data [10, 12, 13]. In contrast, Gobioidei with six branchiostegal rays (6brG) form a paraphyletic group in molecular

phylogenies [12, 16], indicating that this condition is plesiomorphic within Gobioidei. The only gobioid that has seven branchiostegal rays, of which the last ray is expanded as is typical for Gobioidei (see [17]), is the genus †*Pirskenius* (denoted 7brG here), and phylogenetic analyses placed it within the paraphyletic 6brG [11]; see Fig 1. The closest living relatives of Gobioidei, the Apogonidae, Kurtidae, and Trichonotidae, all have seven branchiostegals and no expansion of the last branchiostegal ray [1, 17-19].

Fig 1. Current understanding of gobioid relationships showing the different nomenclatures in use. ~~Note that Gobiiformes/Gobiaria is referred to as Gobiida in Nelson et al. [1], but the Trichonotidae are not included in their Gobiida. Therefore, the Gobiiformes *sensu* [1] corresponds to the Gobioidei *of* Thacker et al. [10] and Betancur-R et al. [20].~~ brG = branchiostegal-rayed Gobioidei.

Formatted: Font: Italic

Previous gobioid classifications date back to more than 100 years ago. Regan [20] had already recognized that two main groups within Gobioidei, which he termed Eleotridae and Gobiidae, can be distinguished based on the configuration of the pelvic fins (separate in Eleotridae vs. united in Gobiidae), the shape of the palatine (L- vs. T-shaped), the endopterygoid (present vs. mostly absent), and the composition of the shoulder girdle (presence vs. absence of the dorsal coracoid [=scapula]). Regan's Eleotridae conforms to the paraphyletic 6brG, and his Gobiidae are reflected in the 5brG. Since then, several efforts have been made to classify groups within Gobioidei using morphological characters (see [21] for a comprehensive compilation). Akihito [22] and Hoese [23] noted that the loss of the anteriormost branchiostegal ray appears to be characteristic for the most advanced groups in Gobioidei (= 5brG). Miller [24] erected the family Rhyacichthyidae and reclassified Gobioidei into Rhyacichthyidae and Gobiidae, with the latter comprising seven subfamilies, Eleotrinae and Xenisthminae (now Eleotridae), Gobionellinae and Tridentigerinae (now

included in Oxudercidae), Gobiinae and Kraemeriinae (now Gobiidae), and †Pirskeniinae (referring to †Pirskeniidae Obrhelová, 1961) [10, 12, 25]. Harrison [26] tried to resolve gobioid interrelationships with the help of features of the palato-pterygoquadrate complex and indicated some putative relationships among six taxa of the 5brG. Pezold [27] recognized a distinct head pore configuration as an autapomorphy of the subfamily Gobiinae (now in Gobiidae). Hoese and Gill [28] recognized the family Odontobutidae and used 16 characters to propose interrelationships among Rhyacichthyidae, Odontobutidae, and Gobiidae; their Gobiidae included the Butinae, Eleotridinae and Gobiinae, each of which is now assigned family rank [12, 29]. Other authors used morphological data for phylogenetic studies of subgroups of various families, as Larson [30] has done for the *Mugilogobius* group of the Gobiidae, and Murdy [31] for the Oxudercinae. Most of the more recent works that includes morphological characters haves focused on smaller groups within Gobioidei (e.g. [29, 32-36]).

The known fossil record of Gobioidei is relatively modest in comparison to their recent diversity and comprises about 40 species that are known from skeletal material. In older studies, these species have been assigned to genera or families by comparing meristic characters, such as counts of vertebrae and fin rays (e.g. [37-40]). More recent authors have also considered the systematic value of certain osteological features (e.g. shape of the palatine, first dorsal fin-pterygiophore formula, number of branchiostegal rays, configuration of the palatine-ectopterygoid complex, presence of the entopterygoid), which enabled a tentative systematic placement of some fossil gobioids in relation to extant taxa [41-47].

However, determining the systematic context of fossil gobioids remains a challenge. Firstly, fossil gobioids apparently included several extinct lineages (e.g. [42, 43, 45, 48-51]). Secondly, a phylogenetic analysis of extant gobioids based on morphological characters is hampered by the rarity of synapomorphic characters [28], and becomes even more difficult in fossil gobioids, which usually preserve only skeletal traits and otoliths [11]. Thirdly, all

comparative approaches suffer from a limited knowledge of the range of skeletal characters of extant gobioids, which is also due to the sheer number of species (currently 2310, see above) and the inaccessibility of some rare taxa [52, 53]. This explains why, with the exception of †Pirskeniidae [11], no phylogenetic analyses have yet been conducted to position fossil gobioids within the phylogeny of extant taxa.

The objective of this study was to place ten selected fossil gobioid species within an integrative ("total evidence") phylogenetic framework including published molecular sequence data from extant species in combination with morphological data for both fossil and extant species. The choice of fossils was largely based on our previous works (see section Material and Methods). The overall idea was that placement of fossil gobioids in the context of a rigorous phylogenetic analysis would greatly help to improve our understanding of the evolutionary history of these fascinating fishes.

Institutional abbreviations

BMNH, former acronym for British Museum of Natural History (now NHMUK); FMNH, Field Museum of Natural History, Chicago, Illinois, USA; IRSNB, Royal Institute of Natural Sciences, Brussel, Belgium; LACM, Los Angeles County Museum of Natural History, Los Angeles, California, USA; MNHN, Muséum national d’Histoire naturelle, Paris, France; MRAC, Musée royal de l’Afrique centrale, Tervuren, Belgium; NHMUK, Natural History Museum in London, United Kingdom; NMP, National Museum, Prague, Czech Republic; SMNS, State Museum for Natural History, Stuttgart, Germany; WAM, Western Australian Museum, Welshpool, Australia; ZM-CBSU, Zoological Museum Collection of the Biology Department at Shiraz University, Iran; ZSM, Bavarian State Collection of Zoology, Munich, Germany.

Material and Methods

Ethic statement

All specimens originate from museum collections (see Table 1) and no specimens were sacrificed for this work. ~~The sampling complied with protocols approved by the responsible governmental authorities (according to the countries where the sampling was conducted). All necessary permits were obtained for the described study, which complied with all relevant regulations. Collection of *Rhyacichthys guilberti* (MNHN 2019-0113) was granted by the Vanuatu Environment Unit (Permit Numbers ENV326/001/1/07/DK and ENV326/001/1/08/D). Collection of *Protogobius attiti* (MNHN 2019-0112) was permitted by the New Caledonian South Province (Permit Number 1224-08/PS).~~

Compilation of the taxon set

The ingroup species of the Gobioidei used in this study comprise 29 extant species; the apogonid *Sphaeramia nematoptera* is used as outgroup (Table 1). The Odontobutidae, Milyeringidae, Butidae, and Thalasseleotridae are each represented by two species, the Rhyacichthyidae and the Eleotridae each by three, the Oxudercidae by five, and the Gobiidae by ten species. Criteria for species selection were the availability of 'total-evidence data' including molecular as well as morphological data of the skeleton and ~~, osteological, meristic, and~~ otolith data. Morphological data of ~~three~~ four species were coded based solely on published information: the odontobutid *Odontobutis obscurus* (data from [44, 54, 55]), the milyeringid *Typhleotris madagascariensis* (data from [56]), the thalasseleotridid *Thalasseleotris iota* (data from [57-59]), and the oxudercid *Eucyclogobius newberryi* (data from [60, 61]); no otolith data~~s~~ were ~~not~~ available for *T. madagascariensis*~~the latter two~~. This selection of extant species, although modest in terms of species numbers of Oxudercidae and

Gobiidae, was sufficient to achieve a molecular ‘backbone’ as its phylogenetic analysis produced a well-resolved tree that fully agrees with published hypotheses (see Results).

Ten fossil gobioid species were added to the extant taxon set (Table 1). We selected those that we had examined in previous works [11, 42-44, 46, 62], and added also the oldest known putative gobioid so far, †*Carlomonnius quasigobius* (Table 1). A short overview of all fossil species is provided in Appendix 1.

Table 1. Species and specimens used in this study. Birdsong et al. [60], Hoese and Gill [28] and Gill and Mooi [15] are used for morphology of many or all species. Colour codes ~~F~~for molecular data: Agorreta et al. [12] =green; Near et al. [63] =blue; Thacker et al. [10] =red~~ are used~~; for not color-coded cells see entries in Genbank via given accession numbers.

**Family**	**Species**	**Morphology**	**rRNA**	***cytb***	***rag1***	***zic1***	***sreb2***
Fossil	†*Carlomonnius quasigobius* Bannikov and Carnevale, 2016	Bannikov and Carnevale 2016 [64]	n/a	n/a	n/a	n/a	n/a
Fossil	†*Eleogobius brevis* (Agassiz, 1839)	NHMUK PV OR 42779–80; Gierl and Reichenbacher 2015 [42]	n/a	n/a	n/a	n/a	n/a
Fossil	†*Eleogobius gaudanti* Gierl and Reichenbacher, 2015	Gierl and Reichenbacher 2015 [65]	n/a	n/a	n/a	n/a	n/a
Fossil	†“*Gobius*” *francofurtanus* Koken, 1891	Weiler 1963; Gierl 2012 [62, 66]	n/a	n/a	n/a	n/a	n/a
Fossil	†*Gobius jarosi* Prikryl and Reichenbacher, 2018	Reichenbacher et al. 2018 [46]	n/a	n/a	n/a	n/a	n/a

Fossil	†*Lepidocottus aries* (Agassiz, 1839)	Gierl et al. 2013 [44]	n/a	n/a	n/a	n/a	n/a
Fossil	†*Paralates bleicheri* Sauvage, 1883	Gierl and Reichenbacher 2017 [43]	n/a	n/a	n/a	n/a	n/a
Fossil	†*Paralates chapelcorneri* Gierl and Reichenbacher, 2017	Gierl and Reichenbacher 2017 [43]	n/a	n/a	n/a	n/a	n/a
Fossil	†*Pirskenius diatomaceus* Obrhelová, 1961	Obrhelová 1961; Přikryl 2014; Reichenbacher et al. 2020 [11, 49, 67]	n/a	n/a	n/a	n/a	n/a
Fossil	†*Pirskenius radoni* Přikryl 2014	Přikryl 2014; Reichenbacher et al. 2020 [11, 67]	n/a	n/a	n/a	n/a	n/a
Gobiidae	*Amblygobius phalaena* (Valenciennes, 1837)	ZSM-PIS-P-GO-0594, -0600; Harrison 1989 [26]	KF415301	KF415498	KF415693	KF415897	KF416109
Gobiidae	*Aphia minuta* (Risso, 1810)	NMP 6V; Rojo 1985; Birdsong et al. 1988; Harrison 1989; La Mesa et al. 2005 [26, 60, 68, 69]	KF415305	FR851417	KF415697	KF415901	KF416113

Gobiidae	*Asterropteryx semipunctata* Rüppell, 1830	SMNS-Z-PI-026591, -005882 ~~(skeletal data);~~ ~~otolith from Schwarzhans et al. 2020: pl. 7, fig. 5;~~ Birdsong et al. 1988; Van Tassell et al. 1988; Harrison 1989 (skeleton data); Schwarzhans et al. 2020: pl. 7, fig. 5 (otolith) [26, 50, 60, 70]	KF415309	KF415506	KF415701	KF415906	KF416117
Gobiidae	*Cryptocentrus cinctus* (Herre, 1936)	ZSM-PIS-P-GO-0619, -0639; Karplus and Thompson 2011 [9]	KF415340	KF415536	KF415732	KF415940	KF416150
Gobiidae	*Discordipinna griessingeri* Hoese and Fourmanoir, 1978	ZSM-PIS-P-GO-0632, -0633; Hoese and Fourmanoir 1978; Birdsong et al. 1988 [60, 71]	KF415345	KF415540	KF415737	KF415945	KF416155
Gobiidae	*Glossogobius giuris* (Hamilton, 1822)	ZM-CBSU; Harrison 1989; Esmaeili et al. 2009 [26, 72]	KF415369	KF415566	KF415767	KF415972	KF416185
Gobiidae	*Gobius niger* Linnaeus, 1758	NMP6V 146072, - 146073; Harrison 1989 [26]	KF415385	KF415583	KF415786	KF415990	KF416203
Gobiidae	*Lesueurigobius sanzi* (de Buen, 1918)	ZSM-PIS-035529_1, _4 ~~(skeletal data)~~; ~~otolith from Schwarzhans et al. (2020: pl. 7, fig. 5);~~ Miller 1986; Birdsong et al. 1988; Harrison 1989; McKay and Miller 1997 (skeleton data); Schwarzhans et al. 2020: pl. 2, fig. 3 (otolith) [26, 60, 73, 74]	KF415406	KF415603	KF415808	KF416012	

Field Code Changed

Gobiida e	*Ptereleotris evides* (Jordan and Hubbs, 1925)	ZSM-PIS-P-GO-0587, -0588; Randall and Hoese 1985 [75]			KF1 413 41	KF1 406 23	KF1 402 63
Gobiida e	*Tigrigobius multifasciatus* (Steindachner, 1876)	ZSM-PIS-P-GO-0622, -0642	AF4 911 02	AY8 464 02	KF4 158 78	KF4 160 88	KF4 163 02
Oxuderc idae	*Awaous flavus* (Valenciennes, 1837)	ZSM-PIS-43853 (P-GO-1050, -1051)	KF4 153 11	KF4 155 08	KF4 157 03	KF4 159 08	KF4 161 19
Oxuderc idae	*Chlamydogobius eremius* (Zietz, 1896)	ZSM-PIS-43854 (P-GO-1052, -1053); Miller 1987 [76]	KF4 153 29	KF4 155 26	KF4 157 20	KF4 159 28	KF4 161 38
Oxuderc idae	*Eucyclogobius newberryi* (Girard, 1856)	Birdsong et al. 1988; Kindermann et al. 2007 [60, 61]; otolith from the collection of W. Schwarzhans ex LACM-58239	KF4 153 55	EU3 809 42	KF4 157 51	KF4 159 58	KF4 161 69
Oxuderc idae	*Gobioides broussonnetii* Lacepède, 1800	ZSM-PIS-43852 (P-GO-1048, -1049)	KF4 153 74		KF4 157 72	KF4 159 77	KF4 161 89

Oxudercidae	*Pomatoschistus flavescens* (Fabricius, 1779)	ZSM-PIS-043982 (2); Vigo, Spain, Sanda, Prag; Mestermann and Zander 1984; Harrison 1989 [26, 77]	KF415386	KF415584	KF415787	KF415991	KF416204
Thalasseleotridae	*Grahamichthys* ~~*radiatus*~~ sp. ~~(Valenciennes, 1837)~~	ZSM-P-GO-0783; Whitley 1956; McDowall 1965; Akihito 1986; Gierl and Reichenbacher 2015; ~~Schwarzhans 2019~~ [42, 54, 78-80]			KT266412	KT266520	KT266467
Thalasseleotridae	*Thalasseleotris iota* Hoese and Roberts, 2005	~~ZSM-P-GO-766;~~ Hoese and Larson 1987; Hoese and Roberts 2005 (skeleton data); Schwarzhans 2019: fig. 99.10 (otolith) [57-59]~~; Hoese & Roberts 2005; Schwarzhans 2019~~			KT266413	KT266521	
Butidae	*Kribia nana* (Boulenger, 1901)	MRAC (1-5); Wongrat 1977 [81]		AY722211	KF235468		
Butidae	*Oxyeleotris marmorata* (Bleeker, 1852)	ZSM-PIS-43857 (P-GO-1058, -1059); Regan 1911; Harrison 1989 [20, 26]	KF415429	KF415623	KF415829	KF416035	KF416250
Eleotridae	*Dormitator maculatus* (Bloch, 1792)	ZSM-PIS-009383 – ~~8~~5; Harrison 1989 (skelet~~a~~on data) [26]; otolith from the collection of W. Schwarzhans ex LACM (collection Fitch)~~; Harrison 1989 [26]~~	KF415347	KF415542	KF415739	KF415947	KF416157

Field Code Changed

Field Code Changed

Eleotridae	*Hypseleotris compressa* (Krefft, 1864)	ZSM-PIS-43863 (P-GO-1068, -1069); Akihito 1986; Akihito et al. 2000; Thacker and Unmack 2005 [14, 25, 54]	KF415398	KF415596	KF415800	KF416004	KF416217
Eleotridae	*Tateurndina ocellicauda* Nichols, 1955	ZSM-PIS-43855 (P-GO-1054, -1055)	KF415480	KF415672	KF415875	KF416085	KF416299
Milyeringidae	*Milyeringa veritas* Whitley, 1945	WAM-BES1151(B), -BES18735, -BES9768.2; Larson et al. 2013 [82]		HM590598	KT266404	KT266511	KT266459
Milyeringidae	*Typhleotris madagascariensis* Petit, 1933	FMNH 116494-98 (skelet~~al~~on data only); Sparks and Chakrabarty 2012 [56]		JQ619661	KY981273		
Odontobutidae	*Odontobutis obscurus* (Temminck and Schlegel, 1845)	IRSNB ex BMNH 1983.11.4 (otolith only); Iwata et al. 1985; Akihito 1986; ~~Gierl et al. 2013;~~ Iwata and Sakai 2002; Gierl et al. 2013 [44, 54, 55, 83]	KF415424	KF415618	KF415825	KF416030	KF416245
Odontobutidae	*Perccottus glenii* Dybowski, 1877	ZSM-PIS-43867 (P-GO-1078, -1079); Akihito 1986; Birdsong et al. 1988; Harrison 1989 [26, 54, 60]	KF415440	KF415632	KF415837	KF416044	JX190055

Field Code Changed

Field Code Changed

Field Code Changed

Rhyacic<br>hthyida<br>e	*Rhyacichthys aspro*<br>(Valenciennes, 1837)	ZSM-PIS-044262 (BAyFi 13481); Miller 1973; Birdsong et al. 1988 [24, 60]	KF4<br>154<br>62	KF4<br>156<br>54	KF4<br>158<br>58	KF4<br>160<br>66	KF4<br>162<br>82
Rhyacic<br>hthyida<br>e	*Rhyacichthys guilberti*<br>Dingerkus and Séret,<br>1992	MNHN 2019-0113-1, MNHN 2019-0113-2		KF6<br>690<br>52			
Rhyacic<br>hthyida<br>e	*Protogobius attiti*<br>Watson and Pöllabauer,<br>1998	MNHN 2019-0112; Akihito et al. 2000; Shibukawa et al. 2001 [14, 84]		AB0<br>212<br>57			
Apogoni<br>dae	*Sphaeramia*<br>*nematoptera* (Bleeker,<br>1856)	ZSM-PIS-P-GO-0621, -0634; McAllister 1968; Fraser 1972; Bergman 2004 [19, 85, 86]	AB8<br>896<br>73		KT2<br>664<br>01	KT2<br>665<br>08	KT2<br>664<br>56

Study and compilation of morphological characters

Literature data, when available, ~~was~~ were used to compile phylogenetically informative morphological characters for the extant species (see ~~Supplementary~~ Table 1 for details). Additionally, X-ray images were produced for the extant species with a Faxitron Ultrafocus facility at the ZSM and examined to determine numbers of vertebrae, fin elements and pterygiophores, and configuration of the caudal skeleton. After radiography, otoliths were extracted from the same specimens and prepared for scanning electron microscopy (SEM) imaging (using a HITACHI SU 5000 Schottky FE-SEM at the Department of Earth- and Environmental Sciences, LMU Munich). SEM images served as the basis for the identification of the otolith characters (Fig. 2), which are used here for the first time within a phylogenetic matrix. They include (i) the overall otolith shape (~~five~~ six character states: trapezoid/triangular;~~,~~ long rectangular;~~,~~ high rectangular~~/higher than long~~;~~,~~ quadratic~~/slightly higher/slightly longer~~;~~,~~ rounded; longish-ovate), (ii) the posterodorsal projection (two states: present; ~~or~~ absent), (iii) ~~the sulcus position (centred or shifted anteriorly), and (iv)~~ the sulcus shape (three states: perciform-like~~other~~, shoe sole, shoe sole/specialized), and (iv) the sulcus shape and position (three states: shoe sole + centred, shoe sole + shifted anteriorly, not shoe sole). Plesiomorphic otolith character states were defined according to the condition seen in the otolith of the outgroup (see Supplementary Table for details). ~~.~~The otoliths of the included extant gobioid species are shown in Figure 3, the otolith of the outgroup species *Sphaeramia nematoptera* is depicted in Figure 2. For the fossil species, skeletal and otolith characters were largely compiled from previous works, but some additional characters could also be added (see Results).

Fig 2. Otolith terminology and otolith characters and character states used here for the phylogenetic analyses. Images depict left otoliths (sagittae) in medial view. Scale bars: 0.5 mm.

A total of ~~54~~ 48 morphological characters were assembled. ~~Forty~~Thirty-~~three~~ eight characters concern bony structures of the skeleton, four characters relate to cartilage, membrane, or tendon configurations, four refer to otolith morphology, two concern the presence and type of ctenii on the scales, and one is a morphometric character (see Supplementary Table Part B -for ~~details~~all characters). Character states were determined according to ~~the results of the~~ literature data and our morphological investigations (based on X-rays, SEM images of otoliths) (see Supplementary Table Part A for details). ~~Table 1 and the Supplementary Table provide details of the data sources of the characters of each species.~~

All taxa and characters were assembled in Mesquite 3.61 [87]. We used presence/absence coding (1/0) or up to ten states, depending on the character (see Supplementary Table).

~~Twenty-one characters were coded according to previous works, i.e. we assigned the extant species the character state that is apomorphic for its family, following Hoese and Gill [28] and Gill and Mooi [15]. The remaining character states were determined according to the results of the literature data and our morphological investigations (based on X-rays, SEM images). Table 1 and the Supplementary Table provide details of the data sources of the characters of each species.~~

Preparation of the molecular data matrix

Molecular sequence data for extant species were assembled based on previously published sequences of five markers: *rDNA* (12S rRNA, tRNA-Val, 16S rRNA), *cytb*, *rag1*, *zic1*, and *sreb2*. The sequences were mainly from the study of Agorreta et al. [12],

supplemented by some data from Near et al. [63] and Thacker et al. [10]. Molecular data were downloaded from GenBank, aligned in AliView 1.26 [88] with MUSCLE [89], followed by manual adjustment and exclusion of ambiguous regions where necessary. Individual gene alignments were then concatenated into a supermatrix (6271 bp) with SeaView 5.0.4 [90]. For details and GenBank accession numbers see Table 1. All data matrices are available on figshare (https://figshare.com/s/ed10b9a5ac382f856a20).

Phylogenetic analyses

We conducted phylogenetic analyses for the extant species based on the morphological character matrix, the molecular supermatrix, and based on the combined molecular and morphological (total evidence) datasets. Adding all ten fossils ~~The fossil species were added~~ to the total evidence character set resulted in a collapse of the molecular backbone (see below). Therefore we us~~ing~~ed a step-wise approach: (i) a single fossil species was added, (ii) two fossil species of the same genus were added, (iii) four ~~to ten~~ fossil species were added. We used the Bayesian Markov Chain Monte Carlo (MCMC) approach implemented in MrBayes versions 3.2.6 and 3.2.7a [91], as well as implied-weights maximum parsimony (IW-MP, [92]) implemented in TNT 1.5 [93] to infer phylogenies.

In MrBayes, we assigned the Mkv+G model [94, 95] to the morphological data, and separate GTR+G models [95, 96] to each molecular partition. For each analysis, we ran 2 x 4 MCMC chains in parallel for 5 x 10^6 generations. We used the "sump" command in MrBayes as well as Tracer 1.7.1 [97] to check for convergence and discarded the first 10% of samples of each analysis as burn-in before summarizing the remaining samples in 50% majority-rule consensus (MRC) trees including posterior probability (PP) values for all clades.

In TNT, we employed new-technology searches (with sectorial search, ratchet, drift, and tree fusing enabled; init. addseqs = 100; find min. length = 10) and a concavity constant of K = 12. A 50% majority-rule consensus tree was calculated if more than one most

parsimonious tree was found. For assessing clade support in the IW-MP analysis, we used standard bootstrap resampling [98] (500 replicates; new technology search; init. addseq = 10; find min. length = 5). Phylogenetic trees were visualized in FigTree 1.4.4 [99]; tree files are available on figshare (https://figshare.com/s/ed10b9a5ac382f856a20).

Results

Skeleton data

The character state of each character of each species (extant and fossil) is provided in the Supplementary Table; unknown character states were coded with a question mark.

Three characters could be coded for the first time for †*Eleogobis brevis* based on the specimens NHMUK PV OR 42779 and 42780, deposited in the Natural History Museum in London: the pterygiophore formula (3-12210), the presence of a single anal fin pterygiophore inserting before the haemal spine of the first caudal vertebra (AP = 1), and the position of the penultimate branchiostegal on the ceratohyal. Re-inspection of the type specimens of †*Gobius jarosi* revealed that in this species the penultimate branchiostegal is located on the ceratohyal. Finally, a count of three to four anal fin pterygiophores (AP = 3-4) could be determined for †*Lepidocottus aries* based on re-inspection of the specimens used in Gierl et al. [44].

Otolith data

Figure 3 depicts the otoliths of the extant species included in this study. Short descriptions of the otoliths are given below.

Fig 3. Otoliths of the extant gobioid species used in this study. Images depict left otoliths (sagittae) in medial view, except for *Lesueurigobius sanzi*, *Asterropteryx semipunctata*, *Dormitator maculatus* and *Odontobutis obscurus*, which represent right sagittae that were

mirrored for better comparison. Otoliths were not available for the milyeringid *Typhleotris madagascariensis*~~ and the oxudercid *Eucyclogobius newberryi*~~. For sources of otoliths see Table 1. Scale bars: 0.5 mm.

Family Gobiidae

Amblygobius phalaena (Valenciennes, 1837). – High-rectangular~~Irregular, high~~, margins crenate, dorsal rim strongly lobed, pronounced posterodorsal projection present. Sulcus centered~~median~~, shoe-sole shape with slender cauda and wide ostium with dorsal lobe.

Aphia minuta (Risso, 1810). – Rounded~~Discoidal~~, margins smooth, short projection slightly below level of ostium tip. Sulcus shifted anteriorly~~median~~, rather shoe-sole shape, cauda strongly reduced, ostium with dorsal lobe and pointed tip.

Asterropteryx semipunctata Rüppell, 1830. – Rectangular, higher than long. Dorsal, anterior and ventral margins slightly crenulate, small posterodorsal projection. Sulcus centered~~median~~, shoe-sole shape, with rounded cauda and ostium. See Schwarzhans et al. [50] for additional details of the same otolith.

Cryptocentrus cinctus (Herre, 1936). – High-r~~R~~ectangular~~, higher than long~~, margins smooth, with a projection in the middle of the dorsal margin and a median constriction on anterior and posterior rims. Sulcus centered~~median~~, shoe-sole shape with small, rounded cauda and a broad ostium with ostial lobe.

Discordipinna griessingeri Hoese and Fourmanoir, 1978. – Long-r~~R~~ectangular, smooth margins with median constrictions anterior and posterior defining four projections. Sulcus centered, shortened, ~~submedian, ~~oval specialized, surrounded by a bulging crista.

Glossogobius giuris (Hamilton, 1822). – Long-rectangular~~Oval~~, slightly crenate margins, posterodorsal projection and a sub-median projection on the posterior rim. Sulcus centered~~median~~, shoe-sole shape, cauda elongated, ostium with strong dorsal lobe.

Gobius niger Linnaeus, 1758. – Long-rRectangular, dorsal margin slightly undulated, prominent posterodorsal and posteroventral projections, small anteroventral projection. Sulcus centeredmedian, shoe-sole shape, elongated with rounded cauda and ostium with dorsal lobe.

Lesueurigobius sanzi (de Buen 1918) – QuadraticSquarishQuadratic/slightly longer with rounded dorsal margin, dorsal and anterior margins faintly lobed, other margins smooth, submedian constriction on posterior margin. Posterodorsal projection short and rounded, weak anteroventral projection. Sulcus centeredmedian, shoe-sole shape, rounded cauda, ostium with pointed tip. See Schwarzhans et al. [50] for additional details of the same otolith.

Ptereleotris evides (Jordan and Hubbs, 1925). – High-rRectangular, higher than long, margins sinuate; small posteroventral and more pronounced anteroventral projection. Sulcus centeredmedian, shoe-sole shape, with rounded cauda and ostium.

Tigrigobius multifasciatus (Steindachner, 1876). – QuadraticSquaredish, margins undulated; , cusp on dorsal rim. Sulcus shifted anteriorlymedian, shoe-sole shape, cauda rounded, ostium with pronounced elongate tip.

Family Oxudercidae

Awaous flavus (Valenciennes, 1837). – High-rRectangular, dorsal and posterior margins crenate. Sulcus centeredmedian, shoe-sole shape. Cauda and ostium rounded, ostium with marked dorsal lobe.

Chlamydogobius eremius (Zietz, 1896). – QuadraticSquarished, margins slightly undulated, small posterodorsal projection. Sulcus centeredmedian, shoe-sole shape, with small, rounded cauda and broad ostium with marked dorsal lobe.

Eucyclogobius newberryi (Girard, 1856). – QuadraticSquarish with slightly curved dorsal margin, small posterodorsal projection, sulcus centeredmedian, shoe-sole shape, with well-developed cauda and ostium.

Gobioides broussonnetii Lacepède, 1800. – Rounded~~Discoidal~~, margins irregularly crenate, small posterodorsal cusp. Sulcus centered~~median~~, shoe-sole shape, rounded cauda, large ostium with rounded tip.

Pomatoschistus flavescens (Fabricius, 1779). – Quadratic~~Oval~~, margins smooth. Sulcus centered~~median~~, shoe-sole shape, weakly developed cauda, rounded ostium.

Family Thalasseleotrididae

Grahamichthys ~~*radiatus* (Valenciennes, 1837)~~ sp. – Rounded~~Discoidal~~, all margins smooth, no projections. Sulcus centered~~median~~, shoe-sole shape, cauda and ostium rounded.

Thalasseleotris iota Hoese and Roberts, 2005. – Long-~~R~~rectangular, margins ~~rather~~ smooth, with ~~, dorsal margin undulated. Small~~ prominent ~~posterodorsal~~ praeventral projection~~-~~ and slightly~~present,~~ rounded ~~anteroventral~~ anterodorsal expansion~~ present~~. Sulcus centered~~median~~, shoe-sole shape, cauda and ostium ~~rounded~~slender. See Schwarzhans [58] for additional details of the same otolith.

Family Butidae

Kribia cf. *nana* (Boulenger, 1901). – Long-r~~R~~ectangular, all margins relatively smooth, small posterodorsal projection, posteroventral expansion (bulge). Sulcus centered~~median~~, ~~rounded-to-~~shoe-sole shape, slightly shifted anteriorly. Rounded cauda about half the length of the ostium.

Oxyeleotris marmorata (Bleeker, 1852). – Long-r~~R~~ectangular~~, longer than high~~, anterior and posterior margins distinctly incised in the middle, pointed posterodorsal and rounded anterodorsal projections. Sulcus centered~~median~~, slender-to-shoe-sole shape, slightly shifted anteriorly.

Family Eleotridae

Dormitator maculatus (Bloch, 1792). – Long-r~~R~~ectangular, dorsally rounded~~. higher than long~~. Anterior and ventral margins finely crenulated, other margins smooth. Sulcus centered~~median~~, shoe-sole shape~~,~~; cauda almost as large as ostium and posteriorly pointed. *Hypseleotris compressa* (Krefft, 1864). – High-r~~R~~ectangular~~, higher than long~~. All margins smooth, anterior and posterior margins with sub-median constriction at the level of the sulcus. Sulcus centered~~median~~, shoe-sole shape, rounded cauda. Ostium with angled dorsal lobe. *Tateurndina ocellicauda* Nichols, 1955. – High-r~~R~~ectangular~~, higher than long~~, all margins relatively smooth except for dorsal margin, which is serrate. Sulcus centered~~median~~, rounded-to-shoe-sole shape, cauda and ostium only weakly differentiated.

Family Milyeringidae

Milyeringa veritas Whitley, 1945. – Trapezoid ~~quadrate~~and smooth with posteroventral projection and dorsally pointing anterodorsal projection. Sulcus centered~~sub-median~~, rounded-to-shoe-sole shape, surrounded by a crista, cauda and ostium not clearly differentiated.

Family Odontobutidae

Odontobutis obscurus (Temminck and Schlegel, 1845). – T~~Irregular t~~rapezoid, dorsal margin with tip in its posterior half, anterior margin with spine-like dorsal projection, ventral margin crenulated and with spine-like posterior projection, posterior margin deeply sinuate. Sulcus centered, supra-median, relatively small, perciform-like. Cauda slender, posteriorly bent, ostium rounded and distant from anterior rim.

Perccottus glenii Dybowski, 1877. – T~~Irregular t~~rapezoid with sinuate dorsal and ventral margins, posterior margin with median notch, anterior margin with sub-median notch. Sulcus centered~~sub-median~~, rounded-to-shoe-sole shape, cauda slightly shorter than ostium.

Family Rhyacichthyidae

Protogobius attiti Watson and Pöllabauer, 1998. – Trapezoid/triangular~~ in overall outline~~, rounded with short median projection on posterior margin, dorsal margin smooth, ventral margin lobed. Sulcus shifted anteriorly~~median~~, perciform-like with slender, relatively short cauda and broad ostium~~, ~~; ostium opened to anterior rim.

Rhyacichthys aspro (Valenciennes, 1837). – Trapezoid/triangular with short median projection on posterior and dorsal margins, dorsal margin slightly undulated with prominent anterodorsal bulge, ventral margin crenate. Sulcus shifted anteriorly~~ median~~, perciform-like with slender, relatively short cauda and broad ostium~~. O~~; ostium anteriorly closed.

Rhyacichthys guilberti Dingerkus and Séret, 1992. – Trapezoid/triangular~~ to slightly rounded~~, ventral margin lobed, dorsal margin smooth. Sulcus shifted anteriorly~~ median~~, perciform-like with slender, relatively short cauda and broad ostium~~,~~; ostium opened to anterior rim.;

Family Apogonidae (outgroup)

Sphaeramia nematoptera (Bleeker, 1856). – Overall shape ~~shield-like~~longish-ovate, with projections on the shallower dorsal rim. Perciform sulcus slightly above the middle, with broad ostium and slender cauda which are of equal length.

Phylogenetic analyses

Phylogenetic relationships ~~based on~~ of the extant species

~~inferred from molecular data~~

Formatted: Font: 12 pt

Molecular data. – The Bayesian phylogeny based on molecular data (Fig 4A) recovers all families as monophyletic. Notably, although we used a restricted number of species, the tree is completely congruent with the trees published by Agorreta et al. [12] and Thacker et al. [10]: Rhyacichthyidae and Odontobutidae are sister groups, and together they are sister to the rest of the gobioid families. Milyeringidae, Eleotridae, Butidae, and Thalasseleotrididae are successive sister groups to the 5brG clade, which is composed of well-supported Oxudercidae and Gobiidae.

Maximum Parsimony analysis produced a single most parsimonious tree (Fig S1), which is similar to the Bayesian tree, but some nodes have poor bootstrap support (BS). The tree is topologically identical to the Bayesian tree on family-level, only within Gobiidae there are some minor differences concerning the positions of *Amblygobius* and *Asterropteryx*; BS for several nodes is rather low compared to the support in the Bayesian tree.

Morphological data. –

~~Phylogenetic relationships inferred from morphological data~~

~~Morphological phylogeny based on the extant species~~

In the Bayesian phylogeny restricted to the extant species, the 5brG clade (Gobiidae + Oxudercidae) is recovered with maximum support, but the internal structure of the clade is completely unresolved (Fig ~~5A~~4B). The thalasseleotridid species *G. radiatus* and *Th. iota* group together and are ~~successive~~ sister ~~groups~~ to 5brG~~, thus rendering the Thalasseleotrididae paraphyletic (PP = 0.71).~~ ~~. However, t~~The Thalasseleotrididae + 5brG

clade is maximally supported, consistent with molecular phylogenetic results. Also, similar to the established molecular phylogeny, Eleotridae, Butidae, and Milyeringidae are closely related to that clade (1.00). However, only Eleotridae and Milyeringidae are recovered as monophyletic (0.~~82~~91, 0.82), whereas Butidae are paraphyletic ~~and monophyly of Milyeringidae could not be resolved~~(Fig 4B). Monophyly of Odontobutidae is also not resolved, and in contrast to the molecular phylogeny the odontobutid species are closer to the above assemblage (1.00) instead of being sister to Rhyacichthyidae, which are ~~also~~ recovered paraphyletic (Fig 4B).

The Maximum Parsimony analysis ~~produced~~ recovered one~~three~~ most parsimonious tree~~s. The resulting strict consensus tree~~ (Fig S2~~A~~), which is overall similar to the Bayesian tree, but many nodes have very poor bootstrap support (BS). W~~Resolution w~~ithin the 5brG clade (BS = 95%) the ~~is better than in the Bayesian tree, but~~ reciprocal monophyly of Gobiidae and Oxudercidae is not recovered. Thalasseleotrididae is monophyletic and highly supported as sister to 5brG (99%). As in the Bayesian tree, among the remaining families ~~only~~Eleotridae (71%) and Milyeringidae (52%) are~~is~~ supported as monophyletic ~~(56%)~~.

Total-evidence approach. – The Bayesian phylogeny inferred from the total-evidence dataset (=combined molecular and morphological data) including only extant species ~~(Fig 6A)~~ is topologically identical to the molecular phylogeny ~~(Fig 4)~~ but shows maximum support for Rhyacichthyidae and Rhyacichthyidae + Odontobutidae (1.00 and 1.00 vs. 0.62 and 0.64, respectively) (Fig S3).

The Maximum Parsimony ~~(MP)~~ analysis ~~produced~~ recovered one most parsimonious tree (Fig S4~~3A~~), which is similar to the Bayesian tree. Most nodes have good bootstrap support ~~(BS)~~, except for some clades within ~~the~~ 5brG that are weakly supported. Concerning the topology, the only difference ~~to the molecular tree~~ ~~areis~~is that the positions of the oxudercid genera ~~the slightly~~~~switch~~ ~~changed positions~~ ~~of~~ *Eucyclogobius*, ~~and~~ *Pomatoschistus*

and *Chlamydogobius* ,~~Pterelotris evides within Gobiidae~~are resolved while they form a polytomy in the Bayesian tree (see Fig S3 vs. Fig S4).~~.~~

Formatted: Font: Not Italic

Fig 4. 50% MRC Bayesian tree with posterior probabilities. A ~~(average standard deviation of split frequencies between two independent runs [ASDSF] = 0.000647);~~ Tree based on published DNA data of the 29 gobioid species used in this study (average standard deviation of split frequencies between two independent runs [ASDSF] = 0.003776; for sources of molecular data see Table 1). **B** Tree based on morphological characters of the extant species only (ASDSF = 0.002446~~0.002449~~). Scale bar, average number of substitutions per site respectively character changes per character.

Phylogen~~eti~~ies~~c~~ ~~relationships~~ including extant ~~plus~~ and all ten ~~10~~fossil species

Morphological data. – In the Bayesian phylogeny, the 5brG clade (Gobiidae + Oxudercidae) – including the two fossil *Gobius* spp. – is recovered only with moderate support (0.702~~1~~), and internal relationships are again unresolved (Fig 5~~B~~A). The rest of the tree is topologically similar to the one based on the morphology of the extant species only (Fig ~~5A~~4B), ~~but~~ Thalasseleotrididae are now recovered ~~as monophyletic~~ with ~~weak~~ slightly higher support (0.~~57~~80~~1~~ vs. 0.77). The two †*Eleogobius* spp. are placed ~~paraphyletically (0.55)~~ between Thalasseleotrididae and 5brG (0.60), but monophyly of the genus is not resolved. The †*Pirskenius* and †*Paralates* spp. form a weakly supported clade (0.64~~57~~) that is sister to the above assemblage (0.73~~69~~), but only †*Pirskenius* (i.e., †Pirskeniidae) is resolved as monophyletic (0.87~~1~~). The remaining two fossil species, †*Lepidocottus aries* and †*Carlomonnius quasigobius*, are clearly more ~~closely~~ related to Thalasseleotrididae + 5brG

than to Rhyacichthyidae and Odontobutidae (0.8~~2~~4~~1~~), but their exact placements are not resolved.

The Maximum Parsimony analysis using the same data set ~~produced~~ recovered six~~two~~ most parsimonious trees. The resulting ~~strict~~50% majority rule consensus tree (Fig S~~2B~~5) is overall consistent with the one based on extant species only (Fig S2) but is very poorly resolved on a deeper level; most nodes have BS < 50%. The two fossil *Gobius* spp. are included in the 5brG ~~but neither is closely related to the extant~~in a clade with *Gobius niger*, *Discordipinna* and *Lesueurigobius*. The two †*Eleogobius* spp. are sister to 5brG but monophyly of the genus remains unresolved. †*C*~~.~~ *arlomonnius quasigobius* is placed in a clade containing Eleotridae ~~, Milyeringidae,~~ and *Kribia nana* (Butidae). †*Pirskenius* is monophyletic (64~~51~~%) but its position, as well as that of the remaining three fossil species, is not further resolved.

Total~~-~~ evidence ~~phylogeny including all ten fossil species~~approach. –
The Bayesian phylogenetic analysis of the total-evidence dataset including all 29 extant and the ten fossil species produced a consensus tree with a largely collapsed backbone (Fig 5B~~9~~) compared to the tree based on only the morphological data set of the same taxa ~~molecular phylogeny~~ (Fig ~~4~~5A). It ~~and the combined analysis including only extant taxa (Fig 5A),~~ contains~~ing~~ numerous polytomies and shows~~ing~~ poor support for many deeper nodes. ~~Nevertheless, some results are noteworthy.~~ T~~Although t~~he 5brG clade (Gobiidae + Oxudercidae) forms a polytomy with the Thalasseleotrididae, the Butidae, the clade of †*Paralates* + †*Pirskenius* and three further fossil species (†*Eleogobius* brevis, †*E. gaudanti*, †“*Gobius*” *francofurtanus*). Nevertheless, several groups can be~~is only~~ recovered within this polytomy ~~with weak support (0.51),~~. Oxudercidae, which were~~internal relationships are better~~not resolved ~~than~~ in the phylogen~~ies~~y based only on morphology (Fig~~s~~ ~~5~~5A~~A, B~~), are now recovered with high support (0.93), ~~, recovering Oxudercidae with high support (0.96),~~

Commented [BR1]: 5A stimmt

as well as some clades ~~two further clades~~ within Gobiidae that correspond to the molecular phylogeny, i.e. the *Aphia* (0.89) and *Glossogobius* (0.72) clades. †*Gobius jarosi* is weakly (0.5~~2~~5) placed as sister to the extant *G. niger*~~, whereas the position of †*G. francofurtanus* within 5brG is not resolved. †*Eleogobius brevis* is now included in 5brG (unresolved position) whereas †*E. gaudanti* is placed farther rootwards in the phylogeny~~. The clade of †*Pirskenius* + †*Paralates* ~~clade~~ (see above) is ~~still~~ recovered with slightly weaker support (~~also~~ 0.~~57~~59 vs. 0.64), while ~~and shows even higher~~ support for monophyly of †Pirskeniidae is slightly increased (0.9~~2~~3 vs. 0.87)~~, but its exact position is poorly resolved~~. ~~As in the total evidence phylogenies including a single species each,~~ †*Lepidocottus aries* and †*Carlomonnius quasigobius* are resolved as members of Butidae, albeit with low support (0.~~59~~61).

The consensus tree of the two most parsimonious ~~Maximum Parsimony~~ trees of the same data set (Fig S~~8~~6) is even less well resolved, with very weak BS for many nodes. The 5brG clade is recovered, with reciprocally monophyletic Oxudercidae and Gobiidae. In contrast to the Bayesian tree, †"*G*obius~~,~~" *francofurtanus* is sister to *G*~~.~~ obius *niger* (< 50%) (vs. not resolved in the Bayesian tree), whereas †*G*~~.~~ obius *jarosi* is sister to *Lesueurigobius* (also < 50%) (vs. sister to *G. niger*). †*Eleogobius* is monophyletic and sister to the 5brG clade, but again BS for this is negligible. †*Pirskenius* is monophyletic (65~~50~~%) but its position, as well as that of the remaining four fossil species, is not further resolved.

Formatted: Font: Not Italic

Fig 5. 50% MRC Bayesian trees with posterior probabilities based on ~~morphological~~ the extant plus ten fossil species~~characters~~. **A** Tree based on only the morphological data set ~~based on the extant species only~~ (ASDSF = 0.~~002449~~006790). **B** Tree based on the total evidence data set ~~based on the extant species plus ten fossil species (indicated with (†;~~ ASDSF = 0.~~004479~~010856). Scale bars, average number of substitutions per site respectively character changes per character.

~~Phylogenies inferred from total evidence data~~

Formatted: Font: 14 pt

~~Total-evidence phylogeny based on the extant species~~

Formatted: Font: 14 pt

Total-evidence phylogenies including one to four fossil species

In the following analyses, a single fossil was added to the data set of the extant species and the trees were inferred based on the morphological and molecular data (total-evidence approach).

†*Carlomonnius quasigobius*. –

The Bayesian phylogeny is topologically identical to the molecular and the total evidence phylogenies based on extant species only~~.~~; †*Carlomonnius quasigobius* is placed within Butidae (Fig 6~~6B~~A). Support values are high (>0.90) for most clades, but not for Butidae (0.7~~8~~4 vs. 1.00 in the molecular tree).

The single Maximum Parsimony ~~MP~~ tree retained recovered all recent families, like the Bayesian tree (Fig S7). However, in this tree †*C. quasigobius* is placed as sister to Thalasseleotrididae + 5brG, albeit with very low support (< 50%) ~~(Fig S3B)~~.

~~Total-evidence phylogenies including~~ †*Lepidocottus aries*. –

The Bayesian phylogeny (Fig 6B) and the single Maximum Parsimony ~~MP~~ tree retained (Fig S8) reveal the same topology and almost the same support values as described above for the trees including †*C. quasigobius*~~.~~. ~~The fossil~~ †*Lepidocottus aries* is placed within Butidae in both the Bayesian and the Maximum Parsimony ~~MP~~ tree, with high support values in the former (0.~~81~~95), ~~and~~but very low support in the ~~MP tree~~latter (~~-~~< 50%)~~; Fig 6C, Fig S3C)~~.

Fig 6. 50% MRC Bayesian trees with posterior probabilities based on ~~a~~ the total-evidence ~~approach~~ dataset~~(morphology and DNA)~~. A †*Carlomonnius quasigobius* was added to the extant species (ASDSF = 0.003939). **B** †*Lepidocottus aries* was added to the extant species (ASDSF = 0.000854). Scale bars, average number of substitutions per site respectively character changes per character.

~~Total-evidence phylogenies including~~ †*Gobius jarosi* and †"*Gobius*"~~,~~ *francofurtanus*. –

In the Bayesian tree (Fig 7A), topologies and support values are similar as described above. In comparison to the Bayesian total-evidence phylogeny based on extant taxa only (Fig S3), somewhat decreased support values concern two internal gobiid clades: the *Aphia*-lineage (0.9~~2~~5 vs. 1.00) and the clade containing *Glossogobius* and two members of the *Cryptocentrus*-lineage (0.~~88~~90 vs. 1.00). The two ~~two~~ fossil species ~~*Gobius* species~~ are recovered as successive sister groups to *G. niger* with moderate support (0.7~~1~~7) (Fig ~~6~~5D). In the consensus tree of ~~single retained~~two Maximum Parsimony ~~MP~~ trees (Fig S9), †"*G.*~~.~~*obius*" *francofurtanus* is sister to *Gobius*~~.~~ *niger* while the position of ~~and~~ †*G. jarosi* is unresolved in a clade with~~recovered as sister to~~ *Lesueurigobius sanzi*, *Tigrigobius multifasciatus*, *Asterropteryx semipunctata*, *Amblygobius phalaena*, and *Aphia minuta* ~~(both < 50%)~~ (Fig S~~3D~~9). When only one of the two fossil ~~*Gobius*~~ species is included, a sister-relation with *G. niger* is apparent in each case, in both the Bayesian and the Maximum Parsimony ~~MP~~ tree (Figs S10–S13). ~~Fig S3 E,F.~~

†*Eleogobius brevis* and †*E. gaudanti*. –

Formatted: Font: 12 pt

Formatted: Indent: First line: 0"

The Bayesian phylogeny inferred from the total-evidence dataset including all extant species and either †*E. brevis* or †*E. gaudanti* (Figs ~~7~~S14, 15~~A, B~~) is topologically identical to the molecular phylogeny, and almost no decrease of support values ~~occurs~~is seen. †*Eleogobius brevis* is recovered as sister to *Gobius niger* with ~~high~~ good support (0.~~93~~87) ~~(Fig 7A)~~, while †*E. gaudanti* shows a well-supported (0.8~~3~~7) sister relation to the Thalasseleotrididae ~~(Fig 7B)~~. When both species of †*Eleogobius* are added, they are recovered ~~as successive sister groups to~~in a polytomy with the Thalasseleotrididae ~~, but each with low support~~ (0.~~52, 0.59~~88) (Fig 7~~C~~B), and there is ~~clearly~~ slightly decreased support for the 5brG clade (0.~~63~~90 vs. 1.00), and also for some of the gobiid clades (e.g. *Tigrigobius* + *G. niger*: 0.~~65~~91 vs. 1.00) compared to the total evidence tree using only the extant species (Fig S3).

In the Maximum Parsimony ~~MP~~ trees, when only one of the †*Eleogobius* spp. is included, its position matches that recovered in the Bayesian analyses, albeit with poor support (Figs S16, 17~~4A, B~~). W~~In contrast, w~~hen both species are included, they form a clade~~group together~~ and are sister to the Thalasseleotrididae ~~*Gobius niger*~~ (< 50%, Fig S18~~4C~~).

Fig ~~6~~7. 50% MRC Bayesian trees with posterior probabilities based on ~~a~~ the total-evidence ~~approach~~ dataset~~(morphology and DNA)~~. ~~A Based on the extant species only (ASDSF = 0.000521~~0.000647). BD~~~~ **A** †"*Gobius*" *francofurtanus* and †*G. jarosi* were added to the extant species (ASDSF = 0.014107~~0.005118~~). ~~C~~ **B** Both †*Eleogobius* species were added to the extant species (ASDSF = 0.004114~~0.005202~~). Scale bars, average number of substitutions per site respectively character changes per character.

~~Total-evidence phylogenies including~~ †*Pirskenius diatomaceus* and †*P. radoni*.

Formatted: Font: 12 pt

In the Bayesian tree, topologies are the same as described above; the two species of †*Pirskenius* are recovered as sister to Thalasseleotrididae (Fig 8~~7D~~A). However, in comparison to the Bayesian total-evidence phylogeny based on extant taxa only, ~~clearly~~ decreased support values occur for the 5brG clade (0.~~71~~89 vs. 1.00), the Thalasseleotrididae (0.~~84~~65 vs. 1.00), and for the gobiid clade of *Tigrigobius* + *Gobius niger* (0.~~77~~90 vs. 1.00). Support ~~values~~ for the clade Thalasseleotrididae plus one of the †*Pirskenius* species ~~are~~ is similar when only †*P. radoni* is added (0.69) (Fig S19), but higher when only †*P. diatomaceus* is ~~included~~ involved (0.9~~0~~1) (Fig S20). ~~, but low when only †*P. radoni* (0.54) or both species (0.60) are added (Fig 7D).~~

The single Maximum Parsimony ~~MP~~ tree including both species of †*Pirskenius* recovers all recent families as monophyletic (Fig S21) ~~. When both species or each species alone are only †*P. diatomaceus* areis included,~~ and the genus is recovered as sister to Thalasseleotrididae + 5brG; ~~(830% and 6973%, Figs S5A, B, C);~~ its monophyly is supported with 74~~65~~% BS ~~in the former case~~. Adding solely †*P. radoni* or †*P. diatomaceus* results in the same topology and similar support values (Figs. S22, S23) as seen in the tree including both species. ~~In contrast, when only †*P. radoni* is included, it is placed within Thalasseleotrididae (< 50%) (Fig S5B).~~

~~Total-evidence phylogenies including~~ †*Paralates bleicheri* and †*Pa. chapelcorneri*. –

Inclusion of both species of †*Paralates* does not recover a relationship of these two fossil taxa with any of the extant clades in the Bayesian phylogeny, rather they form a polytomy with the Odontobutidae + Rhyacichthyidae clade and the clade containing all other families (Fig 8~~C~~B). The Bayesian phylogeny that includes only †*Pa. bleicheri* resolves this species as sister to Odontobutidae (Fig ~~8~~S24~~A~~), albeit with relatively low support (0.60). In comparison with the

Formatted: Font: Italic

Formatted: Font: 12 pt

total-evidence phylogeny including only extant taxa, support for Odontobutidae decreased (0.61 vs. 1.00), while the high support for all other deeper nodes was not affected. The Bayesian phylogeny that contains only †*Pa. chapelcorneri* recovers this species ~~in~~ as sister to the clade containing the 5brG, Thalasseleotrididae, Butidae and Eleotridae~~remaining families~~, but ~~its~~ with very low support (0.52) ~~exact position was not resolved~~ (Fig ~~8~~S25~~B~~; note also decreased support for some backbone nodes in this tree).

Formatted: Indent: First line: 0"

In the Maximum Parsimony ~~MP~~ analyses, inclusion of both species could not resolve their phylogenetic position, as in the Bayesian tree, but here the resolution of the backbone of the tree is even more severely reduced (Fig ~~S6C~~S26). The Maximum Parsimony results for †*Pa. bleicheri* (Fig S27) match those of the Bayesian analyses, whereas when only †*Pa. chapelcorneri* is included, it is placed within Thalasseleotrididae (< 50%, Fig S~~6A, B~~28).

†*Pirskenius* spp. and †*Paralates* spp. – When all four †*Paralates* and †*Pirskenius* species were included in the Bayesian analysis, they were recovered in a †*Paralates* + †*Pirskenius* clade (0.74)~~2)~~, which was resolved as sister to Thalasseleotrididae ~~(albeit~~ with ~~low~~ moderate support ~~of (~~0.~~56~~79) (Fig S29~~8D~~).

In the Maximum Parsimony ~~MP~~ tree †*Pirskenius* is recovered monophyletic (64~~52~~%) but its position and the positions of the two †*Paralates* species within a clade together with Butidae, Thalasseleotrididae and 5brG are not resolved; overall, this tree shows very poor resolution ~~outside Thalasseleotrididae + 5brG~~at its backbone (Fig S~~7~~30).

Fig 8. 50% MRC Bayesian trees with posterior probabilities based on ~~a~~ the total-evidence ~~approach~~ dataset~~(morphology and DNA) using the extant species and adding one, two, or four fossil species~~. A †*Pirskenius diatomaceus* and †*P. radoni* were added to the extant species (ASDSF = 0.017673~~0.017558~~). ~~A~~ **B** †*Paralates bleicheri* and †*Pa.*

chapelcorneri was were added to the extant species (ASDSF = 0.0024600.001756). **B** †*Paralates chapelcorneri* was added to the extant species (ASDSF = 0.0083400.007668). **C** Both †*Paralates* species were added to the extant species (ASDSF = 0.0069950.004723). **D** The two species of †*Paralates* and the two species of †*Pirskenius* were added to the extant species (ASDSF = 0.0080770.010550). Scale bars, average number of substitutions per site respectively character changes per character.

Fig 7. 50% MRC Bayesian trees with posterior probabilities based on a total evidence approach (morphology and DNA) using the extant species and adding one or two fossil species. A †*Eleogobius brevis* was added to the extant species (ASDSF = 0.0013310.002136). **B** †*Eleogobius gaudanti* was added to the extant species (ASDSF = 0.0005690.000477).

Fig 9. 50% MRC Bayesian tree with posterior probabilities. Based on a total evidence approach (morphology and DNA) using the extant species and all ten fossil species included in this study (ASDSF = 0.009290). Scale bar, average number of substitutions per site.

Discussion

In this study, we have assembled for the first time a dataset comprising molecular and morphological data for Gobioidei that encompasses both extant and fossil species. This approach was necessary as a phylogenetic analysis of the extant species based solely on their morphological characters could only resolve those clades for which morphological apomorphies are known, i.e. 5brG, Thalasseleotrididae, Thalasseleotrididae+5brG, and Eleotridae [see 15, 28], while Butidae, Odontobutidae and Rhyacichthyidae each were recovered as paraphyletic. The overall objective was to investigate whether a fossil gobioid species can be confidently placed at family level in the tree of the extant Gobioidei using a Bayesian and or Maximum Parsimony total--evidence phylogenetic approach. [see 15, 28]

The results reveal mostly well supported placement at family level when a single fossil species is added to the total evidence data set of the extant species, especially ~~when~~ in the Bayesian ~~approach was used~~setting.

Five of the fossil species used here had previously been assigned at family level based on a comparative approach: †*Lepidocottus aries* had been placed within Butidae [44], †"*Gobius*" *francofurtanus* and †*G*~~.~~*obius jarosi* had been proposed as members of Gobiidae [46, 100], †*Pirskenius* spp. had been placed in its own family †Pirskeniidae [49] and a sister group relation of †Pirskeniidae to Thalasseleotrididae + 5brG has been suggested [11]. Each of those fossil taxa have been recovered in corresponding positions in the present study (Figs 6~~C~~B, 7A~~D~~, ~~7D~~8A). This implies that using comparative morphology has been a very appropriate method to classify those fossils. The family assignment of the remaining five fossil species analyzed here (†*Carlomonnius quasigobius*, †*Eleogobius* spp., †*Paralates* spp.) had been left as *incertae sedis* in previous work because they possess a mosaic set of characters that is not known among extant gobioids [42, 43, 64].

†*Carlomonnius quasigobius* originates from the Eocene of Monte Bolca in northern Italy [64], from the lower to middle Eocene (Ypresian to Lutetian, c. 50–40 Ma, see [101]). It is placed in Butidae in our study (Fig ~~6B~~6A), and it shares with some Butidae (especially with *Kribia*) a very small size, but any comparative approach would not have assigned †*C. quasigobius* to this family because it has only five branchiostegal rays (vs. six in Butidae) and a continuous dorsal fin (vs. divided). The feature that seems to have placed †*C. quasigobius* within the Butidae and close to *Kribia* is the number of 11 branched and segmented caudal fin rays, which is uncommon among other Gobioidei. Nevertheless, given that †*C. quasigobius* is the oldest gobioid species currently known [64], an assignment to the Gobiidae (with which it shares the number of five branchiostegal rays) seems~~is~~ unlikely and its classification within the Butidae appears to be~~seems~~ more plausible. It would expand the fossil record of Butidae from the early Oligocene (c. 30 Ma, [102]) to the early-middle Eocene (c. 50–40 Ma). ~~Of~~

~~course~~However, ~~one~~an additional possibility is that †*C. quasigobius* is a member of an extinct gobioid family or a "stem gobioid" showing a mixture of derived characters (five branchiostegal rays, dorsal postcleithrum absent, ~~13~~11 (7+6) ~~principal~~segmented and branched caudal-fin rays, four pelvic-fin rays) and plesiomorphic ones (dorsal fin continuous, 24 (10+14) vertebrae, autogenous haemal spine of the second preural centrum, first two abdominal centra shortened, first dorsal-fin pterygiophore inserting in the second interneural space) [64].

In case of †*Eleogobius* and †*Paralates*, the resulting phylogenies indicate that these genera are not monophyletic and their species may not even belong to the same family (see Figs~~.~~ 7B, 8B, S14–S18, S24–S28~~7A–C, 8A–C~~). The two species of †*Eleogobius* have been reported from the lower and middle Miocene (c. 17–14 Ma) of Central Europe, specifically southern Germany [42], Austria ([103], as *Gobius*), Switzerland [104, 105], and Croatia [106]. They have been interpreted as belonging to the same genus because they share a T-shaped palatine, absence of an endopterygoid and presence of six branchiostegal rays, and their otoliths are superficially similar, but show differences to recogni~~s~~ze the two species [42]. Of those characters, the T-shaped palatine and absence of an endopterygoid can be considered as apomorphic [23, 28], and would support assignment to Gobiidae, which is proposed, based on our phylogenetic results, for †*E. brevis* (Fig 7A). In contrast, the phylogenetic position of †*E. gaudanti* near Thalasseleotrididae (Fig 7B) is difficult to understand as no potential synapomorphies are known that can be recognized in a fossil. However, it is more or less consistent with the hypothesis of Gierl and Reichenbacher [42] that †*Eleogobius* is somewhat "in-between" the 5brG clade and the 6brG gobioids. ~~And~~Furthermore, ~~considering the work of~~ Bradić-Milinović et al. ~~(~~[41]~~)~~ have ~~who~~ recognized a difference in the arrangement of the branchiostegals in the two *Eleogobius* species, ~~, namely that †*E. gaudanti* has its two branchiostegals on the anteriormost part of the slender portion of the ceratohyal. This~~which is

Formatted: Font: Italic

a morphological hint thatappears to supports the possibility ofthat †*Eleogobius* is not being monophyletic.

In the case of †*Paralates*, a family assignment had not previously been proposed. †*Paralates bleicheri* has only been found in lower Oligocene deposits of the southern Upper Rhinegraben [40]. An assignment of †*P. bleicheri* to the Odontobutidae, as indicated in our analysis, receives some little indirect support based on its skeletal traits as all shared characters with the Odontobutidae represent plesiomorphic character states (e.g. seven spines in the first dorsal fin, 8–9 rays in the second dorsal fin, presence of postmaxillary process) (see Suppl. Table 1). from the occurrence of otoliths in the northern Upper Rhinegraben that have precisely the same age like the fossil skeletons of †*P. bleicheri* (Middle Pechelbronn Beds, Zone fossilifère à *Mytilus*, see [40, 107]). These otoliths have been described as „genus Percoideorum“ *validus* (Stinton 1977) [107]. However, they clearly resemble otoliths of the extant odontobutid species *Micropercops swinhonis* (figured in [108], pl. 315). As no other gobioid otoliths have yet been reported from the lower Oligocene deposits of the Upper Rhinegraben, one can assume that gobioids were solely represented by odontobutids at that time and in this region. However, fFinds of fossil skeletons of †*P. bleicheri* with otoliths preserved in situ are would be necessary to reinforce this hypothesis. †*Paralates chapelcorneri* originates from the upper Eocene “Chapelcorner Fish beds” of southern England (Isle of Wight) [38, 43]. No otoliths have yet been reported from the “Chapelcorner Fish beds”. The family assignment of this species remains a topic of future research based on new material of this species. Moreover, a possible relationship between †*Paralates* spp. and †*Pirskenius* spp. was indicated in the Bayesian and Maximum Parsimony trees shown in Figs 8D(Figs S25, S26) and 9, butwhich is possibly due to their specific combination of plesiomorphic (e.g. seven spines in the first dorsal fin, nine anal-fin rays) and apomorphic traits (e.g. 12 abdominal vertebrae, presence of interneural gap). , given the

~~autapomorphy of seven branchiostegal rays in †Pirskeniidae (vs. six in †*Paralates*) such a relationship can currently not be confirmed from a morphological viewpoint.~~

Our study also yields some new insights from a methodological point of view. Adding morphological data from only extant species to the molecular dataset had practically no influence on the tree topology and support values (Fig ~~6A~~S3). Likewise, the inclusion of a single fossil or of two congeneric species ~~of a monophyletic genus~~ did not change the tree topology, only sometimes some support values decreased (Figs 6~~B–D, 7,~~ 8). However, when all fossils were included in the total evidence phylogenetic framework, the resolution of relationships between families and most fossil taxa dramatically collapsed (Fig ~~9~~5B). Notably, the morphological phylogeny including the extant and all fossil species was less severe collapsed in the backbone of the tree as the 5brG clade and the Thalasseleotrididae were resolved (Fig 5A). It seems that in ~~this~~ the case of the total evidence phylogeny the fossil taxa added a high level of conflicting phylogenetic signals, which could not be overcome by the molecular data despite the latter having orders of magnitude more characters and harbouring strong signal for resolving gobioid phylogeny. A possible explanation is that ~~Of course,~~ the fossil taxa ~~add~~do ~~besides~~not only add morphological information, but also a lot of question marks to the ~~data set~~matrix, because, depending on their ~~not all fossils show a good~~ preservation, some morphological traits cannot be determined~~and so lots of features cannot be identified~~. An additional (or alternative) explanation is that many extinct gobioid clades and families, each with a unique character combination, existed in the past [11, 41, 45, 49]. These cannot be 'forced' in the tree of extant species and eventually may also be responsible for the collapse of the molecular backbone of the extant families. ~~Additionally, some very important phylogenetically informative features in Gobioidei (e.g. sensory papillae patterns; head canal and pore arrangements, e.g. see [71]) are just not visible in fossils.~~

Formatted: Highlight

This ~~remarkable result~~ highlights that increased sampling of fossil taxa in a total-evidence context is not universally beneficial, as might be expected, but strongly depends on the study group and peculiarities of the morphological data.

Formatted: Level 1, Space After: 0 pt, Line spacing: Double

Conclusion

We have presented a total-evidence dataset comprising molecular and morphological data of 29 extant gobioid species representing all families. Bayesian and Maximum Parsimony analyses revealed that this dataset is sufficient to achieve a molecular ‘backbone’ that fully conforms to previous molecular work. The new dataset can be used to analyze the family assignment of fossil skeletal-based gobioid species using Bayesian and Maximum Parsimony total-evidence phylogenetic approaches, which has not been possible before.

Our phylogenetic analyses confirmed the family assignment of those fossil gobioid species for which such a placement had been proposed in previous works. It is thus evident that comparative morphology remains an appropriate method to classify some gobioid fossils. However, our phylogenetic analyses could also ~~recover~~ suggest relationships of fossil gobioid species for cases where the comparative approach did not yield conclusive results. An example is †*Carlomonnius quasigobius*, which is the oldest gobioid fossil to date and our phylogeny suggests that it could be ~~recovered here as~~ a possible member of the Butidae, which would expand the known age of fossil butids from the early Oligocene (30 Ma) to the early-middle Eocene (40–50 Ma). Although such positioning of †*C. quasigobius* remains somewhat speculative for now, ~~This is of course far from certain but~~ it can give hints to look at certain fossil species from a different and new perspective.

We think that the total evidence framework presented here will be beneficial for all future work dealing with the phylogenetic placement of ~~a~~ fossil ~~skeleton-based~~ gobioids and

thus will help to improve our understanding of the evolutionary history of these fascinating fishes.

Acknowledgements

For providing technical assistance and access to specimens from the SNSB-ZSM collection we thank D. Neumann and U. Schliewen, respectively, (both SNSB-ZSM, Munich, Germany), with special thanks to the latter for help in the acquisition of specimens and insightful discussions. We are grateful to E. Bernard (NHMUK, London, UK), S. Merker (SMNS, Stuttgart, Germany), M. Parrent (MRAC, Tervuren, Belgium), and T. Přikryl (Charles University, Prague, Czech Republic), who all helped to study the specimens kept in the collections of their institutions. Sincere thanks go to W. Schwarzhans (Hamburg, Germany) for providing photographs of the otoliths of *Lesueurigobius sanzi*, *Asterropteryx semipunctata*, *Eucyclogobius newberry*, *Thalasseleotris iota* and and *Dormitator maculatus* (shown in Fig 3) and D. Nolf and K. Hoedemakers (both IRSNB, Brussels, Belgium) for supplying the otolith SEM image of *Odontobutis obscurus* (shown in Fig 3). For *Rhyacichthys* we thank the Vanuatu Environment Unit (Permit Numbers ENV326/001/1/07/DK and ENV326/001/1/08/D) and for *Protogobius* the New Caledonian South Province for allowing sampling (Permit Number 1224-08/PS). We thank G. Wörheide (LMU Munich, Germany) for providing access to computational resources. Finally we thank the reviewers and the Academic Editor of PLOS One for their constructive and valuable remarks, which greatly helped to improve the manuscript. We acknowledge funding for this project from the Deutsche Forschungsgemeinschaft to BR (RE 1113/20), and from the Ministry of Culture of the Czech Republic to RS (DKRVO 2019-2023/6.III.d National Museum, 00023272).

Author Contributions

Conceptualization: Christoph Gierl, Bettina Reichenbacher.

Data curation: Christoph Gierl, Bettina Reichenbacher.

Formal analysis: Martin Dohrmann, Christoph Gierl, Bettina Reichenbacher.

Funding acquisition: Bettina Reichenbacher, Radek Šanda.

Investigation: Bettina Reichenbacher, Christoph Gierl.

Methodology: Martin Dohrmann, Christoph Gierl, Bettina Reichenbacher.

Resources: Bettina Reichenbacher, Radek Šanda, Jasna Vukić, Philippe Keith, William Humphreys, Hamid R. Esmaeili.

Supervision: Bettina Reichenbacher.

Validation: Bettina Reichenbacher, Christoph Gierl, Martin Dohrmann.

Visualization: Bettina Reichenbacher, Martin Dohrmann, Christoph Gierl.

Writing – original draft: Christoph Gierl, Martin Dohrmann, Bettina Reichenbacher.

Writing – review & editing: Bettina Reichenbacher, Martin Dohrmann, Christoph Gierl, Philippe Keith, Radek Šanda, Jasna Vukić, Hamid R. Esmaeili, William Humphreys.

References

1. Nelson JS, Grande TC, Wilson MVH. Fishes of the World, Fifth Edition. Hoboken, New Jersey: John Wiley & Sons, inc.; 2016. 752 p.

2. Parenti P. A checklist of the gobioid fishes of the world (Percomorpha: Gobiiformes). Iran J Ichthyol. 2021;8:1–480. doi: 10.22034/iji.v8i0.556.

3. Brandl SJ, Goatley CHR, Bellwood DR, Tornabene L. The hidden half: ecology and evolution of cryptobenthic fishes on coral reefs. Biological Reviews. 2018;93(4):1846–73. Epub May 7. doi: 10.1111/brv.12423. PubMed PMID: WOS:000446427600008.

4. Patzner RA, Van Tassell JL, Kovačić M, Kapoor BG, editors. The biology of gobies. 1 ed. Enfield, New Hampshire: Science Publishers Inc.; 2011.

5. Tornabene L, Robertson DR, Baldwin CC. *Varicus lacerta*, a new species of goby (Teleostei, Gobiidae, Gobiosomatini, *Nes* subgroup) from a mesophotic reef in the southern Caribbean. ZooKeys. 2016;596:143–56. doi: 10.3897/zookeys.596.8217. PubMed PMID: WOS:000378055700010; PubMed Central PMCID: PMC4926659.

6. Jaafar Z, Murdy EO, editors. Fishes Out of Water: Biology and Ecology of Mudskippers. 1st ed. Boca Raton: Taylor & Francis; 2017.

7. Keith P. Biology and ecology of amphidromous Gobiidae of the Indo-Pacific and the Caribbean regions. J Fish Biol. 2003;63(4):831–47. Epub Sep 26. doi: 10.1046/j.1095-8649.2003.00197.x. PubMed PMID: WOS:000186047000001.

8. Karplus I, Szlep R, M T. Goby-shrimp partner specificity. I. Distribution in the northern Red Sea and partner specificity. J Exp Mar Biol Ecol. 1981;51(1):1–19. Epub Mar 31, 2003. doi: 10.1016/0022-0981(81)90151-9.

9. Karplus I, Thompson AR. The partnership between gobiid fishes and burrowing alpheid shrimps. In: Patzner RA, Van Tassell JL, Kovačić M, Kapoor BG, editors. The biology of gobies. Enfield, NH: Science Publishers Inc.; 2011. p. 559–607.

10. Thacker CE, Satoh TP, Katayama E, Harrington RC, Eytan RI, Near TJ. Molecular phylogeny of Percomorpha resolves *Trichonotus* as the sister lineage to Gobioidei (Teleostei: Gobiiformes) and confirms the polyphyly of Trachinoidei. Molecular phylogenetics and evolution. 2015;93:172–9. Epub Aug. doi: 10.1016/j.ympev.2015.08.001. PubMed PMID: MEDLINE:26265255; PubMed Central PMCID: PMC26265255.

11. Reichenbacher B, Přikryl T, Cerwenka AF, Keith P, Gierl C, Dohrmann M. Freshwater gobies 30 million years ago: New insights into character evolution and phylogenetic relationships of †Pirskeniidae (Gobioidei, Teleostei). PloS one. 2020;15(8):e0237366. Epub Aug 24. doi: 10.1371/journal.pone.0237366. PubMed PMID: WOS:000565550400035.

12. Agorreta A, San Mauro D, Schliewen U, Van Tassell JL, Kovačić M, Zardoya R, et al. Molecular phylogenetics of Gobioidei and phylogenetic placement of European gobies. Molecular phylogenetics and evolution. 2013;69(3):619–33. doi: 10.1016/j.ympev.2013.07.017. PubMed PMID: MEDLINE:23911892.

13. McCraney WT, Thacker CE, Alfaro ME. Supermatrix phylogeny resolves goby lineages and reveals unstable root of Gobiaria. Molecular phylogenetics and evolution. 2020;151:106862. Epub May 28. doi: 10.1016/j.ympev.2020.106862.

14. Akihito, Iwata A, Kobayashi T, Ikeo K, Imanishi T, Ono H, et al. Evolutionary aspects of gobioid fishes based upon a phylogenetic analysis of mitochondrial cytochrome *b* genes. Gene. 2000;259(1–2):5–15. doi: 10.1016/S0378-1119(00)00488-1. PubMed PMID: ISI:000166338300003.

15. Gill AC, Mooi RD. Thalasseleotrididae, new family of marine gobioid fishes from New Zealand and temperate Australia, with a revised definition of its sister taxon, the Gobiidae (Teleostei: Acanthomorpha). Zootaxa. 2012;3266(1):41–52. doi: 10.11646/zootaxa.3266.1.3.

16. Thacker CE. Biogeography of goby lineages (Gobiiformes: Gobioidei): origin, invasions and extinction throughout the Cenozoic. J Biogeogr. 2015;42(9):1615–25. Epub Jun 13. doi: 10.1111/jbi.12545. PubMed PMID: WOS:000359376600004.

17. McAllister DE. Evolution of the branchiostegals and classification of teleostome fishes. Bull natn Mus Can (Biol Ser). 1968;77:1–239.
18. Carpenter KE, Niem VH. The living marine resources of the Western Central Pacific. Volume 5. Bony fishes part 3 (Menidae to Pomacentridae). Rome: Food and Agriculture Organization of the United Nations (FAO); 2001. 2791–3380 p.
19. Fraser TH. Comparative osteology of the shallow water cardinal fishes (Perciformes: Apogonidae) with reference to the systematics and evolution of the family. Ichthyological Bulletin of the J L B Smith Institute of Ichthyology. 1972;34:1–105.
20. Regan CT. The osteology and classification of the gobioid fishes. The Annals and Magazine of Natural History [Eighth Series]. 1911;8(48):729–33.
21. Van Tassell JL, Tornabene L, Taylor MS. A history of gobioid morphological systematics. In: Patzner RA, Van Tassell JL, Kovačić M, Kapoor BG, editors. The biology of gobies. Enfield, NH: Science Publishers Inc.; 2011. p. 3–22.
22. Akihito. A systematic examination of the gobiid fishes based on the Mesopterygoid, Postcleithra, Branchiostegals, pelvic fins, Scapula, and Suborbital. Jpn J Ichthyol. 1969;16(3):93–104. Epub Jun 28, 2010. doi: 10.11369/jji1950.16.93. PubMed PMID: BCI:BCI197051058231.
23. Hoese DF. Gobioidei: relationships. In: Moser HG, Richards WJ, Cohen DM, Fahay MP, Kendall J, A. W., Richardson SL, editors. Ontogeny and systematics of fishes. Gainesville, Florida: American Society of Ichthyologists and Herpetologists; 1984. p. 588–91.
24. Miller PJ. The osteology and adaptive features of *Rhyacichthys aspro* (Teleostei: Gobioidei) and the classification of gobioid fishes. Journal of Zoology. 1973;171(3):397–434. doi: 10.1111/j.1469-7998.1973.tb05347.x.
25. Thacker CE, Hardman MA. Molecular phylogeny of basal gobioid fishes: Rhyacichthyidae, Odontobutidae, Xenisthmidae, Eleotridae (Teleostei: Perciformes: Gobioidei). Molecular phylogenetics and evolution. 2005;37(3):858–71. Epub 2005/06/25. doi: 10.1016/j.ympev.2005.05.004. PubMed PMID: 15975831.
26. Harrison IJ. Specialization of the gobioid palatopterygoquadrate complex and its relevance to gobioid systematics. Journal of Natural History. 1989;23(2):325–53. doi: 10.1080/00222938900770211.
27. Pezold F. Evidence for a monophyletic Gobiinae. Copeia. 1993;1993(3):634–43. doi: 10.2307/1447224. PubMed PMID: BCI:BCI199396109145.
28. Hoese DF, Gill AC. Phylogenetic relationships of eleotridid fishes (Perciformes, Gobioidei). B Mar Sci. 1993;52(1):415–40. PubMed PMID: ISI:A1993KX23000015.
29. Thacker CE. Phylogeny of Gobioidei and placement within Acanthomorpha, with a new classification and investigation of diversification and character evolution. Copeia. 2009;2009(1):93–104. doi: 10.1643/Ci-08-004. PubMed PMID: ISI:000263748000013.

30. Larson HK. A revision of the gobiid fish genus *Mugilogobius* (Teleostei: Gobioidei), and its systematic placement. Rec West Aust Mus. 2001;62:1–233. doi: 10.18195/issn.0313-122x.62.2001.001-233.

31. Murdy EO. A taxonomic revision and cladistic analysis of the oxudercine gobies (Gobiidae: Oxudercinae). Rec West Aust Mus Suppl. 1989;11:1–93. Epub Jun 16, 2009. doi: 10.3853/j.0812-7387.11.1989.93. PubMed PMID: BCI:BCI199089035472.

32. Harold AS, Winterbottom R, Munday PL, Chapman RW. Phylogenetic relationships of Indo-Pacific coral gobies of the genus *Gobiodon* (Teleostei: Gobiidae), based on morphological and molecular data. B Mar Sci. 2008;82(1):119–36. PubMed PMID: WOS:000252842600008.

33. Lord C, Bellec L, Dettaï A, Bonillo C, Keith P. Does your lip stick? Evolutionary aspects of the mouth morphology of the Indo-Pacific clinging goby of the *Sicyopterus* genus (Teleostei: Gobioidei: Sicydiinae) based on mitogenome phylogeny. Journal of Zoological Systematics and Evolutionary Research. 2019;57(4):910–25. Epub May 30. doi: 10.1111/jzs.12291.

34. Pezold F. Phylogenetic analysis of the genus *Gobionellus* (Teleostei: Gobiidae). Copeia. 2004;2004(2):260–80. doi: 10.1643/CI-02-218R3. PubMed PMID: WOS:000221336700006.

35. Tornabene L, Deis B, Erdmann MV. Evaluating the phylogenetic position of the goby genus *Kelloggella* (Teleostei: Gobiidae), with notes on osteology of the genus and description of a new species from Niue in the South Central Pacific Ocean. Zool J Linn Soc. 2018;183(1):143–62. Epub Dec 16, 2017. doi: 10.1093/zoolinnean/zlx060. PubMed PMID: WOS:000432303500006.

36. Tornabene L, Greenfield DW, Erdmann MV. A review of the *Eviota zebrina* complex, with descriptions of four new species (Teleostei, Gobiidae). ZooKeys. 2021;1057:149–84. doi: 10.3897/zookeys.1057.66675.

37. Gaudant J. Sur les conditions de gisement de l'ichthyofaune oligocène d'Aix-en-Provence (Bouches-du-Rhône): Essai de définition d'un modèle paléoécologique et paléogéographique. Géobios. 1978;11(3):393–7. doi: 10.1016/S0016-6995(78)80039-4.

38. Gaudant J, Quayle WJ. New palaeontological studies on the Chapelcorner fish bed (Upper Eocene, Isle of Wight). Bull Br Mus nat Hist (Geol). 1988;44(1):15–39.

39. Gaudant J. Nouvelles observations sur les poissons oligocènes de Monteviale (Vicenza - Italie). Mem Sci Geol. 1978;32:1–9.

40. Gaudant J. Sur la présence de Gobiidae (Poissons téléostéens) dans l'Oligocène inférieur de Rouffach (Haut-Rhin). Sci Géol Bull. 1979;32(3):131–7. doi: 10.3406/sgeol.1979.1560.

41. Bradić-Milinović K, Ahnelt H, Rundić L, Schwarzhans W. The lost freshwater goby fish fauna (Teleostei, Gobiidae) from the early Miocene of Klinci (Serbia). Swiss Journal of Palaeontology. 2019;138(2):285–315. Epub June 1. doi: 10.1007/s13358-019-00194-4.

42. Gierl C, Reichenbacher B. A new fossil genus of Gobiiformes from the Miocene characterized by a mosaic set of characters. Copeia. 2015;103(4):792–805. Epub Nov 12. doi: 10.1643/ci-14-146. PubMed PMID: WOS:000366679700006.

43. Gierl C, Reichenbacher B. Revision of so-called *Pomatoschistus* (Gobiiformes, Teleostei) from the late Eocene and early Oligocene. Palaeontologia Electronica. 2017;20.2.33A:1–17. doi: 10.26879/721. PubMed PMID: WOS:000405188500014.

44. Gierl C, Reichenbacher B, Gaudant J, Erpenbeck D, Pharisat A. An extraordinary gobioid fish fossil from southern France. PloS one. 2013;8(5):e64117. Epub May 15. doi: 10.1371/journal.pone.0064117. PubMed PMID: 23691158; PubMed Central PMCID: PMCPMC3655028.

45. Reichenbacher B, Bannikov AF. Diversity of gobioid fishes in the late middle Miocene of northern Moldova, Eastern Paratethys – part I: an extinct clade of *Lesueurigobius* look-alikes. PalZ. 2022;96:67–112. Epub Aug 26, 2021. doi: 10.1007/s12542-021-00573-8.

46. Reichenbacher B, Gregorová R, Holcová K, Šanda R, Vukić J, Přikryl T. Discovery of the oldest *Gobius* (Teleostei, Gobiiformes) from a marine ecosystem of Early Miocene age. Journal of Systematic Palaeontology. 2018;16(6):493–513. Epub May 2, 2017. doi: 10.1080/14772019.2017.1313323. PubMed PMID: WOS:000424810000002.

47. Schwarzhans W, Ahnelt H, Carnevale G, Japundžić S, Bradić K, Bratishko A. Otoliths in situ from Sarmatian (Middle Miocene) fishes of the Paratethys. Part III: tales from the cradle of the Ponto-Caspian gobies. Swiss Journal of Palaeontology. 2017;136(1):45–92. Epub Nov 10, 2016. doi: 10.1007/s13358-016-0120-7.

48. Gaudant J. Mise en évidence des plus anciens Gobioidei (Poissons téléostéens) connus dans le Lutétien inférieur marin de Catalogne (Espagne). Comptes rendus de l'Académie des sciences Série II, Sciences de la terre et des planètes. 1996;322(1):71–6. PubMed PMID: ISI:A1996TT86800009.

49. Obrhelová N. Vergleichende Osteologie der tertiären Süsswasserfische Böhmens (Gobioidei). Sbornik Paläont. 1961;26:103–92.

50. Schwarzhans W, Brzobohatý R, Radwańska U. Goby otoliths from the Badenian (middle Miocene) of the Central Paratethys from the Czech Republic, Slovakia and Poland: A baseline for the evolution of the European Gobiidae (Gobiiformes; Teleostei). Bollettino della Società Paleontologica Italiana. 2020;59(2):125–73. doi: 10.4435/bspi.2020.10. PubMed PMID: WOS:000562503100004.

51. Schwarzhans W, Agiadi K, Carnevale G. Late Miocene-Early Pliocene evolution of Mediterranean gobies and their environmental and biogeographic significance. Riv It Paleont Strat. 2020;126(3):657–724. PubMed PMID: WOS:000584561400003.

52. Keith P. Threatened fishes of the world: *Rhyacichthys guilberti* Dingerkus & Séret, 1992 (Rhyacichthyidae). Environ Biol Fish. 2002;63(1):40–. doi: 10.1023/a:1013827820971. PubMed PMID: WOS:000173466700006.

53. Lord C, Keith P. Threatened fishes of the world: *Protogobius attiti* Watson & Pöllabauer, 1998 (Rhyacichthyidae). Environ Biol Fish. 2006;77(1):101–2. doi: 10.1007/s10641-006-9060-1. PubMed PMID: WOS:000239741100010.

Formatted: German (Germany)

54. Akihito. Some morphological characters considered to be important in gobiid phylogeny. In: Matsuura K, editor. Indo-Pacific Fish Biology: Proceedings of the Second International Conference on Indo-Pacific Fishes. Tokyo: Ichthyological Society of Japan; 1986. p. 629–39.

55. Iwata A, Jeon S-R, Mizuno N, Choi K-C. A revision of the eleotrid goby genus *Odontobutis* in Japan, Korea and China. Jpn J Ichthyol. 1985;31(4):373–88. Epub Feb 23, 2011. doi: 10.11369/jji1950.31.373. PubMed PMID: WOS:A1985ADJ0200005.

56. Sparks JS, Chakrabarty P. Revision of the endemic Malagasy Cavefish genus *Typhleotris* (Teleostei: Gobiiformes: Milyeringidae) with discussion of its phylogenetic placement and description of a new species. American Museum Novitates. 2012;3764:1–28. doi: 10.1206/3764.2. PubMed PMID: WOS:000312236300001.

57. Hoese DF, Larson HK. New Australian fishes. Part 11. A new genus and species of eleotridid (Gobioidei) from Southern Australia with a discussion of relationships. Memoirs of the Museum of Victoria. 1987;48(1):43–50. PubMed PMID: BCI:BCI198987014125.

58. Schwarzhans W. Reconstruction of the Fossil Marine Bony Fish Fauna (Teleostei) from the Eocene to Pleistocene of New Zealand by Means of Otoliths: With Studies of Recent Congroid, Morid and Trachinoid Otoliths. Milano: Museo Civico di Storia Naturale di Milano; 2019. 390 p.

59. Hoese DF, Roberts CD. A new species of the eleotrid genus *Thalasseleotris* (Teleostei: Gobioidei) from New Zealand coastal waters. J Roy Soc New Zeal. 2005;35(4):417–31. doi: 10.1080/03014223.2005.9517793. PubMed PMID: ISI:000234492300004.

60. Birdsong RS, Murdy EO, Pezold FL. A study of the vertebral column and median fin osteology in gobioid fishes with comments on gobioid relationships. B Mar Sci. 1988;42(2):174–214. PubMed PMID: ISI:A1988N503000003.

61. Kindermann G, Miljković N, Ahnelt H, Stevenson DE. The osteology of *Eucyclogobius newberryi* and *Quietula guaymasiae* (Teleostei: Gobiidae), two closely related Gobionellines from the East Pacific. Annalen des Naturhistorischen Museums in Wien Serie B Botanik und Zoologie. 2007;108:13–56. PubMed PMID: BCI:BCI200700465922.

62. Gierl C. Articulated gobioid skeletons from the Frankfurt-Formation (Lower Miocene). München: Ludwig-Maximilians-Universität München; 2012.

63. Near TJ, Dornburg A, Eytan RI, Keck BP, Smith WL, Kuhn KL, et al. Phylogeny and tempo of diversification in the superradiation of spiny-rayed fishes. Proc Natl Acad Sci U S A. 2013;110(31):12738–43. doi: 10.1073/pnas.1304661110. PubMed PMID: 23858462; PubMed Central PMCID: PMC3732986.

64. Bannikov AF, Carnevale G. †*Carlomonnius quasigobius* gen. et sp. nov.: the first gobioid fish from the Eocene of Monte Bolca, Italy. Bulletin of Geosciences. 2016;91(1):13–22. Epub Dec 2, 2015. doi: 10.3140/bull.geosci.1577. PubMed PMID: WOS:000372546600002.

Formatted: German (Germany)

65. Gaudant J. Présence du genre *Lepidocottus* Sauvage, 1875 (Teleostei, Gobioidei) dans l'Oligocène inférieur des environs de Céreste (Alpes-de-Haute-Provence, France). Geodiversitas. 2015;37(2):229–35. doi: 10.5252/g2015n2a4. PubMed PMID: WOS:000357401300004.

66. Reichenbacher B, Gaudant J. On *Prolebias meyeri* (Agassiz) (Teleostei, Cyprinodontiformes) from the Oligo-Miocene of the Upper Rhinegraben area, with the establishment of a new genus and a new species. Eclogae Geologicae Helvetiae. 2003;96(3):509–20. doi: 10.1007/s00015-003-1098-x. PubMed PMID: WOS:000189178600014.

67. Přikryl T. A new species of the sleeper goby (Gobioidei, Eleotridae) from the České Středohoří Mountains (Czech Republic, Oligocene) and analysis of the validity of the family Pirskeniidae. Paläontol Z. 2014;88(2):187–96. Epub Jun 28, 2013. doi: 10.1007/s12542-013-0188-y.

68. La Mesa M, Arneri E, Caputo V, Iglesias M. The transparent goby, *Aphia minuta*: review of biology and fisheries of a paedomorphic European fish. Rev Fish Biol Fisheries. 2005;15(1–2):89–109. doi: 10.1007/s11160-005-1613-4. PubMed PMID: WOS:000233725400006.

69. Rojo AL. Osteología del chanquete, *Aphya minuta* (Risso, 1810) (Pisces: Gobiidae). Bol Inst Esp Oceanogr. 1985;2(1):165–79. PubMed PMID: BCI:BCI198681089950.

70. Van Tassell JL, Miller PJ, Brito A. A revision of *Vanneaugobius* (Teleostei: Gobiidae), with description of a new species. Journal of Natural History. 1988;22(2):545–67. Epub Feb 17, 2007. doi: 10.1080/00222938800770371.

71. Hoese DF, Fourmanoir P. *Discordipinna griessingeri*, a new genus and species of gobiid fish from tropical Indo-West Pacific. Jpn J Ichthyol. 1978;25(1):19–24. doi: 10.11369/jji1950.25.19. PubMed PMID: ISI:A1978FG43000003.

72. Esmaeili HR, Baghbani S, Zareian H, Shahryari F. Scale morphology of tank goby *Glossogobius giuris* (Hamilton-Buchanan, 1822) (Perciformes: Gobiidae) using scanning electron microscope. Journal of Biological Sciences. 2009;9(8):899–903. doi: 10.3923/jbs.2009.899.903.

73. McKay SI, Miller PJ. The affinities of European sand gobies (Teleostei: Gobiidae). Journal of Natural History. 1997;31(10):1457–82. doi: 10.1080/00222939700770791. PubMed PMID: ISI:A1997XZ44500001.

74. Miller PJ. Gobiidae. In: Whitehead PJP, Bauchot M-L, Hureau J-C, Nielsen J, Tortonese E, editors. Fishes of the north-eastern Atlantic and the Mediterranean (FNAM). III. Paris: UNESCO; 1986. p. 1019–85.

75. Randall JE, Hoese DF. Revision of the Indo-Pacific dartfishes, genus *Ptereleotris* (Perciformes: Gobioidei). Indo-Pacific Fishes. 1985;7:1–36.

76. Miller PJ. Affinities, origin and adaptive features of the Australian Desert Goby *Chlamydogobius eremius* (Zietz, 1896) (Teleostei: Gobiidae). Journal of Natural History. 1987;21(3):687–705. Epub Feb 17, 2007. doi: 10.1080/00222938700770391. PubMed PMID: WOS:A1987H259500009.

77. Mestermann K, Zander CD. Vergleichende osteologische Untersuchungen an *Pomatoschistus*-Arten (Gobioidei, Pisces). Zool Jb Anat. 1984;111:501–42.

78. Whitley GP. New fishes from Australia and New Zealand. Proceedings of the Royal Zoological Society of New South Wales. 1956;for the Year 1954–55:34–8.

79. McDowall RM. Descriptive and taxonomic notes on *Grahamichthys radiatus* (Valenciennes), Eleotridae. Trans Roy Soc NZ, Zool. 1965;7(2):51–6.

80. Schwarzhans W. Reconstruction of the fossil marine bony fish fauna (Teleostei) from the Eocene to Pleistocene of New Zealand by means of otoliths. Memorie della Società Italiana di Scienze Naturali e del Museo di Storia Naturale di Milano. 2019;46:1–326.

81. Wongrat P. Systematics, comparative anatomy, and phylogeny of eleotrine gobies (Teleostei: Gobioidei): University of Bristol; 1977.

82. Larson HK, Foster R, Humphreys WF, Stevens MI. A new species of the blind cave gudgeon *Milyeringa* (Pisces: Gobioidei, Eleotridae) from Barrow Island, Western Australia, with a redescription of *M. veritas* Whitley. Zootaxa. 2013;3616(2):135–50. doi: 10.11646/zootaxa.3616.2.3. PubMed PMID: WOS:000315021500003; PubMed Central PMCID: PMC24758799.

83. Iwata A, Sakai H. *Odontobutis hikimius* n. sp.: A new freshwater goby from Japan, with a key to species of the genus. Copeia. 2002;2002(1):104–10. doi: 10.1643/0045-8511(2002)002[0104:ohnsan]2.0.co;2. PubMed PMID: BCI:BCI200200185878.

84. Shibukawa K, Iwata A, Viravong S. *Terateleotris*, a new gobioid fish genus from the Laos (Teleostei, Perciformes), with comments on its relationships. Bulletin of the National Science Museum Series A (Zoology). 2001;27(4):229–57. PubMed PMID: BCI:BCI200200292381.

85. Bergman LMR. The cephalic lateralis system of cardinalfishes (Perciformes: Apogonidae) and its application to the taxonomy and systematics of the family: University of Hawaii at Manoa; 2004.

86. McAllister DE. Mandibular pore pattern in the sculpin family Cottidae. Nat Mus Can Bull. 1968;223:58–69. PubMed PMID: BCI:BCI19684900097393.

87. Maddison WP, Maddison DR. Mesquite: a modular system for evolutionary analysis. Version 3.61 https://www.mesquiteproject.org. 3.61 ed2019.

88. Larsson A. AliView: a fast and lightweight alignment viewer and editor for large datasets. Bioinformatics. 2014;30(22):3276–8. Epub Aug 5. doi: 10.1093/bioinformatics/btu531. PubMed PMID: WOS:000344774600022; PubMed Central PMCID: PMCPMC4221126.

89. Edgar RC. MUSCLE: multiple sequence alignment with high accuracy and high throughput. Nucleic Acids Research. 2004;32(5):1792–7. doi: 10.1093/nar/gkh340. PubMed PMID: WOS:000220487200025; PubMed Central PMCID: PMCPMC390337.

90. Gouy M, Guindon S, Gascuel O. SeaView version 4: A multiplatform graphical user interface for sequence alignment and phylogenetic tree building. Molecular biology and evolution. 2010;27(2):221–4. doi: 10.1093/molbev/msp259. PubMed PMID: WOS:000273704400003.

Formatted: German (Germany)

91. Ronquist F, Teslenko M, van der Mark P, Ayres DL, Darling A, Höhna S, et al. MrBayes 3.2: efficient Bayesian phylogenetic inference and model choice across a large model space. Systematic Biology. 2012;61(3):539–42. doi: 10.1093/sysbio/sys029. PubMed PMID: WOS:000303336200013; PubMed Central PMCID: PMC3329765.

92. Goloboff PA, Farris JS, Nixon KC. TNT, a free program for phylogenetic analysis. Cladistics. 2008;24(5):774–86. doi: 10.1111/J.1096-0031.2008.00217.X. PubMed PMID: WOS:000259611200009.

93. Goloboff PA, Catalano SA. TNT version 1.5, including a full implementation of phylogenetic morphometrics. Cladistics. 2016;32(3):221–38. Epub Apr 25. doi: 10.1111/cla.12160. PubMed PMID: WOS:000376269000001.

94. Lewis PO. A likelihood approach to estimating phylogeny from discrete morphological character data. Systematic Biology. 2001;50(6):913–25. doi: 10.1080/106351501753462876.

95. Yang Z. Maximum likelihood phylogenetic estimation from DNA sequences with variable rates over sites: Approximate methods. Journal of Molecular Evolution. 1994;39(3):306–14. Epub Sep 1. doi: 10.1007/bf00160154. PubMed PMID: 7932792.

96. Lanave C, Preparata G, Saccone C, Serio G. A new method for calculating evolutionary substitution rates. Journal of Molecular Evolution. 1984;20(1):86–93. doi: 10.1007/bf02101990. PubMed PMID: WOS:A1984SN04000011.

97. Rambaut A, Drummond AJ, Xie D, Baele G, Suchard MA. Posterior summarization in Bayesian phylogenetics using Tracer 1.7. Systematic Biology. 2018;67(5):901–4. Epub 2018/05/03. doi: 10.1093/sysbio/syy032. PubMed PMID: WOS:000443580600012; PubMed Central PMCID: PMCPMC6101584.

98. Felsenstein J. Confidence Limits on Phylogenies: An Approach Using the Bootstrap. Evolution. 1985;39(4):783–91. doi: 10.2307/2408678. PubMed PMID: WOS:A1985APJ8100007.

99. Rambaut A. FigTree. Tree Figure Drawing Tool version 1.4.4. http://tree.bio.ed.ac.uk/software/figtree/. 1.4.4 ed. Edinburgh: University of Edinburgh; 2018.

100. Weiler W. Die Fischfauna des Tertiärs im oberrheinischen Graben, des Mainzer Beckens, des unteren Maintals und der Wetterau, unter besonderer Berücksichtigung des Untermiozäns. Abhandlungen der Senckenbergischen Naturforschenden Gesellschaft. 1963;504:1–75.

101. Marramà G, Bannikov AF, Tyler JC, Zorzin R, Carnevale G. Controlled excavations in the Pesciara and Monte Postale sites provide new insights about the palaeoecology and taphonomy of the fish assemblages of the Eocene Bolca Konservat-Lagerstätte, Italy. Palaeogeography, Palaeoclimatology, Palaeoecology. 2016;454:228-45.

102. Pandolfi L, Carnevale G, Costeur L, Del Favero L, Fornasiero M, Ghezzo E, et al. Reassessing the earliest Oligocene vertebrate assemblage of Monteviale (Vicenza, Italy). Journal of Systematic Palaeontology. 2017;15(2):83–127. Epub Mar 16, 2016. doi: 10.1080/14772019.2016.1147170.

Formatted: German (Germany)

103. Brzobohatý R, Gaudant J. *Gobius brevis* (Agassiz, 1839), a gobiid fish with otoliths in situ (Pisces, Teleostei) in the Karpatian (Lower Miocene) of the Vienna Basin. Annalen des Naturhistorischen Museums in Wien - Serie A (Mineralogie und Petrographie, Geologie und Paläontologie, Archäozoologie, Anthropologie und Prähistorie). 2009;111:245–55. PubMed PMID: BCI:BCI200900349722.

104. Jost J, Kälin D, Börner S, Vasilyan D, Lawver D, Reichenbacher B. Vertebrate microfossils from the Upper Freshwater Molasse in the Swiss Molasse Basin: Implications for the evolution of the North Alpine Foreland Basin during the Miocene Climate Optimum. Palaeogeography, Palaeoclimatology, Palaeoecology. 2015;426:22–33. doi: 10.1016/j.palaeo.2015.02.028. PubMed PMID: WOS:000353603000003.

105. Jost J, Kälin D, Schulz-Mirbach T, Reichenbacher B. Late Early Miocene lake deposits near Mauensee, central Switzerland: fish fauna (otoliths, teeth), accompanying biota and palaeoecology. Eclogae Geologicae Helvetiae. 2006;99(3):309–26. Epub Jan 12, 2007. doi: 10.1007/s00015-006-1198-5.

106. Mandic O, Hajek-Tadesse V, Bakrač K, Reichenbacher B, Grizelj A, Miknić M. Multiproxy reconstruction of the middle Miocene Požega palaeolake in the Southern Pannonian Basin (NE Croatia) prior to the Badenian transgression of the Central Paratethys Sea. Palaeogeography, Palaeoclimatology, Palaeoecology. 2019;516:203–19. Epub Dec 7, 2018. doi: 10.1016/j.palaeo.2018.12.003. PubMed PMID: WOS:000456903000018.

Appendix 1

Short description of the 10 fossil specimens included in this study.

Additional Information

All data matrices and trees are available on figshare (https://figshare.com/s/ed10b9a5ac382f856a20).

Supporting information

Suppl. Table. Part A. Distribution of character states of the characters 1–54 among the examined 29 extant and 10 fossil gobioid species. **Part B.** List of characters, their states and literatur sources. Colour indicates how the character state was determined; ? indicates that character state is not known.

Captions for Figures S1–S30

Fig S1 Maximum Parsimony tree with bootstrap values based on published DNA data of the 29 gobioid species used in this study (single tree retained; for sources of molecular data see Table 1). TL = 10112, CI = 0.407, RI = 0.377.

Fig S2 Maximum Parsimony tree with bootstrap values based on morphological data of the extant species only (single tree retained). TL = 179, CI = 0.559, RI = 0.782.

Fig S3 50% MRC Bayesian tree with posterior probabilities based on a total evidence approach (morphology and DNA) based on the extant species only (ASDSF = 0.000521).

Fig S4 Maximum Parsimony tree with bootstrap values based on a total evidence approach (morphology and DNA) of the extant species only (single tree retained). TL = 10312, CI = 0.409, RI = 0.390.

Fig S5 Maximum Parsimony tree with bootstrap values based on morphological data (50% majority rule consensus of six trees) of the extant species plus ten fossil species (indicated with red frames). TL = 227, CI = 0.467, RI = 0.715.

Fig S6 Maximum Parsimony tree with bootstrap values based on a total evidence approach (morphology and DNA) of the extant species plus all ten fossil species included in this study (50% majority rule consensus of two trees). TL = 10633, CI = 0.397, RI = 0.362.

Fig S7 Maximum Parsimony tree with bootstrap values based on a total evidence approach (morphology and DNA) of the extant species plus †*Carlomonnius quasigobius* (single tree retained). TL = 10321, CI = 0.409, RI = 0.390.

Fig S8 Maximum Parsimony tree with bootstrap values based on a total evidence approach (morphology and DNA) of the extant species plus †*Lepidocottus aries* (single tree retained). TL = 10316, CI = 0.409, RI = 0.390.

Fig S9 Maximum Parsimony tree with bootstrap values based on a total evidence approach (morphology and DNA) of the extant species plus †"*Gobius*" *francofurtanus* and †*Gobius jarosi* (50% majority rule consensus tree of two trees). TL = 10623, CI = 0.397, RI = 0.359.

Fig S10 50% MRC Bayesian tree with posterior probabilities based on a total evidence approach (morphology and DNA) using the extant species and adding †*Gobius francofurtanus* (ASDSF = 0.005129).

Fig S11 50% MRC Bayesian tree with posterior probabilities based on a total evidence approach (morphology and DNA) using the extant species and adding †*Gobius jarosi* (ASDSF = 0.002010).

Fig S12 Maximum Parsimony tree with bootstrap values based on a total evidence approach (morphology and DNA) of the extant species plus †*Gobius jarosi* (single tree retained). TL = 10313, CI = 0.409, RI = 0.390.

Fig S13 Maximum Parsimony tree with bootstrap values based on a total evidence approach (morphology and DNA) of the extant species plus †*Gobius francofurtanus* (single tree retained). TL = 10312, CI = 0.409, RI = 0.390.

Fig S14 50% MRC Bayesian tree with posterior probabilities based on a total evidence approach (morphology and DNA) using the extant species and †*Eleogobius brevis* (ASDSF = 0.001331).

Fig S15 50% MRC Bayesian tree with posterior probabilities based on a total evidence approach (morphology and DNA) using the extant species and †*Eleogobius gaudanti* (ASDSF = 0.000569).

Fig S16 Maximum Parsimony tree with bootstrap values based on a total evidence approach (morphology and DNA) of the extant species plus †*Eleogobius brevis* (single tree retained). TL = 10314, CI = 0.409, RI = 0.390.

Fig S17 Maximum Parsimony tree with bootstrap values based on a total evidence approach (morphology and DNA) of the extant species plus †*Eleogobius gaudanti* (single tree retained). TL = 10315, CI = 0.409, RI = 0.390.

Fig S18 Maximum Parsimony tree with bootstrap values based on a total evidence approach (morphology and DNA) of the extant species plus both †*Eleogobius* species (single tree retained). TL = 10317, CI = 0.409, RI = 0.390.

Fig S19 50% MRC Bayesian tree with posterior probabilities based on a total evidence approach (morphology and DNA) using the extant species and †*Pirskenius radoni* (ASDSF = 0.006008).

Fig S20 50% MRC Bayesian tree with posterior probabilities based on a total evidence approach (morphology and DNA) using the extant species and †*Pirskenius diatomaceus* (ASDSF = 0.003438).

Fig S21 Maximum Parsimony tree with bootstrap values based on a total evidence approach (morphology and DNA) of the extant species plus both †*Pirskenius* species (single tree retained). TL = 10324, CI = 0.409, RI = 0.390.

Fig S22 Maximum Parsimony tree with bootstrap values based on a total evidence approach (morphology and DNA) of the extant species plus †*Pirskenius radoni* (single tree retained). TL = 10320, CI = 0.409, RI = 0.390.

Fig S23 Maximum Parsimony tree with bootstrap values based on a total evidence approach (morphology and DNA) of the extant species plus †*Pirskenius diatomaceus* (single tree retained). TL = 10318, CI = 0.409, RI = 0.390.

Fig S24 50% MRC Bayesian tree with posterior probabilities based on a total evidence approach (morphology and DNA) using the extant species and adding †*Paralates bleicheri* (ASDSF = 0.002460).

Fig S25 50% MRC Bayesian tree with posterior probabilities based on a total evidence approach (morphology and DNA) using the extant species and adding †*Paralates chapelcorneri* (ASDSF = 0.008340).

Fig S26 Maximum Parsimony tree with bootstrap values based on a total evidence approach (morphology and DNA) of the extant species plus both †*Paralates* species (50% majority rule consensus of two trees). TL = 10824, CI = 0.390, RI = 0.339.

Fig S27 Maximum Parsimony tree with bootstrap values based on a total evidence approach (morphology and DNA) of the extant species plus †*Paralates bleicheri* (single tree retained). TL = 10313, CI = 0.409, RI = 0.390.

Fig S28 Maximum Parsimony tree with bootstrap values based on a total evidence approach (morphology and DNA) of the extant species plus †*Paralates chapelcorneri* (single tree retained). TL = 10316, CI = 0.409, RI = 0.390.

Fig S29 50% MRC Bayesian tree with posterior probabilities based on a total evidence approach (morphology and DNA) using the extant species and adding the two species of †*Paralates* and the two species of †*Pirskenius* (ASDSF = 0.008077). Scale bars, average number of substitutions per site.

Fig S30 Maximum Parsimony tree with bootstrap values based on a total evidence approach (morphology and DNA) of the extant species plus the species of †*Pirskenius* and †*Paralates* (50% majority rule consensus of seven trees). TL = 10333, CI = 0.408, RI = 0.389.

Reviewer 1:

Reviewer #1: Dear authors, This is an interesting article with a novel approach and incorporating otolith research for the first time in a total evidence phylogenetic analysis. My congratulations to your work.

There are, however, a few issues that need to be addressed in my view before this article can be published. I have made some comments in the main text and the supplementary data as attached, but there are a few principle issues which I would like to bring to your attention as follows:

1. I am lacking a discussion about the state of the characters used, i.e., plesiomorphic or apomorphic and polarity. This is particularly so for the otolith characters while for the other characters possibly references could be cited. In general, however, this is my most concerning issue.

We have added the requested information to the Suppl Table Part B by providing a new column in which we explain how the plesiomorphic character state was determined. If such information was available from the literature, we indicate the corresponding literature source in this column. If no such information was available from previous work, we have used the outgroup and for some characters additionally *Rhyacichthys* to define it. Each plesiomorphic character state is marked by a grey shading in Part B of the Suppl Table. We have complemented the Method part of the revised manuscript accordingly.

2. Did the phylogenetic analyses reflect plesiomorphic versus apomorphic character states and if so, how?

This can best be assessed using the tree based only on the morphological data of the extant species (Fig 4B). The clades for which apomorphies are known (5brG, Thalasseleotrididae, Thalasseleotrididae+5brG, Eleotridae) are resolved in this tree. Groups of which apomorphies are yet unknown are recovered as paraphyletic (Butidae, Odontobutidae, Rhyacichthyidae). We have added a notice about this in the first paragraph of the Discussion.

3. The phylogeny collapsed when all fossil taxa where included. I find this intriguing and the discussion to this aspect way too short, while the technical phylogenetic discussion from the various runs is rather exhaustive. Definitely, this aspect requires more attention and consideration and discussion than you provided in the manuscript.

We added some additional thoughts in the last paragraph of the discussion.

4. Much reference is made to the earliest gobioid on the fossil record- Carlomonnius quasigobius. A very detailed anatomical and phylogenetic analysis was provided by Bannikov & Carnevale (2016) who described it resulting in not placing it in a specific family because of its unique mixture of plesiomorphic and apomorphine characters. In this article it is now based in Butidae based on your phylogenetic modeling alone without discussion of how the results of Bannikov & Carnevale would relate. This needs to be considered and discussed in more detail because of its phylogenetic impact.

We added some additional thoughts in the discussion. We also explain that we cannot exclude that C. quasigobius represents an extinct clade with a combination of plesiomorphic (e.g. continuous

dorsal fin, 24 (10+14) vertebrae, autogenous haemal spine of the second preural centrum, first two abdominal centra shortened, first dorsal- fin pterygiophore inserting in the second interneural space) and derived characters (five branchiostegal rays, dorsal postcleithrum absent, 11 branched and segmented caudal-fin rays, four pelvic-fin rays], as described in Bannikov & Carnevale (2016).

With these aspects being carefully addressed I am of the opinion that the manuscript will become a very valuable article both in terms of the methodology applied as well as the results presented.

Thank you for the positive feedback and constructive comments. All of which have been considered in the revised version of this manuscript.

From Manuscript:

L.23: Here and later why not use the official abbreviation Ma?

"Ma" is established in the geoscientific community, while the colleagues of the biological community are not always familiar with this abbreviation. Therefore, we prefer to keep "m.y. ago"

L.33: This is still a relatively low coverage given that there are 2310 extant species and 320 extant genera.

We are aware of this issue (is also explicitly mentioned in the method part). However, our data set was sufficient to achieve a molecular 'backbone' that fully conforms to previous molecular work (please see following sentence of the Abstract, line 36, 37).

Would it make sense to refer to comparative publications?

We used comparative publications (see Table 1), which is indicated at the beginning of the sentence ("using both literature and newly collected data"). It is explained more in detail in the Material section.

L.46: Any rational why this should be so? There is some discussion at the end of the article, but in the abstract it should either be explained or left out.

We agree that in the abstract we can leave it out.

L.58: this is awful! Please write "sensu Thacker et al., 2015"

Ok, we changed to Thacker et al. [10]

L.59: The placement of Trichonotidae in that clade is controversial

Yes, because there are different definitions of the term "Gobiiformes", which is indicated in our Figure 1 and also mentioned in the text. In the sentence on line 59, we refer to the "Gobiiformes

sensu Thacker et al. (2015)" and according to Thacker et al. (2015), the Trichonotidae are part of the Gobiiformes.

L. 73: This is not uniformly accepted and I suggest to exclude them here.

As explained above, Trichonotidae are listed here according to the phylogeny of Thacker et al. 2015.

L.78: this would then not be necessary to highlight.

OK, done. In addition, we revised Figure 1 to include the information how Nelson et al. (2016) define Gobiiformes

Table: Gobius francofurtanus like does not belong to the genus Gobius, but is a relict from a time when Gobius was used as a junk basket for such fossil taxa. I would therefore recommend here and later to name it: "Gobius" francofurtanus.

OK, done.

L.189: don't understand the difference in these three terms. Please consider rewording. The explanatory figure shows only rectangular and rectangular/higher than long. How about simplier and more easy to understand terms such as long rectangular, high rectangular, quadratic?

OK, done.

L.191: this is not very informative. Please describe more precisely.

We changed it to perciform-like.

L.210: I am missing here or in the supplementary table a discussion about which character states are considered plesiomorphic and which apomorphic, i.e., the polarity of characters. In most cases, this can probably be resolved with referencing published articles, but not for otoliths, which is newly introduced here. So at least for those characters some detail discussion of character state and polarity is required.

We have added the plesiomorphic character states (and an explanation or reference) for each character to the supplementary Table 1. See above (point 1.)

L.602: but why is it not working when adding several fossil taxa? See discussion at end of article and comment to that.

We added some thoughts.

L.623: But how about Carlomonnius representing a fossil family like Pirskenius, or a stem Gobioidei. I am lacking a more specific discussion of how the assignment cope with the 5 derived and 5 plesimorphic characters discussed in Bannikov & Carnevale 2016.

OK, done, see above (point 4.)

L.630: these are universal characters of no specific phylogenetic value at the genus level.

True, that's where the otoliths come in... and it is clearer now in the text.

L.631: see Bradic-Milinovic et al 2019: it is not only the number of branchiostegal rays that may matter but also where they are inserted. Is this comparable among the two recorded species of Eleogobius or not?

This is a good point. Actually, the positions of the branchiostegal rays appear different in the two *Eleogobius* species. However, the available fossils are moderately well preserved, thus it is currently difficult to say whether the observable bone arrangement corresponds to the natural anatomical configuration. Nevertheless, it could be a hint, that the two *Eleogobius* species may actually represent two different genera. It is added to the text.

L.645: The figure of Micropercops swinhonis in Nolf 2013 is a drawing and not very detailed. Schwarzhans et al., 2017 contain photographs. I can see no clear resemblance of Umbra valida with Micropercops. I suggest to reword this sentance in a more careful manner. I further suggest to make reference to the late Oligocene Micropercops pomahaka in Schwarzhans et al. 2017 from New Zealand.

We have deleted the comparison to these otoliths from the manuscript as further evidence should be awaited

L.645: Stinton 1977 described it as Umbra valida and I recommend to refer to the original reference.

Not necessary any more as the corresponding text has been removed

L.665: Could this not rather be an indication that some fossil taxa do not fit into the backbone built from extant taxa because they represent extinct clades or stem taxa?

Yes. We have added this (last chapter of Discussion).

From Suppl Table-WWS:

In none of the listed 54 characters has it been noted and explained, which character state is considered plesimorphic and which apomorphic. For the majority of characters, this can possibly be deduced from published assessment. For otoliths this is new and character polarity should be discussed.

See above point 1.

From Supplementary Figures S1-S8-WWS:

Please note that the otolith of Thalasseleotris iota looks quite different from the ones figured in Schwarzhans, 2019, which includes information from a paratype.

We checked that and we found out that there has been an unfortunate error with the Thalasseleotris specimen we had received from a museum collection. This specimen was wrongly labelled and it was not Thalasseleotris, which we had not realized before. Now we have removed all data of this falsely labelled specimen (including its otolith) from our data and replaced all information by literature data (see Table 1).

From Appendix:

Not quite. Bannikov and Carnevale listed five derived and five plesiomorphic characters in a unique combination not found in any extant gobioid lineage. This should be adequately acknowledged here and correctly cited in the description and discussion thereof. Furthermore, it should be explained how the new familial allocation proposed from the ‚total evidence' aproach deals with the individual characters and assessment by Bannikov & Carnevale. Finally, it should be mentioned that this taxon represents the first/earliest confirmed record of a gobioid.

Done.

There is more to be considered than just the number. See Bradic-Milinovic et al. 2019 why the 6 branchiostegals in the case of Elegobius may not be homologous with the 6 branchiostegals found in Eleotridae, Butidae etc.

Done.

Reviewer 2:

Reviewer #2: This paper is well-written and I appreciate the hard work done by the authors. However, several questions and concerns arose during the readings. The authors claimed that they were able to assign several of the incertae sedis fossils to family level using their total-evidence phylogeny, but in many cases the results are not solid. Also, I was not able to review the Suppl. Figs 1-8 (what I was able to download was a docx. with the figures in the main text, not Suppl. Figs 1-8), so basically, I was only able to access half of the results presented.

1. The step-wise approach (L. 229) seems to have good resolution on the results, but what's the rationale here to this approach only? Because when multiple fossils are included in the analysis, the phylogeny collapsed (as in L. 664), which can be easily challenged by future works when more evidence and specimens are available, then, the total evidence phylogeny produced here is not a stable one, and means that you need other robust phylogeny (topology) for the ten fossil taxa (and perhaps not yours). Or, on the contrary, the morphological data are too few to reconstruct a good tree for fossils, leaving those inc. sed. still incertae sedis. If that's so, then you are forcing the taxonomic assignment to any of the extant families while they might well belong to any of an extinct clade.

We have restructured the Result part in the revised version to better explain the rationale of our approach. Yes, more morphological data could certainly help. However, the idea of our study was also that it could serve as a (promising) starting point to carry this work on. Not to mention the hundreds of extant goby species that have more or less unknown osteology.

Yes, the fossils may represent fossil clades/families and we have added this in the revised version of the manuscript (last chapter of Discussion).

2. L. 618, you mentioned these morphological traits, but these are conflicting your assignment below! Therefore, the statement in L. 615-624 is weak, there should be morphological evidence supporting your assignment rather than your phylogenetic tree (L. 583, with low support value!) or just stating the stratigraphic occurrences. Also, if C. quasigobius really is a Butidae, then the expansion of the fossil record is also a big deal (some 10-20 Ma earlier).

Done. We have rephrased the paragraph dealing with *Carlomonnius* in the revised version of the manuscript according to these concerns.

3. In my opinion, we only have a limited number of extant goby families and that might not be ideal to place the unknown, yet to be fully discovered fossil taxa to the living scheme, as there are myriads of unknown diversity behind (as you wrote in L. 611-614). Therefore, it might not be so surprising that the results are not well resolved as all.

Yes. We have added this (last chapter of Discussion).

4. L. 218, you mentioned some molecular data came from “Thacker et al. [62]”, but it didn’t match the ref. 62 in the References.

Corrected.

5. Suppl. Table. I suggest following Sereno (2007) for character description. Sereno, C. P. 2007. Logical basis for morphological characters in phylogenetics. Cladistics 23(6): 565-587.

We did not follow this suggestion as we do not consider it necessarily an Improvement

To Editor:

1. Please ensure that your manuscript meets PLOS ONE's style requirements, including those for file naming.

2. To comply with PLOS ONE submissions requirements, please provide methods of sacrifice in the Methods section of your manuscript.

We added that all specimens originate from museum collections (as indicated in Table 1) and no specimens were sacrificed for this work.

3. In your manuscript, please provide additional information regarding the specimens used in your study. Ensure that you have reported specimen numbers and complete repository information, including museum name and geographic location.

The requested information is provided in Table 1

4. We note that you have provided funding information that is not currently declared in your Funding Statement. However, funding information should not appear in the Acknowledgments section or other areas of your manuscript. We will only publish funding information present in the Funding Statement section of the online submission form. Please remove any funding-related text from the manuscript and let us know how you would like to update your Funding Statement.

Done

5. In your Data Availability statement, you have not specified where the minimal data set underlying the results described in your manuscript can be found. PLOS defines a study's minimal data set as the underlying data used to reach the conclusions drawn in the manuscript and any additional data required to replicate the reported study findings in their entirety. All PLOS journals require that the minimal data set be made fully available. For more information about our data policy, please see http://journals.plos.org/plosone/s/data-availability.

Upon re-submitting your revised manuscript, please upload your study's minimal underlying data set as either Supporting Information files or to a stable, public repository and include the relevant URLs, DOIs, or accession numbers within your revised cover letter.

Done

All data matrices and trees are available on figshare (https://figshare.com/s/ed10b9a5ac382f856a20).

Data Review URL

Click here to download Data Review URL
http://figshare.com/s/ed10b9a5ac382f856a20
