# Supplementary material for: An integrative phylogenetic approach for inferring relationships of fossil gobioids (Teleostei: Gobiiformes)
Source: PLoS One. 2022 Jul 8;17(7):e0271121. doi: 10.1371/journal.pone.0271121 (PMC9269936; doi:10.1371/journal.pone.0271121)
Supplement: Attachment — Submitted filename: Supplementary Figures S1-S8-WWS.docx [file pone.0271121.s006.docx]

**Fig 1. Current understanding of gobioid relationships showing the different nomenclatures in use.** Note that Gobiiformes/Gobiaria is referred to as Gobiida in Nelson et al. (2016), but the Trichonotidae are not included in their Gobiida. Thus, the Gobiiformes sensu Nelson et al. (2016) corresponds to the Gobioidei of Thacker et al. (2015) and Betancur-R et al. (2017). brG = branchiostegals.

**Fig 2.** Otoliths (left sagitta in each case) of all the species used in this study for which they were obtainable. The Figure shows specimens representing all gobioid families and an apogonid. Scale bars: 0.5 mm.

**Fig 3.** 50% MRC Bayesian tree with posterior probabilities (average standard deviation of split frequencies between two independent runs [ASDSF] = 0.000647) based on published DNA data of the 29 gobioid species used in this study (for sources of molecular data see Table 1). Scale bar, average number of substitutions per site.

**Fig 4. 50% MRC Bayesian trees with posterior probabilities based on morphological characters.** **A** Tree based on the extant species only (ASDSF = 0.002449). **B** Tree based on the extant species plus ten fossil species (indicated with †; ASDSF = 0.004479). Scale bars, average number of character changes per character.

**Fig 5. 50% MRC Bayesian trees with posterior probabilities based on a total evidence approach (morphology and DNA).** **A** Based on the extant species only (ASDSF = 0.000647). **B** †*Carlomonnius quasigobius* was added to the extant species (ASDSF = 0.006397). **C** †*Lepidocottus aries* was added to the extant species (ASDSF = 0.000888). **D** †*Gobius francofurtanus* and *†G. jarosi* were added to the extant species (ASDSF = 0.005118). Scale bars, average number of substitutions per site.

**Fig 6. 50% MRC Bayesian trees with posterior probabilities based on a total evidence approach (morphology and DNA) using the extant species and adding one or two fossil species.** **A** †*Eleogobius brevis* was added to the extant species (ASDSF = 0.002136). **B** †*Eleogobius gaudanti* was added to the extant species (ASDSF = 0.000477). **C** Both †*Eleogobius* species were added to the extant species (ASDSF = 0.005202). **D †***Pirskenius diatomaceus* and †*P. radoni* were added to the extant species (ASDSF = 0.017558). Scale bars, average number of substitutions per site.

**Fig 7. 50% MRC Bayesian trees with posterior probabilities based on a total evidence approach (morphology and DNA) using the extant species and adding one, two, or four fossil species.** **A** †*Paralates bleicheri* was added to the extant species (ASDSF = 0.001756). **B** †*Paralates chapelcorneri* was added to the extant species (ASDSF = 0.007668). **C** Both †*Paralates* species were added to the extant species (ASDSF = 0.004723). **D** The two species of †*Paralates* and the two species of †*Pirskenius* were added to the extant species (ASDSF = 0.010550). Scale bars, average number of substitutions per site.

**Fig 8. 50% MRC Bayesian tree with posterior probabilities based on a total evidence approach (morphology and DNA) using the extant species and all ten fossil species included in this study** (ASDSF = 0.009290). Scale bar, average number of substitutions per site.
