# Supplementary material for: An integrative phylogenetic approach for inferring relationships of fossil gobioids (Teleostei: Gobiiformes)
Source: PLoS One. 2022 Jul 8;17(7):e0271121. doi: 10.1371/journal.pone.0271121 (PMC9269936; doi:10.1371/journal.pone.0271121)
Supplement: Attachment — Submitted filename: Gierl et al Appendix-WWS.docx [file pone.0271121.s007.docx]

**Appendix: Description of fossil specimens**

**†*Carlomonnius quasigobius* Bannikov & Carnevale, 2016**

*Material*. Skeleton with otolith imprint.

*Holotype*. Museo Civico di Storia Naturale, Verona; MCSNV IGVR71187 (only known specimen).

*Stratigraphy and distribution*. Lower Eocene of Italy (upper Ypresian; Monte Bolca, Pesciara cave site), 56–47.8 Ma.

*Short description*. Vertebrae number 24 (10 abdominal, 14 caudal); dorsal fin continuous with seven spines and 11 rays, pterygiphore formula 2-3111 (for the spines); anal fin with one spine and seven rays; three anal fin pterygiophores inserting before the haemal spine of the first caudal vertebra; endopterygoid present; five branchiostegals.

*Remarks*. Based on these characters Bannikov and Carnevale [1] refrained from assigning *C. quasigobius* to a gobioid family and left it incertae sedis among Gobioidei.

*Reference*. Bannikov & Carnevale [1].

**†*Eleogobius brevis* (Agassiz, 1839)**

*Material*. Skeletons, partially with otoliths *in situ*.

*Holotype*. not assigned (see [2] Tome IV Atlas, Tab. 32, Fig. 2–4).

*Stratigraphy and distribution*. Lower-Middle Miocene of Austria (Eibiswald), Germany (Öhningen, Rhinegraben), Slovakia (Závod), 23–11.6 Ma.

*Short description.* 10 to 12 abdominal and 15 to 17 (18?) caudal vertebrae; first dorsal fin with six spines; second dorsal fin with one spine 10 to 11 rays; anal fin with one spine and eight to nine rays; six branchiostegals; palatine T-shaped; endopterygoid absent; otolith rectangular with broad posterodorsal projection, small ventral projections anteriorly and posteriorly.

*Reference*. Gierl & Reichenbacher [3].

**†*Eleogobius gaudanti* Gierl & Reichenbacher, 2015**

*Material*. Skeletons, partially with otoliths *in situ*.

*Holotype*. Naturhistorisches Museum Wien; NMW 1857 XXVII 7 (with otoliths in situ)

*Stratigraphy and distribution*. Uppermost lower Miocene of Germany (Illerkirchberg), c. 17 Ma.

*Short description*. 10 abdominal and 16 to 17 caudal vertebrae; first dorsal fin with 6 spines; second dorsal fin with one spine and nine to ten rays; anal fin with one spine and ten to 11 rays; six branchiostegals; palatine T-shaped; endopterygoid absent; otolith rectangular with posterodorsal and anteroventral projection.

*Remarks*. The authors proposed, based on the combination of characters, for this genus to be „in between“ 6brG and 5brG.

*Reference*. Gierl & Reichenbacher [3].

**†*Gobius francofurtanus* Koken, 1891**

*Material*. Skeletons, partially with otoliths *in situ*.

*Holotype*. see Koken [4] (132–133, Pl. VI, Figs. 7–7a; otolith only); better picture of same otolith in Malz ([5], tab. 4, fig. 51), here designated as lectotype with number Senckenberg-Museum Frankfurt SMF P.1956b; fate currently unknown.

*Stratigraphy and distribution*. Lower Miocene of Germany (Mainz Basin), 23–15.9 Ma.

*Short description*. 10 to 11 abdominal and 16 to 18 caudal vertebrae; first dorsal fin with six spines; pterygiophore formula 3-22110; second dorsal fin with one spine and 10 to 11 rays; anal fin with one spine and 9 to 10 rays; five branchiostegals; palatine T-shaped; no endopterygoid; otolith rectangular with big projections at the ventral corners; dorsal corners with slightly smaller projections; sulcus centred.

*Remarks*. Probably a member of the Gobiidae.

*Referencey*: Weiler [6]; Gierl [7]

**†*Gobius jarosi* Přikryl & Reichenbacher, 2018**

*Material*. Skeletons.

*Holotype*. Moravian Museum in Brno; MZM Ge25032a and b.

*Stratigraphy and distribution*. Early Miocene of the Czech Republic and Poland, 19.1–20.4 Ma.

*Short description*. Vertebral number of 11 abdominal and 16–17 caudal vertebrae; first dorsal fin with six spines and pterygiophore formula 3-22110; second dorsal with one spine and 12 rays; anal fin has one spine and 11 rays; two anal pterygiophores anterior to first hemal spine. T-shaped palatine; endopterygoid absent; five branchiostegals. The latter characters place the species within the 5brG. The presence of the postmaxillary process on the premaxilla; the D1 pterygiophore pattern; the vertebra number; two or one epural; and the two preacaudal anal pterygiophores led Reichenbacher et al. [8] to the assignment of *G. jarosi* to the Gobiidae.

*Remarks*. Based on the combination of vertebra number, ray number in the second dorsal fin, and the D1 pterygiophore formula, this new fossil species was assigned to the genus *Gobius*.

*References*. Reichenbacher et al. [8]; Kovalchuk et al. [9].

**†*Lepidocottus aries* (Agassiz, 1839)**

*Material*. Skeletons, partially with otoliths *in situ*.

*Holotype*. not assigned and lost (see [2] Tome IV Atlas, Tab. 18, Fig. 3; apparently never printed by mistake).

*Stratigraphy and distribution.* Upper Oligocene of France (Aix-en-Provence; Alpes-de-Haute-Provence), 27.8–23 Ma.

*Short description.* Vertebral number of 10 abdominal and 15–16 caudal vertebrae; first dorsal fin with six spines; corresponding pterygiophore formula 4-22110; second dorsal fin with one spine and ten rays; anal fin with one spine and eight to nine rays; three or four anal pterygiophores anterior to first hemal spine. L-shaped palatine; endopterygoid absent; six branchiostegals. Otolith rectangular longer than high with sulcus shifted anteriorly.

*Remarks*. Those features together with D1 pterygiophore formula, squamation, and reduced number of principle caudal rays (13) led Gierl et al. [10] to the conclusion that *Lepidocottus* can be placed within the Butidae.

*References*. Gierl et al. [10]; Gaudant [11].

**†*Paralates bleicheri* Sauvage, 1883**

*Material*. Skeletons.

*Holotype*. Naturhistorisches Museum Basel, NMB Ruf. 15b (neotype designated by Gaudant [12]; type material lost)

*Stratigraphy and distribution*. Lower Oligocene of France (Upper Alsace, Rouffach), 33.9–27.8 Ma.

*Short description*. 12 abdominal and 17 to 18 caudal vertebrae; first dorsal fin with seven spines; second dorsal fin with one spine and 8 to 10 rays; anal fin with one spine and 8 to 10 rays; postmaxillary process on premaxilla present.

*Remarks*. Probably belongs in the 6brG clade.

*References*. Gaudant [12]; Gierl & Reichenbacher [13].

**†*Paralates chapelcorneri* Gierl & Reichenbacher, 2017**

*Material*. Skeletons.

*Holotype*. Natural History Museum, London; NHMUK PV P 59785 (part and counterpart)

*Stratigraphy and distribution*. Upper Eocene of the Isle of Wight (UK), 37.8–33.9 Ma.

*Short description*. 12 abdominal and 17 to 19 caudal vertebrae; first dorsal fin with seven spines; pterygiophore formula 3-2121100; second dorsal fin with one spine and 9 to 10 rays; anal fin with one spine and eight to nine rays; postmaxillary process on premaxilla present; long caudal peduncle; six branchiostegals.

*Remarks*. These features of †*Paralates* rather speak for a placement within the 6brG grade. However, it is not possible to assign it to a family with certainty.

*References*. Gaudant and Quayle [14]; Gierl and Reichenbacher [13].

**†*Pirskenius diatomaceus* Obrhelová, 1961**

*Material*. Skeletons.

*Holotype*. Národní Museum Prague, NMP PC 2769.

*Stratigraphy and distribution*. Lower Oligocene of the Czech Republic (Knížecí), 33.9–27.8 Ma.

*Short description*. 11 abdominal and 16–17(18) caudal vertebrae; first dorsal fin with seven spines; D1 pterygiophore formula 3-122110 and 12210; second dorsal fin with one spine and nine to ten rays; anal fin with one spine and nine to ten rays; precaudal anal pterygiophores 2–3; palatine with short ethmoid process; endopterygoid present; seven branchiostegals.

*Remarks*. Together with †*Pirskenius radoni* (see below), the species is placed in the extinct family †Pirskeniidae, which was reconstructed as sister to Thalasseleotridae + 5brG in Reichenbacher et al. [15].

*References*. Obrhelová [16]; Přikryl [17]; Reichenbacher et al. [15].

**†*Pirskenius radoni*** **Přikryl, 2014**

*Material*. Skeletons.

*Holotype*. Museum of Teplice; MT PA1480.

*Stratigraphy and distribution*. Lower Oligocene of the Czech Republic (Central Bohemian Uplands), 33.9–27.8 Ma.

*Short description*. 12 abdominal and 16 caudal vertebrae; first dorsal fin with seven spines; D1 pterygiophore formula 4-32110; second dorsal fin with one spine and eight rays; anal fin with one spine and nine rays; precaudal anal pterygiophores 4; palatine „not exactly L-shaped“ (Reichenbacher et al. [15], p. 21); endopterygoid present; seven branchiostegals.

*Remarks*. Together with †*Pirskenius diatomaceus* (see above), the species is placed in the extinct family †Pirskeniidae, which was reconstructed as sister to Thalasseleotridae + 5brG in Reichenbacher et al. [15].

*References*. Přikryl [17]; Reichenbacher et al. [15].

**References**

1.	Bannikov AF, Carnevale G. †*Carlomonnius quasigobius* gen. et sp. nov.: the first gobioid fish from the Eocene of Monte Bolca, Italy. Bulletin of Geosciences. 2016;91(1):13–22. Epub Dec 2, 2015. doi: 10.3140/bull.geosci.1577. PubMed PMID: WOS:000372546600002.

2.	Agassiz L. Recherches sur les Poissons fossiles. Tome IV. Neuchâtel: Petitpierre; 1833–1843. 348 p.

3.	Gierl C, Reichenbacher B. A new fossil genus of Gobiiformes from the Miocene characterized by a mosaic set of characters. Copeia. 2015;103(4):792–805. Epub Nov 12. doi: 10.1643/ci-14-146. PubMed PMID: WOS:000366679700006.

4.	Koken E. Neue Untersuchungen an tertiären Fisch-Otoloithen II. Zeitschrift der deutschen geologischen Gesellschaft. 1891;43:77–170.

5.	Malz H. Vergleichend-morphologische Untersuchungen an aquitanen Fisch-Otolithen aus dem Untergrund von Frankfurt am Main. Senckenbergiana lethaea. 1978;59(4/6):441–81.

6.	Weiler W. Die Fischfauna des Tertiärs im oberrheinischen Graben, des Mainzer Beckens, des unteren Maintals und der Wetterau, unter besonderer Berücksichtigung des Untermiozäns. Abhandlungen der Senckenbergischen Naturforschenden Gesellschaft. 1963;504:1–75.

7.	Gierl C. Articulated gobioid skeletons from the Frankfurt-Formation (Lower Miocene). München: Ludwig-Maximilians-Universität München; 2012.

8.	Reichenbacher B, Gregorová R, Holcová K, Šanda R, Vukić J, Přikryl T. Discovery of the oldest *Gobius* (Teleostei, Gobiiformes) from a marine ecosystem of Early Miocene age. Journal of Systematic Palaeontology. 2018;16(6):493–513. Epub May 2, 2017. doi: 10.1080/14772019.2017.1313323. PubMed PMID: WOS:000424810000002.

9.	Kovalchuk O, Świdnicka E, Stefaniak K. A new record of *Gobius jarosi* (Teleostei, Gobiidae) from the Early Miocene of Poland with inference to paleogeography and palaeoecology of the Carpathian Basin. Historical Biology. 2019;31(10):1394–401. Epub Mar 28, 2018. doi: 10.1080/08912963.2018.1457032.

10.	Gierl C, Reichenbacher B, Gaudant J, Erpenbeck D, Pharisat A. An extraordinary gobioid fish fossil from southern France. PloS one. 2013;8(5):e64117. Epub May 15. doi: 10.1371/journal.pone.0064117. PubMed PMID: 23691158; PubMed Central PMCID: PMCPMC3655028.

11.	Gaudant J. Présence du genre *Lepidocottus* Sauvage, 1875 (Teleostei, Gobioidei) dans l'Oligocène inférieur des environs de Céreste (Alpes-de-Haute-Provence, France). Geodiversitas. 2015;37(2):229–35. doi: 10.5252/g2015n2a4. PubMed PMID: WOS:000357401300004.

12.	Gaudant J. Sur la présence de Gobiidae (Poissons téléostéens) dans l'Oligocène inférieur de Rouffach (Haut-Rhin). Sci Géol Bull. 1979;32(3):131–7. doi: 10.3406/sgeol.1979.1560.

13.	Gierl C, Reichenbacher B. Revision of so-called *Pomatoschistus* (Gobiiformes, Teleostei) from the late Eocene and early Oligocene. Palaeontologia Electronica. 2017;20.2.33A:1–17. doi: 10.26879/721. PubMed PMID: WOS:000405188500014.

14.	Gaudant J, Quayle WJ. New palaeontological studies on the Chapelcorner fish bed (Upper Eocene, Isle of Wight). Bull Br Mus nat Hist (Geol). 1988;44(1):15–39.

15.	Reichenbacher B, Přikryl T, Cerwenka AF, Keith P, Gierl C, Dohrmann M. Freshwater gobies 30 million years ago: New insights into character evolution and phylogenetic relationships of †Pirskeniidae (Gobioidei, Teleostei). PloS one. 2020;15(8):e0237366. Epub Aug 24. doi: 10.1371/journal.pone.0237366. PubMed PMID: WOS:000565550400035.

16.	Obrhelová N. Vergleichende Osteologie der tertiären Süsswasserfische Böhmens (Gobioidei). Sbornik Paläont. 1961;26:103–92.

17.	Přikryl T. A new species of the sleeper goby (Gobioidei, Eleotridae) from the České Středohoří Mountains (Czech Republic, Oligocene) and analysis of the validity of the family Pirskeniidae. Paläontol Z. 2014;88(2):187–96. Epub Jun 28, 2013. doi: 10.1007/s12542-013-0188-y.
