# Supplementary material for: An integrative phylogenetic approach for inferring relationships of fossil gobioids (Teleostei: Gobiiformes)
Source: PLoS One. 2022 Jul 8;17(7):e0271121. doi: 10.1371/journal.pone.0271121 (PMC9269936; doi:10.1371/journal.pone.0271121)
Supplement: Attachment — Submitted filename: Response to reviewers.docx [file pone.0271121.s008.docx]

Reviewer 1:

Reviewer #1: Dear authors, This is an interesting article with a novel approach and incorporating otolith research for the first time in a total evidence phylogenetic analysis. My congratulations to your work.

There are, however, a few issues that need to be addressed in my view before this article can be published. I have made some comments in the main text and the supplementary data as attached, but there are a few principle issues which I would like to bring to your attention as follows:

1. I am lacking a discussion about the state of the characters used, i.e., plesiomorphic or apomorphic and polarity. This is particularly so for the otolith characters while for the other characters possibly references could be cited. In general, however, this is my most concerning issue.

We have added the requested information to the Suppl Table Part B by providing a new column in which we explain how the plesiomorphic character state was determined. If such information was available from the literature, we indicate the corresponding literature source in this column. If no such information was available from previous work, we have used the outgroup and for some characters additionally *Rhyacichthys* to define it. Each plesiomorphic character state is marked by a grey shading in Part B of the Suppl Table. We have complemented the Method part of the revised manuscript accordingly.

2. Did the phylogenetic analyses reflect plesiomorphic versus apomorphic character states and if so, how?

This can best be assessed using the tree based only on the morphological data of the extant species (Fig 4B). The clades for which apomorphies are known (5brG, Thalasseleotrididae, Thalasseleotrididae+5brG, Eleotridae) are resolved in this tree. Groups of which apomorphies are yet unknown are recovered as paraphyletic (Butidae, Odontobutidae, Rhyacichthyidae). We have added a notice about this in the first paragraph of the Discussion.

3. The phylogeny collapsed when all fossil taxa where included. I find this intriguing and the discussion to this aspect way too short, while the technical phylogenetic discussion from the various runs is rather exhaustive. Definitely, this aspect requires more attention and consideration and discussion than you provided in the manuscript.

We added some additional thoughts in the last paragraph of the discussion.

4. Much reference is made to the earliest gobioid on the fossil record- Carlomonnius quasigobius. A very detailed anatomical and phylogenetic analysis was provided by Bannikov & Carnevale (2016) who described it resulting in not placing it in a specific family because of its unique mixture of plesiomorphic and apomorphine characters. In this article it is now based in Butidae based on your phylogenetic modeling alone without discussion of how the results of Bannikov & Carnevale would relate. This needs to be considered and discussed in more detail because of its phylogenetic impact.

We added some additional thoughts in the discussion. We also explain that we cannot exclude that C. quasigobius represents an extinct clade with a combination of plesiomorphic (e.g. continuous dorsal fin, 24 (10+14) vertebrae, autogenous haemal spine of the second preural centrum, first two abdominal centra shortened, first dorsal- fin pterygiophore inserting in the second interneural space) and derived characters (five branchiostegal rays, dorsal postcleithrum absent, 11 branched and segmented caudal-fin rays, four pelvic-fin rays], as described in Bannikov & Carnevale (2016).

With these aspects being carefully addressed I am of the opinion that the manuscript will become a very valuable article both in terms of the methodology applied as well as the results presented.

Thank you for the positive feedback and constructive comments. All of which have been considered in the revised version of this manuscript.

From Manuscript:

L.23: Here and later why not use the official abbreviation Ma?

“Ma” is established in the geoscientific community, while the colleagues of the biological community are not always familiar with this abbreviation. Therefore, we prefer to keep “m.y. ago”

L.33: This is still a relatively low coverage given that there are 2310 extant species and 320 extant genera.

We are aware of this issue (is also explicitly mentioned in the method part). However, our data set was sufficient to achieve a molecular ‘backbone’ that fully conforms to previous molecular work (please see following sentence of the Abstract, line 36, 37).

Would it make sense to refer to comparative publications?

We used comparative publications (see Table 1), which is indicated at the beginning of the sentence (“using both literature and newly collected data”). It is explained more in detail in the Material section.

L.46: Any rational why this should be so? There is some discussion at the end of the article, but in the abstract it should either be explained or left out.

We agree that in the abstract we can leave it out.

L.58: this is awful! Please write "sensu Thacker et al., 2015"

Ok, we changed to Thacker et al. [10]

L.59: The placement of Trichonotidae in that clade is controversial

Yes, because there are different definitions of the term “Gobiiformes”, which is indicated in our Figure 1 and also mentioned in the text. In the sentence on line 59, we refer to the “Gobiiformes sensu Thacker et al. (2015)” and according to Thacker et al. (2015), the Trichonotidae are part of the Gobiiformes.

L. 73: This is not uniformly accepted and I suggest to exclude them here.

As explained above, Trichonotidae are listed here according to the phylogeny of Thacker et al. 2015.

L.78: this would then not be necessary to highlight.

OK, done. In addition, we revised Figure 1 to include the information how Nelson et al. (2016) define Gobiiformes

Table: Gobius francofurtanus like does not belong to the genus Gobius, but is a relict from a time when Gobius was used as a junk basket for such fossil taxa. I would therefore recommend here and later to name it: "Gobius" francofurtanus.

OK, done.

L.189: don't understand the difference in these three terms. Please consider rewording. The explanatory figure shows only rectangular and rectangular/higher than long. How about simplier and more easy to understand terms such as long rectangular, high rectangular, quadratic?

OK, done.

L.191: this is not very informative. Please describe more precisely.

We changed it to perciform-like.

L.210: I am missing here or in the supplementary table a discussion about which character states are considered plesiomorphic and which apomorphic, i.e., the polarity of characters. In most cases, this can probably be resolved with referencing published articles, but not for otoliths, which is newly introduced here. So at least for those characters some detail discussion of character state and polarity is required.

We have added the plesiomorphic character states (and an explanation or reference) for each character to the supplementary Table 1. See above (point 1.)

L.602: but why is it not working when adding several fossil taxa? See discussion at end of article and comment to that.

We added some thoughts.

L.623: But how about Carlomonnius representing a fossil family like Pirskenius, or a stem Gobioidei. I am lacking a more specific discussion of how the assignment cope with the 5 derived and 5 plesimorphic characters discussed in Bannikov & Carnevale 2016.

OK, done, see above (point 4.)

L.630: these are universal characters of no specific phylogenetic value at the genus level.

True, that's where the otoliths come in... and it is clearer now in the text.

L.631: see Bradic-Milinovic et al 2019: it is not only the number of branchiostegal rays that may matter but also where they are inserted. Is this comparable among the two recorded species of Eleogobius or not?

This is a good point. Actually, the positions of the branchiostegal rays appear different in the two *Eleogobius* species. However, the available fossils are moderately well preserved, thus it is currently difficult to say whether the observable bone arrangement corresponds to the natural anatomical configuration. Nevertheless, it could be a hint, that the two *Eleogobius* species may actually represent two different genera. It is added to the text.

L.645: The figure of Micropercops swinhonis in Nolf 2013 is a drawing and not very detailed. Schwarzhans et al., 2017 contain photographs. I can see no clear resemblance of Umbra valida with Micropercops. I suggest to reword this sentance in a more careful manner. I further suggest to make reference to the late Oligocene Micropercops pomahaka in Schwarzhans et al. 2017 from New Zealand.

We have deleted the comparison to these otoliths from the manuscript as further evidence should be awaited

L.645: Stinton 1977 described it as Umbra valida and I recommend to refer to the original reference.

Not necessary any more as the corresponding text has been removed

L.665: Could this not rather be an indication that some fossil taxa do not fit into the backbone built from extant taxa because they represent extinct clades or stem taxa?

Yes. We have added this (last chapter of Discussion).

From Suppl Table-WWS:

In none of the listed 54 characters has it been noted and explained, which character state is considered plesimorphic and which apomorphic. For the majority of characters, this can possibly be deduced from published assessment. For otoliths this is new and character polarity should be discussed.

See above point 1.

From Supplementary Figures S1-S8-WWS:

Please note that the otolith of Thalasseleotris iota looks quite different from the ones figured in Schwarzhans, 2019, which includes information from a paratype.

We checked that and we found out that there has been an unfortunate error with the Thalasseleotris specimen we had received from a museum collection. This specimen was wrongly labelled and it was not Thalasseleotris, which we had not realized before. Now we have removed all data of this falsely labelled specimen (including its otolith) from our data and replaced all information by literature data (see Table 1).

From Appendix:

Not quite. Bannikov and Carnevale listed five derived and five plesiomorphic characters in a unique combination not found in any extant gobioid lineage. This should be adequately acknowledged here and correctly cited in the description and discussion thereof. Furthermore, it should be explained how the new familial allocation proposed from the ‚total evidence’ aproach deals with the individual characters and assessment by Bannikov & Carnevale. Finally, it should be mentioned that this taxon represents the first/earliest confirmed record of a gobioid.

Done.

There is more to be considered than just the number. See Bradic-Milinovic et al. 2019 why the 6 branchiostegals in the case of Elegobius may not be homologous with the 6 branchiostegals found in Eleotridae, Butidae etc.

Done.

Reviewer 2:

Reviewer #2: This paper is well-written and I appreciate the hard work done by the authors. However, several questions and concerns arose during the readings. The authors claimed that they were able to assign several of the incertae sedis fossils to family level using their total-evidence phylogeny, but in many cases the results are not solid. Also, I was not able to review the Suppl. Figs 1-8 (what I was able to download was a docx. with the figures in the main text, not Suppl. Figs 1-8), so basically, I was only able to access half of the results presented.

1. The step-wise approach (L. 229) seems to have good resolution on the results, but what’s the rationale here to this approach only? Because when multiple fossils are included in the analysis, the phylogeny collapsed (as in L. 664), which can be easily challenged by future works when more evidence and specimens are available, then, the total evidence phylogeny produced here is not a stable one, and means that you need other robust phylogeny (topology) for the ten fossil taxa (and perhaps not yours). Or, on the contrary, the morphological data are too few to reconstruct a good tree for fossils, leaving those inc. sed. still incertae sedis. If that’s so, then you are forcing the taxonomic assignment to any of the extant families while they might well belong to any of an extinct clade.

We have restructured the Result part in the revised version to better explain the rationale of our approach. Yes, more morphological data could certainly help. However, the idea of our study was also that it could serve as a (promising) starting point to carry this work on. Not to mention the hundreds of extant goby species that have more or less unknown osteology.

Yes, the fossils may represent fossil clades/families and we have added this in the revised version of the manuscript (last chapter of Discussion).

2. L. 618, you mentioned these morphological traits, but these are conflicting your assignment below! Therefore, the statement in L. 615-624 is weak, there should be morphological evidence supporting your assignment rather than your phylogenetic tree (L. 583, with low support value!) or just stating the stratigraphic occurrences. Also, if C. quasigobius really is a Butidae, then the expansion of the fossil record is also a big deal (some 10-20 Ma earlier).

Done. We have rephrased the paragraph dealing with *Carlomonnius* in the revised version of the manuscript according to these concerns.

3. In my opinion, we only have a limited number of extant goby families and that might not be ideal to place the unknown, yet to be fully discovered fossil taxa to the living scheme, as there are myriads of unknown diversity behind (as you wrote in L. 611-614). Therefore, it might not be so surprising that the results are not well resolved as all.

Yes. We have added this (last chapter of Discussion).

4. L. 218, you mentioned some molecular data came from “Thacker et al. [62]”, but it didn’t match the ref. 62 in the References.

Corrected.

5. Suppl. Table. I suggest following Sereno (2007) for character description. Sereno, C. P. 2007. Logical basis for morphological characters in phylogenetics. Cladistics 23(6): 565-587.

We did not follow this suggestion as we do not consider it necessarily an Improvement

To Editor:

1. Please ensure that your manuscript meets PLOS ONE's style requirements, including those for file naming.

2. To comply with PLOS ONE submissions requirements, please provide methods of sacrifice in the Methods section of your manuscript.

We added that all specimens originate from museum collections (as indicated in Table 1) and no specimens were sacrificed for this work.

3. In your manuscript, please provide additional information regarding the specimens used in your study. Ensure that you have reported specimen numbers and complete repository information, including museum name and geographic location.

The requested information is provided in Table 1

4. We note that you have provided funding information that is not currently declared in your Funding Statement. However, funding information should not appear in the Acknowledgments section or other areas of your manuscript. We will only publish funding information present in the Funding Statement section of the online submission form. Please remove any funding-related text from the manuscript and let us know how you would like to update your Funding Statement.

Done

5. In your Data Availability statement, you have not specified where the minimal data set underlying the results described in your manuscript can be found. PLOS defines a study's minimal data set as the underlying data used to reach the conclusions drawn in the manuscript and any additional data required to replicate the reported study findings in their entirety. All PLOS journals require that the minimal data set be made fully available. For more information about our data policy, please see http://journals.plos.org/plosone/s/data-availability.

Upon re-submitting your revised manuscript, please upload your study’s minimal underlying data set as either Supporting Information files or to a stable, public repository and include the relevant URLs, DOIs, or accession numbers within your revised cover letter.

Done

All data matrices and trees are available on figshare (<https://figshare.com/s/ed10b9a5ac382f856a20>).
