# Supplementary material for: An integrative phylogenetic approach for inferring relationships of fossil gobioids (Teleostei: Gobiiformes)
Source: PLoS One. 2022 Jul 8;17(7):e0271121. doi: 10.1371/journal.pone.0271121 (PMC9269936; doi:10.1371/journal.pone.0271121)
Supplement: Attachment — Submitted filename: Response to reviewers R2.docx [file pone.0271121.s010.docx]

Review Comments to the Author

**Reviewer #1:** Thank you for considering all the comments and suggestions made. In my opinion,

the manuscript reads very well and conclusive now.

Thank you for the positive evaluation.

Two minor aspects I would like to bring to your attention before final submission and publication:

1.- In the abstract some sort of reference should be made to the problems concerning the tree collapsing when including all fossil taxa.

DONE

2.- In the phylogenetic analysis you mention the problem of the tree collapsing with all fossil data for the first time. Later, in the chapter Discussion you have outlined your thoughts to this effect, which in my opinion are quite comprehensive. You should refer to this discussion where you mention the effect first in the text as a guidance for the reader.

Looking forward to see you study published and best wishes,

DONE

Reviewer #2: Dear authors,

After reviewing the revised manuscript, I judge that all my previous comments have been

addressed adequately. My congratulations to your work.

Thank you for the positive evaluation.
